# Genomic Characterization of Antimicrobial-Resistant Salmonella enterica in Duck, Chicken, and Pig Farms and Retail Markets in Eastern China

**DOI:** 10.1128/spectrum.01257-22

**Published:** 2022-09-01

**Authors:** Biao Tang, Mohammed Elbediwi, Reshma B. Nambiar, Hua Yang, Jiahui Lin, Min Yue

**Affiliations:** a State Key Laboratory for Managing Biotic and Chemical Threats to the Quality and Safety of Agro-products, Institute of Agro-product Safety and Nutrition, Zhejiang Academy of Agricultural Sciences, Hangzhou, China; b Department of Veterinary Medicine, Institute of Preventive Veterinary Sciences, Zhejiang University College of Animal Sciences, Hangzhou, Zhejiang, China; c Hainan Institute of Zhejiang University, Sanya, China; d State Key Laboratory for Diagnosis and Treatment of Infectious Diseases, National Clinical Research Center for Infectious Diseases, National Medical Center for Infectious Diseases, The First Affiliated Hospital, College of Medicine, Zhejiang University, Hangzhou, China; e Zhejiang Provincial Key Laboratory of Preventive Veterinary Medicine, Hangzhou, China; University of Maryland Eastern Shore

**Keywords:** *Salmonella*, food animals, antimicrobial resistance, genomic characterization, virulence factors

## Abstract

Antimicrobial-resistant Salmonella enterica poses a significant public health concern worldwide. However, the dissemination of Salmonella enterica among food animals in eastern China has not been fully addressed. Here, we demonstrated the antimicrobial resistance (AMR) patterns and the whole-genome characterization of 105 S. enterica isolates from 1,480 fecal samples and anal swabs collected from 22 different farms (chickens, ducks, and pigs) and two live animal markets located in Zhejiang and Fujian Provinces in eastern China in 2019. The prevalence of isolates in duck farms (19.17%, 23/120) was statistically significantly higher (*P < *0.001) than that in chicken farms (6.61%, 37/523) and pig farms (3.50%, 7/200). Among these isolates, 75.26% (79/105) were multidrug resistant, with the highest rates of resistance to tetracycline (76.20%) and ampicillin (67.62%) and the lowest resistance rate to meropenem (0.00%). The serotypes were consistent with sequence types and were closely related to the sampling animal species and sites. S. enterica serotype Kentucky (20.95%, 22/105) was the most frequent serotype and harbored more AMR patterns and genes than others. Furthermore, IncFII(S) and IncHI2 were the most prevalent replicons. A total of 44 acquired AMR genes were found. Among those genes, *aac(6′)-Iaa*, *bla*_TEM-1B_, *floR*, *dfrA14*, *fosA7*, *mph*(A), *qnrS1*, *sul1*, *tet*(A), and *ARR-3* were the dominant AMR genes mediating the AMR toward aminoglycosides, β-lactams, phenicol, trimethoprim, fosfomycin, macrolide, quinolone, sulfonamides, tetracycline, and rifampin, respectively. The consistency of acquired AMR genes with AMR phenotypes for ampicillin, ceftiofur, ceftazidime, meropenem, sulfamethoxazole-trimethoprim, and tetracycline was >90%. Together, our study highlights the application of whole-genome sequencing to assess veterinary public health threats.

**IMPORTANCE** Public health is a significant concern in China, and the foodborne pathogen Salmonella, which is spread via the animal-borne food chain, plays an important role in the overall disease burden in China annually. The development of advanced sequencing technologies has introduced a new way of understanding emerging pathogens. However, the routine surveillance application of this method in China remains in its infancy. Here, we applied a pool of all isolates from the prevalence data in Zhejiang and Fujian for whole-genome sequencing and combined these data with the cutting-edge bioinformatic analysis pipeline for one-step determination of the complete genetic makeup for all 105 genomes. The illustrated method could provide a cost-effective approach, without labor-intensive laboratory characterization, for predicting serotypes, genotypes, plasmid types, antimicrobial resistance genes, and virulence genes, and thus would provide essential knowledge for emerging pathogens. Our findings and perspectives are essential for delivering updated knowledge on foodborne pathogens in an understudied region in China.

## INTRODUCTION

Salmonellosis, which is caused by Salmonella enterica, is a significant global foodborne disease of humans and livestock that can cause enteric gastroenteritis (1, 2). Salmonella is considered one of the most widespread pathogenic foodborne bacteria in Chinese food commodities (3), and it poses a severe threat to food safety and public health. It is estimated that 70% to 80% of foodborne bacterial outbreaks are attributable to Salmonella infections in China (4, 5). Nontyphoid Salmonella spp. cause 9.874 million gastroenteritis cases annually, and 91.5% of these cases are caused by food transmission (4). Food animals, especially poultry and pigs, are believed to be the primary reservoirs for a large number of different Salmonella serotypes (6, 7). Poultry is one of the most common animal foods in China, and it was found that 52.2% of retail chicken carcasses were contaminated with Salmonella (5). Additionally, several previous studies reported salmonellosis outbreaks in China linked to poultry, chicken, and ducks (8–10) and to pork (11–13).

Antimicrobial resistance (AMR) has been recognized as a global health problem for decades. Major world health organizations have now elevated it to one of the top health challenges facing the 21st century (14, 15). Some of its causes are the overuse and misuse of antibiotics, which have accelerated the selection pressure for the accumulation of antimicrobial-resistant bacterial species, especially those belonging to the *Enterobacteriaceae* family (16). Indeed, the emergence of multidrug-resistant (MDR) Salmonella is a primary global food safety concern, and infections caused by MDR Salmonella can increase morbidity and mortality (17–19).

Recent advances in sequencing technologies have encouraged whole-genome sequencing (WGS) in routine epidemiological investigations, especially for foodborne bacterial species, including Salmonella. Significantly, WGS can facilitate early detection of emerging threats that could result in severe human and animal infections (20, 21), providing a comprehensive toolkit for food safety and public health.

In this study, we investigated the prevalence of different S. enterica serotypes isolated from chicken, duck, and pig farms, as well as from live animal markets in Zhejiang and Fujian Provinces, eastern China. In addition, we performed antimicrobial susceptibility testing and, importantly, genomic characterization. The study evaluated the potential relationship between the food animals, serotypes, AMR phenotypes, AMR determinants, virulence factors, and accompanying plasmids found in the S. enterica isolates under investigation. Here, as part of the surveillance program, we provided a framework for routine genomic sequencing combined with an advanced analytic approach, which could accelerate the detection of food safety risks at the beginning of the animal food chain.

## RESULTS

### Prevalence and geographical distribution of Salmonella serovars.

**FIG 1:**
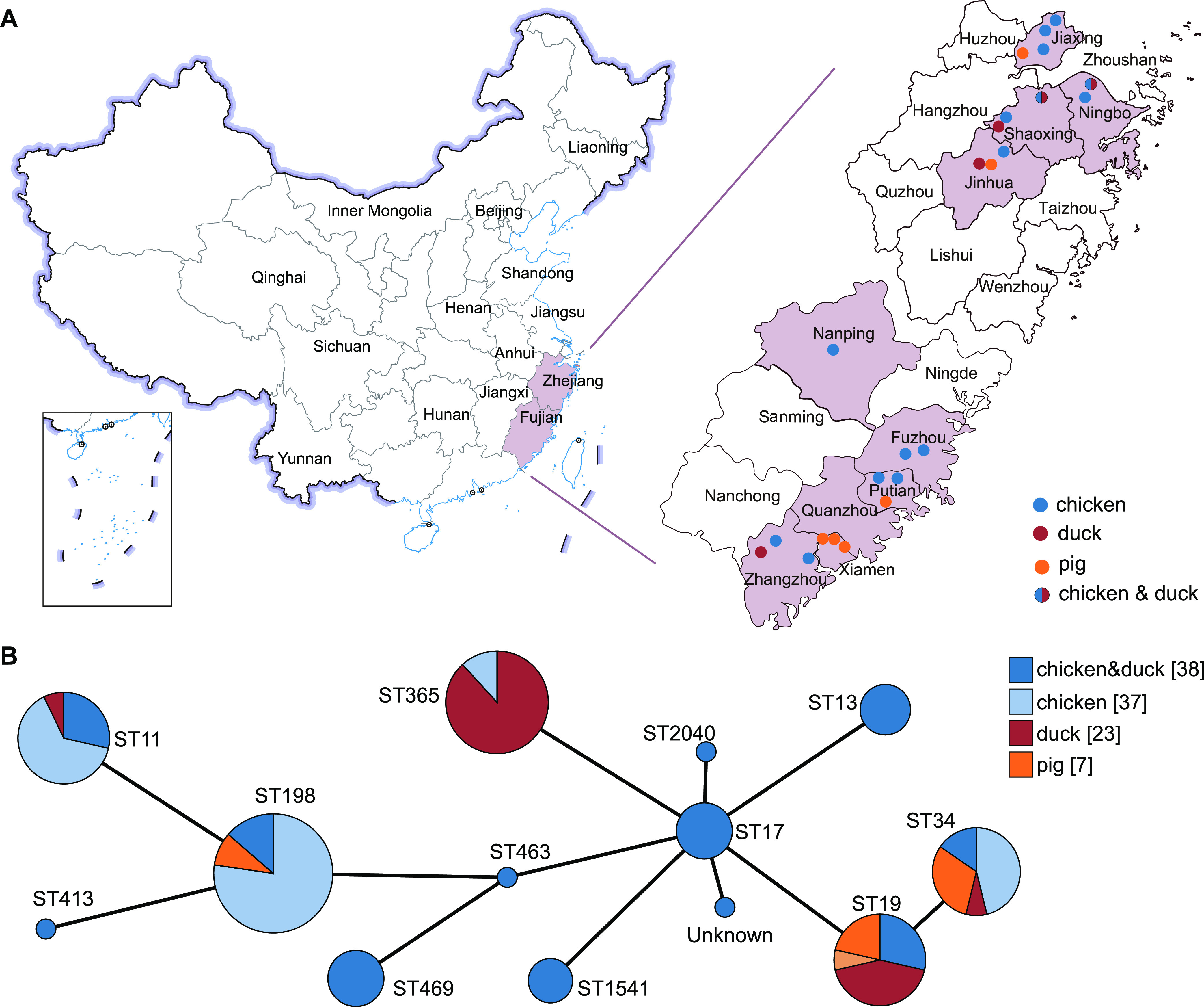
Geographical distribution of the sampling areas in Zhejiang and Fujian Provinces, China, and minimum spanning tree of Salmonella strains based on MLST. (A) The sampling sources of the chickens, ducks, and pigs are denoted in blue, dark red, and orange circles, respectively. Zhejiang and Fujian Provinces in this study are shaded in light pink. (B) Each node represents a single ST. The size of a node is proportional to the number of isolates. The lengths of branches between each node are proportional to the number of different alleles between the two nodes.

A total of 105 (7.09%) Salmonella isolates were recovered from 1,480 samples from chickens, ducks, and pigs (feces or anal swab) (Fig. 1). These 105 Salmonella isolates were identified in only 11 out of 22 farms and from two mixed (chicken and duck) live animal markets in five cities in Fujian Province and four cities in Zhejiang Province (Fig. 1). The prevalence of Salmonella in the chicken farms ranged from 0% to 27.5% (11/40), and the highest prevalence of Salmonella was on the F17 farm in Xiamen city, Fujian. The F22 farm in Zhangzhou city (16/40, 40%) showed the highest prevalence of Salmonella among all 22 farms. Salmonella isolates of pig origin were only detected in two farms, F7 in Jiaxing city, Zhejiang (1/40, 2.5%) and F14 in Xiamen city, Fujian (6/40, 15%) (Fig. 1; see also Table S1 in the supplemental material). The isolated prevalence of the 24 sampling areas from different sources was diverse (see Fig. S1). Based on the sources of the samples, a higher frequency of Salmonella isolates was obtained from chicken samples (55%, 37/67), followed by duck samples (34.3%, 23/67). However, the prevalence of isolates in duck farms (19.17%, 23/120) was statistically higher (*P* < 0.001) than that in chicken farms (6.61%, 37/523) and pig farms (3.50%, 7/200) (see Tables S1 and S2).

**FIG 2:**
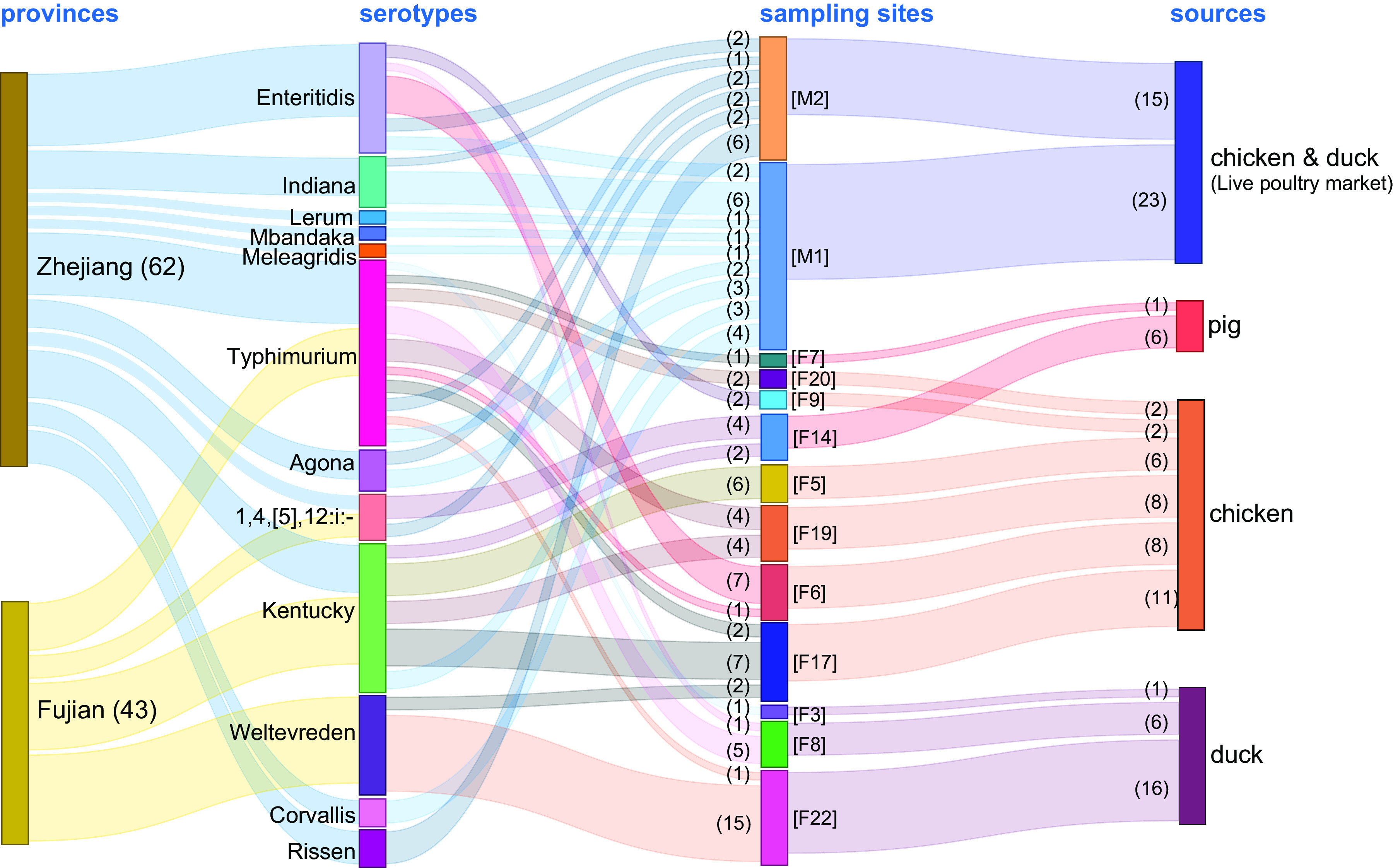
Sankey diagram combining the provinces, serotypes, farms, and sampling sources for 105 Salmonella isolates. The diameter of a line is proportional to the number of isolates from the indicated source, which is also shown in parentheses on the right. Line colors correspond to provinces and sampling sources. M1 and M2 refer to live animal markets, and other labels refer to animal farms.

As both traditional and *in silico* serotyping methods gave identical results, we found that these 105 isolates belonged to 12 distinct serotypes. The most frequent serotype was serotype Kentucky (20.95%, 22/105), followed by Typhimurium (20.00%; 21/105), and Weltevreden (16.19%, 17/105) (Fig. 2). The results also demonstrated that serotypes Kentucky and Weltevreden were the most frequently represented serotypes in chickens and ducks in this study, respectively (Fig. 2; see also Table S3). Four serotypes were isolated in Fujian Province, of which serotype Weltevreden was isolated only in Fujian Province. There were 11 serotypes isolated in Zhejiang Province, among which serotypes Enteritidis, Indiana, Lerum, Mbandaka, Meleagridis, Agona, Corvallis, and Rissen were only isolated in Zhejiang Province, mainly due to the large number of serotypes found in the live poultry markets.

### Antimicrobial resistance phenotype.

**FIG 3:**
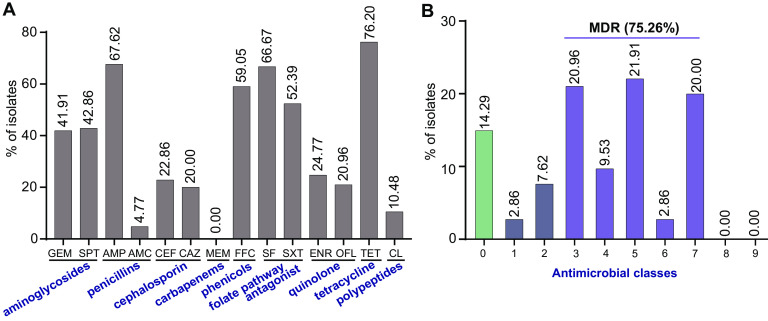
AMR rates of Salmonella isolates. (A) The prevalence of antimicrobial-resistant isolates for each individual drug. (B) Distribution of MDR isolates.

**FIG 4:**
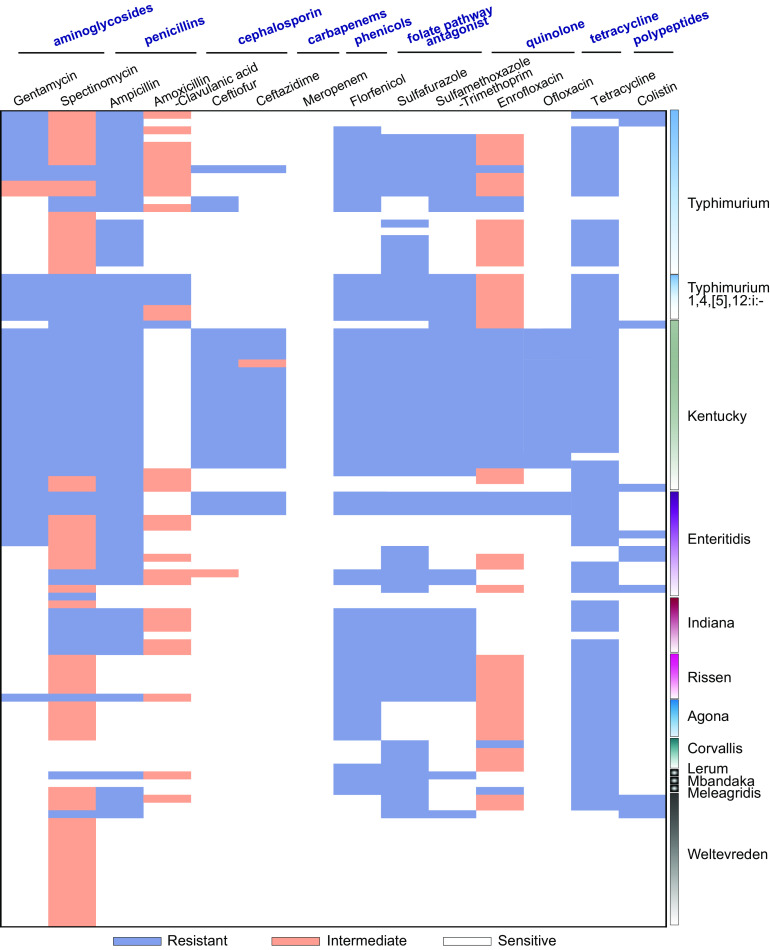
AMR patterns of Salmonella isolates. Orange indicates intermediate resistance, while blue-gray indicates resistant phenotypes.

The results showed that 76.20% (80/105) and 67.62% (71/105) of the isolates were resistant to tetracycline and ampicillin, respectively. In contrast, all isolates were susceptible to meropenem (Fig. 3A). Thirty AMR profiles were identified in the 105 Salmonella isolates (see Table S4). Of these, 75.26% (79/105) of studied isolates were MDR (i.e., could resist ≥3 antimicrobial classes) (Fig. 3B). We also noted that 90 isolates (85.71%) were resistant to at least one antimicrobial agent, whereas 15 isolates (14.29%) were susceptible to all tested antimicrobials. Our study determined that the most common AMR profile was gentamicin (GEN)-spectinomycin (SPT)-ampicillin (AMP)-ceftiofur (CEF)-ceftazidime (CAZ)-florfenicol (FFC)-sulfafurazole (SF)-trimethoprim with sulfamethoxazole (SXT)-enrofloxacin (ENR)-ofloxacin (OFL)-tetracycline (TET) (20 isolates, 19.05%) (see Table S4). Importantly, 18/22 (81.8%) *S.* Kentucky, 3/14 (21.4%) S. Enteritidis, and 3/27 (11.1%) *S.* Typhimurium isolates were resistant to a third-generation cephalosporin (ceftiofur), which was of great concern. Interestingly, 11 isolates (4 S. Enteritidis, 2 *S.* Typhimurium, 3 *S.* Weltevreden, and 1 *S.* Kentucky) were colistin resistant (Fig. 4). At the farm level, we found that the most prevalent antimicrobial-resistant isolates were identified in two chicken farms, F17 (7/11) and F5 (3/5), in addition to one pig farm, F19, with 4/10 (40%) that could resist 11 different antibiotics belonging to seven antimicrobial classes (see Fig. S2). We also noticed that the isolates that belonged to the *S.* Kentucky serotype obtained from F17, F19, and F5 showed a resistance profile toward seven antimicrobial classes, followed by *S.* Typhimurium, which was obtained from F17 and F19 and showed resistance to six antimicrobial classes (Fig. 4 and Fig. S2). Interestingly, isolates obtained from the same farm showed different AMR patterns. Isolates resistant to florfenicol, sulfamethoxazole-trimethoprim, spectinomycin, ampicillin, sulfafurazole, tetracycline, gentamicin, enrofloxacin, ceftiofur, ceftazidime, and ofloxacin were isolated from F5, F6, F8, F14, F17, F19, M1, and M2. In contrast, in some farms, such as duck farm F22, the AMR levels were low. In general, no farm profoundly affected the AMR analysis in this study (see Fig. S2).

### Multilocus sequence typing patterns.

The most prevalent sequence type was ST198 (20.95%, 22/105), detected in *S*. Kentucky isolates that originated from chickens, followed by ST365 (16.19%, 17/105), which was chiefly detected in *S.* Weltevreden isolates recovered from ducks. ST19, mainly seen in *S.* Typhimurium, showed a prevalence of 13.33% (14/105) (Fig. 1; see also Fig. S3). We also noticed that each serotype was associated with one ST, except for *S.* Typhimurium (associated with ST19 and ST34) and *S.* Indiana (associated with ST17 and ST2040).

### Plasmid profiles.

**FIG 5:**
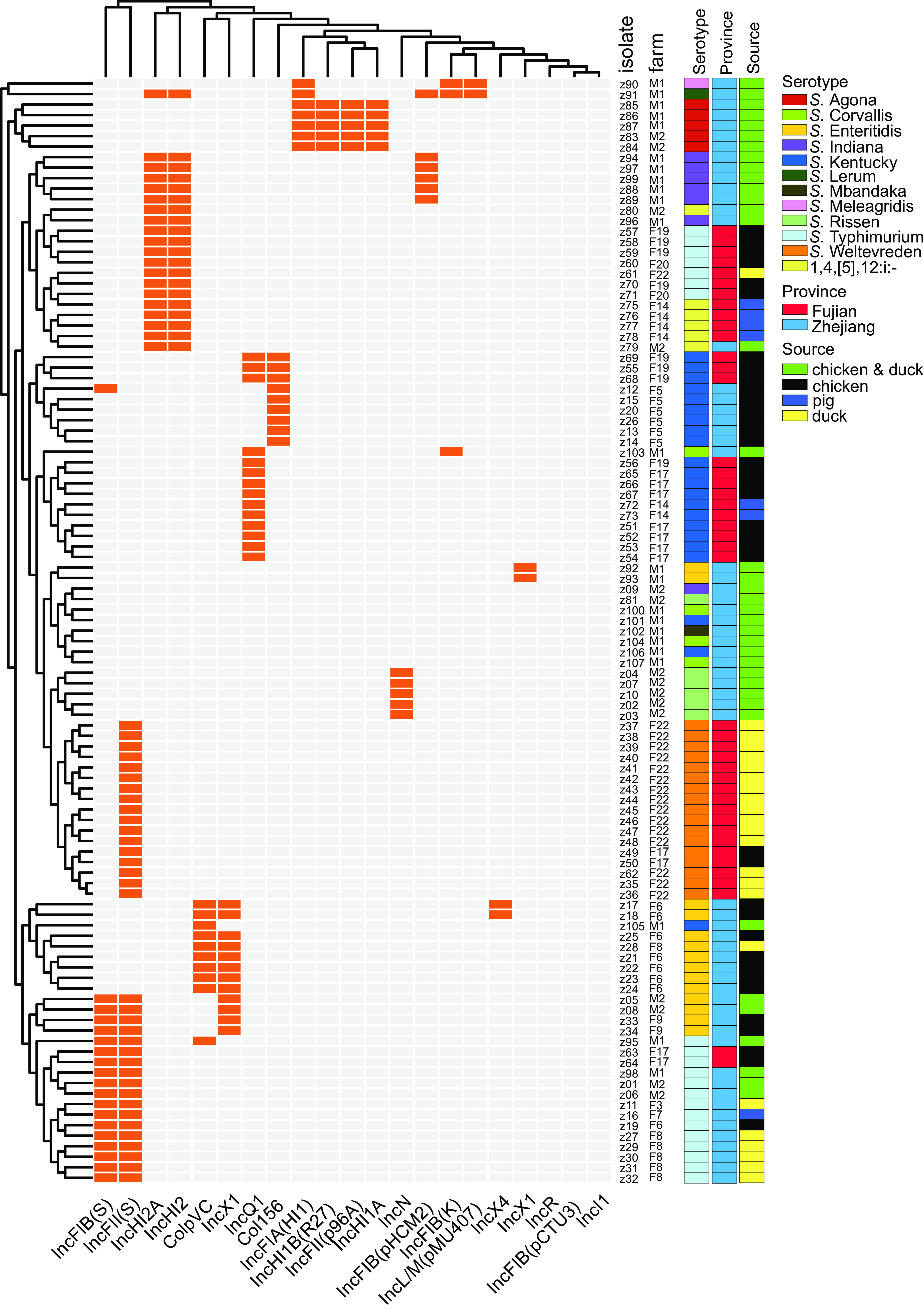
Dendrogram of a hierarchical clustering heat map of the 105 isolates and 19 plasmid replicons. The heat map shows the plasmid replicon profiles for the studied isolates. The *y* axis shows the isolate ID numbers, and the *x* axis shows the identified plasmid replicon. Orange cells, plasmid present; gray cells, plasmid absent.

WGS showed 19 different plasmid replicons in the recovered Salmonella isolates. The most significant number of plasmid replicon types was six replicons, and they were detected in *S.* Typhimurium and monophasic variant isolates. The most prevalent plasmid replicon type among all plasmids was IncFll(S), which was found to be harbored by 51% (14/27) *S.* Typhimurium and monophasic variant, 100% (17/17) *S.* Weltevreden, and 21.4% (3/14) S. Enteritidis isolates. Followed by IncHI2 and IncHI2A plasmids, which were also harbored by 49% (13/27) *S.* Typhimurium and monophasic variant, one *S.* Lerum, and 85.7% (6/7) *S.* Indiana isolates (Fig. 5). Interestingly, IncFll(S), IncHI2, and IncHI2A plasmids were harbored by different isolates originating from chickens, ducks, and pigs from both provinces, which indicated wide dissemination of these plasmids among the other hosts and across distinct geographic regions.

### Antimicrobial resistance genes.

**FIG 6:**
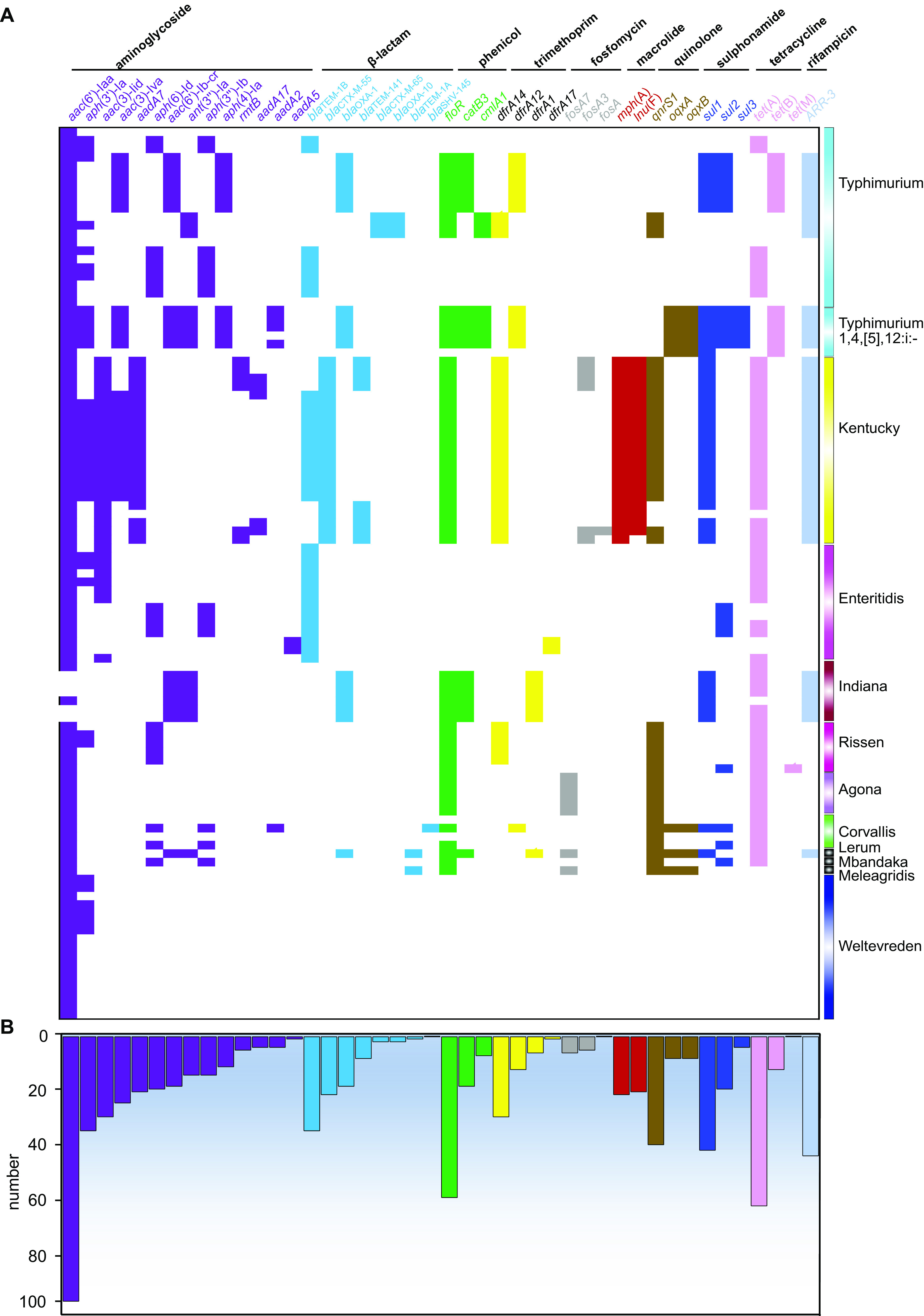
Heat map showing the AMR gene profiles in this work. Different groups of AMR genes are color-coded. (A) Distribution of AMR genes among different serotypes of Salmonella. (B) Numbers of AMR genes in the 105 Salmonella isolates.

The screening for the AMR determinants by using Abricate with the ARG database showed that 95.23% (100/105) of the isolates harbored the *aac(6′)-Iaa* gene (Fig. 6). Also, the results showed that 61 (58.08%) isolates harbored *tet*(A), encoding resistance to tetracyclines, while 60 (57.18%) isolates harbored the *floR* gene, suggesting resistance to phenicols. Notably, around 40% of the isolates harbored *qnrS1*, *sul1*, and *arr3*, encoding resistance to quinolones, sulfonamide, and rifampin, respectively (Fig. 6; see also Fig. S4). Notably, genes encoding β-lactamases, such as *bla*_TEM-1_ and *bla*_OXA-1_, were identified in a range of different serotypes, including 37% (10/27) *S.* Typhimurium and a monophasic variant, 100% (14/14) S. Enteritidis, 100% (22/22) *S.* Kentucky, 24.8% (3/7) *S.* Indiana, and 1 *S.* Lerum isolate. At the farm level, we also noticed that a higher prevalence of AMR genes was identified in one chicken farm (F17) and one pig farm (F19), which was in good agreement with the AMR phenotype (see Fig. S4). We noticed that there was a strong relationship between IncHI2 and IncHI2A and *dfrA12*, *catB3*, *bla*_OXA-1_, *acc(6’)-Ib-cr*, and *ant(3”)-la* genes, as both plasmids and resistance genes have only been detected in *S.* Typhimurium, *S.* Lerum, and *S.* Indiana isolates. Moreover, phenotypic and genotypic AMR profiles were highly correlated in this work. The consistency of acquired AMR genes and resistance phenotypes to ampicillin, ceftiofur, ceftazidime, meropenem, sulfamethoxazole-trimethoprim, and tetracycline was >90%. The above data on the two quinolones, enrofloxacin and ofloxacin, showed <70% consistency (see Table S5).

### Virulence genes.

**FIG 7:**
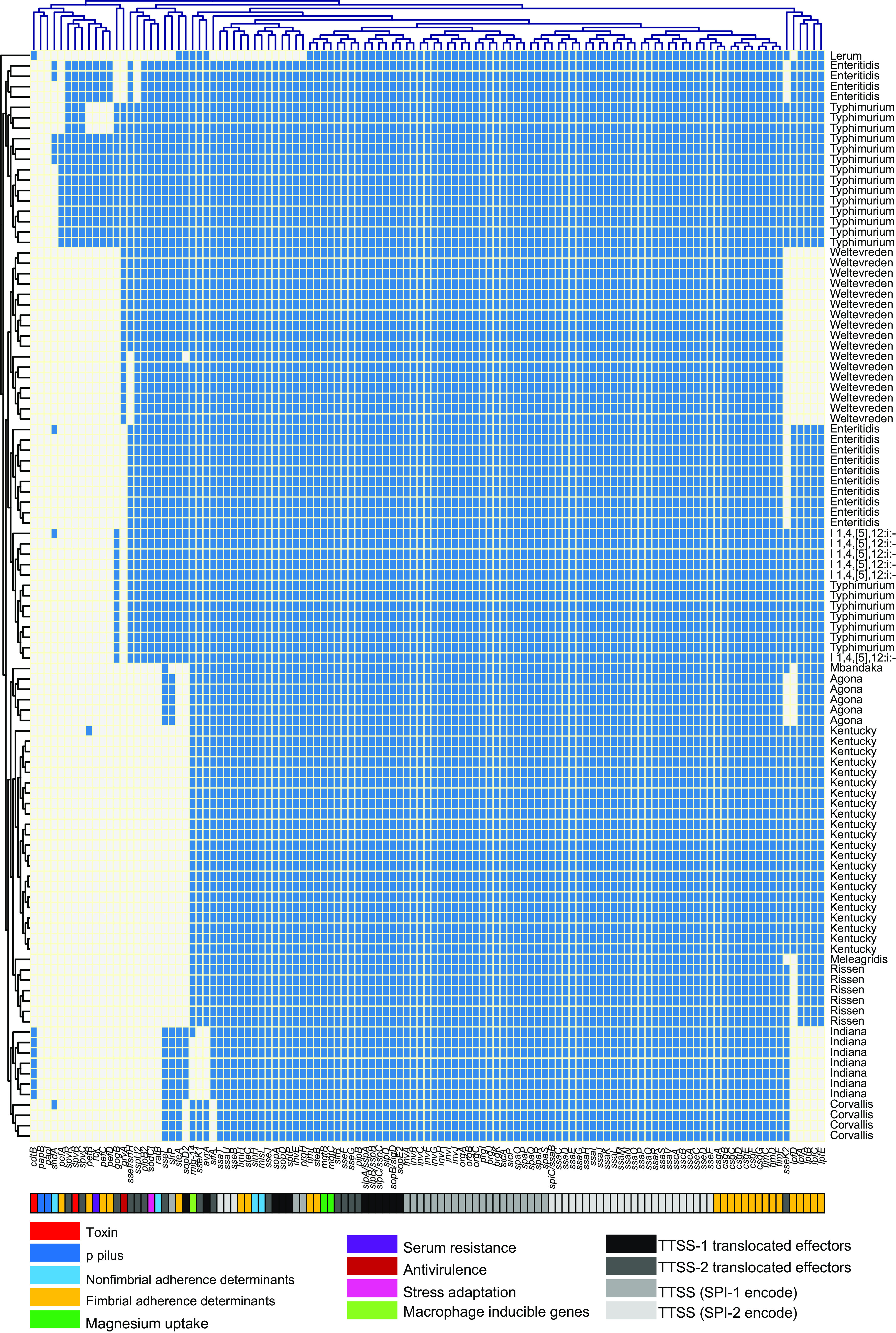
Dendrogram of a hierarchical clustering heat map of the isolates and virulence genes. The figure shows the prediction of virulence gene factor profiles for the studied isolates. The *y* axis shows the isolate ID numbers, and the *x* axis shows the identified selected virulence genes. Blue cells, presence of gene; gray cells, absence of gene.

In total, 115 virulence genes implicated in different virulence pathogenicity mechanisms were identified from the WGS data (see Table S4). Our results showed that the virulence gene distribution strongly correlated with serotypes (Fig. 7). *S.* Typhimurium harbored a higher number of virulence genes than the other serotypes. Fimbrial adherence factor (*pef)* genes and the nonfimbrial adherence factor (*shdA*) gene were detected only in *S.* Typhimurium isolates. Additionally, the serum resistance gene *rck*, stress adaptation gene *sodCl*, and plasmid-borne *spv* genes (*spvB*, *spvC*, and *spvR*), which play an essential role in the virulence systems of nontyphoid Salmonella strains, were also detected in 14 *S.* Typhimurium and 4 S. Enteritidis isolates. Importantly, these critical genes were found in the isolates that originated from different hosts in both provinces (Fig. 7). Our results also showed that seven isolates of *S.* Indiana carried the gene *cdtB* encoding typhoid toxin. All the examined isolates harbored the typical virulence factors from Salmonella pathogenicity islands 1 and 2 (SPI-1 and SPI-2) (Fig. 7; see also Table S3).

## DISCUSSION

Salmonella is a major foodborne pathogen worldwide and presents severe food safety risks. Salmonella has been recognized as one of the most widespread pathogenic foodborne bacteria in Chinese food products (3). Epidemiological monitoring and hygienic control of Salmonella through the food chain are significant priorities. Traditionally, conventional bacterial characterization and genotyping are time-consuming and deliver a portion of results with incomparability across different investigations (22, 23). The WGS analytic framework proposed in this study has been demonstrated to be an efficient and cost-effective approach for investigating food safety (18, 20, 24).

In this study, the cumulative prevalence of Salmonella in samples collected from pigs, chickens, and ducks in Fujian and Zhejiang Provinces in the eastern part of China was ≈7%. However, Salmonella isolates were more prevalent in duck farms, followed by chicken and pig farms. The total cumulative prevalence was 7.1% (105/1,480). These results were in agreement with those reported by Ibrahim et al. (25), who reported a prevalence of 6.5% (25/384) of Salmonella isolates in broiler farms in Malaysia. However, it was lower than the cumulative prevalence reported in pigs, ducks, and chicken farms in Sichuan Province, China (165/1382, 11.9%) (26). Additionally, it was higher than data reported in northwestern Spanish broiler houses (67/6,577, 1.0%) (27). Moreover, our results showed that the prevalence of Salmonella isolates in chicken and duck samples collected from farms (6.81%, 60/880) was approximately similar to those collected from live animal markets (6.33%; 38/600), which raises a significant concern. Notably, the presence of Salmonella in asymptomatic animal carriers poses a severe public health concern (24, 28). Many strategies should be properly conducted to avoid the transmission of Salmonella to humans through the food chain, including the strict application of critical hygienic practices on farms and live animal markets (29, 30).

Serotype prediction suggested a wide range of Salmonella serotypes circulating in animal farms and markets. Serovar prediction showed the dominance of *S.* Kentucky and *S.* Typhimurium among the studied Salmonella isolates. These serovars were detected throughout the samples collected from both provinces, including farms and live animal markets. *S.* Kentucky has been previously reported as the dominant serovar in chicken slaughterhouses in Jiangsu Province (31) and chicken broilers in Sichuan Province (32) in China. In contrast, several previous works reported *S.* Typhimurium as a dominant serovar in chicken (33, 34), ducks (34), and pigs (35) in China. Another recent study also reported a higher prevalence of Salmonella in retail duck meat (36) and in pork and chicken meat (37) in southern China. These reports provided strong evidence of the increasing prevalence of *S.* Kentucky and *S.* Typhimurium in the poultry and pig farms as well as their supply chain in China. However, several studies have also reported that *S.* Kentucky and *S.* Typhimurium are the dominant serotypes isolated from chickens, pigs, and ducks worldwide (38–41). Indeed, the nonadherence to hygienic practices during slaughtering could play an essential role in increasing the contamination of animal carcasses by pathogenic bacteria after their contact with intestinal contents in slaughter facilities (28).

Our results also suggested that 75.26% of the examined Salmonella isolates were MDR that could resist at least three antimicrobial agent classes. The surge in antimicrobial-resistant Salmonella isolates is recognized as a crucial public health issue (42). The majority of Salmonella isolates were resistant to tetracycline (76.6%) and ampicillin (67.2%), which are the first-line drugs used against bacterial infection in animal farms worldwide (43, 44). These results are in good agreement with Liu et al. (6), who reported a higher percentage of tetracycline (73.4%) and ampicillin (69%) resistance in Salmonella isolates obtained from farms of animal food in Xinjiang, China. The resistance to quinolones and beta-lactams was also recognized in many Salmonella isolates in this study, in agreement with other reports (2, 28, 45) that indicated high-level resistance to quinolones and beta-lactams in Salmonella isolates obtained from pork and chicken products. These findings are regarded as significant threats to public health, since these antimicrobial classes are currently considered drugs of choice for salmonellosis infection treatment in humans (46). The high AMR rate of *S.* Kentucky and *S.* Typhimurium, with a widespread AMR spectrum, is of concern. This type of bacterial resistance has been previously detected in *S.* Kentucky isolates obtained in Xinjiang Province (6) and southern China (37). Moreover, the Salmonella isolates showed resistance to colistin, which is considered one of the last-resort therapeutic options for the treatment of multidrug-resistant *Enterobacteriaceae* (46), and this finding of colistin resistance was in agreement with results reported by other recent studies (24, 47, 48) in Salmonella isolates from poultry and live pigs.

Prediction of plasmid distributions among the studied Salmonella isolates showed that Salmonella isolates harbored several plasmids, with IncFll(S) dominance followed by IncHI2 and IncHI2A. Our results also showed that these plasmids were carried by different serotypes originating from chickens, ducks, and pigs from the two provinces, indicating that these plasmids can disseminate widely among different hosts, which is of great importance (46). Additionally, these plasmids have been linked with resistance to several antibiotic classes, including beta-lactams, aminoglycosides, sulfonamides, and tetracyclines (18, 49). Furthermore, IncHI2, IncHI2A, and IncF plasmids are often associated with persistence and harbor virulence genes that contribute to bacterial fitness (17, 19, 49–52).

Genomic analysis showed that the Salmonella isolates harbored different AMR genes, which could be correlated with the high-level AMR phenotype. The majority (95.2%) of the studied Salmonella isolates harbored the *aac(6′)-Iaa* gene. However, *aac(6′)-Iaa* and similar genes usually are transcriptionally silent and rarely become transcriptionally active. The mere presence of this gene does not confer aminoglycoside resistance in Salmonella (53, 54). Besides, 42.86% (44/105) of the isolates were resistant to the aminoglycoside class and harbored different aminoglycoside resistance genes. Among diverse mechanisms of aminoglycoside resistance, enzymatic modification is the most prevalent mechanism in pathogenic bacteria, including Salmonella spp. (55, 56). Moreover, our study revealed 71/105 (67.7%) of the isolates belonged to S. Enteritidis, *S.* Kentucky, *S.* Typhimurium, and *S.* Indiana serotypes and that were resistant to ampicillin harbored different beta-lactam resistance genes. The *bla* genes control the resistance to beta-lactam antimicrobials by hydrolyzing the beta-lactam ring, leading to antibiotic inactivation (57, 58). The *bla*_TEM-1B_ gene, which confers resistance to ampicillin, is the dominant beta-lactam in most Salmonella serotypes worldwide (58–60). Resistance to tetracyclines was detected in 80/105 (76.2%) of the isolates due to the different *tet* resistance genes in the studied isolates. Additionally, the plasmid-mediated quinolone resistance gene *qnrS1* was identified in among the examined isolates belonging to different serotypes. These critical genes in Salmonella isolates from food animals present a tremendous public health concern. It is essential that the existence of the acquired AMR genes in bacterial genomes does not inevitably confer phenotypic resistance and vice versa. Other mechanisms, such as single-nucleotide polymorphisms, in MDR transporter and AMR expression regulation also significantly contribute to the phenotypic resistance (2, 61). Hence, phenotypic confirmation of the studied isolates is still essential for validating antimicrobial-resistant profiles.

Considering the investigated virulence factors, *S.* Typhimurium isolates displayed a more comprehensive range of virulence determinants than other serotypes. Our study reported detection of the *cdtB* gene encoding typhoid toxins in seven Salmonella isolates that belonged to serotype Indiana. Fimbrial adherence *pef* genes, which were previously reported as plasmid-borne determinants and have an essential role in the adhesion of Salmonella to the host gut epithelium (62–64), were only reported in *S.* Typhimurium isolates. Additionally, the serum resistance gene *rck*, stress adaptation gene *sodCl*, and plasmid-borne *spv* genes (*spvB*, *spvC*, *and spvR*), which play an essential role in the virulence system of nontyphoid Salmonella strains, were detected in 14 *S.* Typhimurium and 4 S. Enteritidis isolates. Serum resistance gene *rck* enhances the bacterial adhesion and invasion and confers high resistance to the bactericidal activity of the complement system (65, 66). Stress adaptation gene *sodCl* is vital in protecting Salmonella from phagocytic superoxide during infection (67, 68). Moreover, all the examined isolates carried the typical virulence factors from Salmonella pathogenicity islands 1 and 2. These results are consistent with our previous studies that reported the presence of *pef*, *rck*, *spv*, *rck*, and *sodCl* genes from different Salmonella serotypes collected from pigs (19, 24) and dead chick embryos (45) obtained from Henan Province in China. In addition, Yang et al. also reported the presence of *pef* and *rck* genes from duck farms and slaughterhouses in Shandong Province in China (69).

Together, we found a considerable diversity of Salmonella serotypes circulating in the animal food farms in Zhejiang and Fujian Provinces, China, with MDR and virulence potentials. This study also demonstrated the importance of whole-genome sequencing as a cost-effective approach for epidemiological analysis of Salmonella isolates. We reported the high prevalence of MDR Salmonella in the studied samples and the presence of numerous virulence determinants in the examined isolates, posing a severe risk to food safety. Therefore, it is essential to continue monitoring the Salmonella serovars, implement the necessary prevention and strategic control plans, and conduct an epidemiological surveillance system based on whole-genome sequencing. Applying an antimicrobial management plan for rational use of essential antimicrobials in animal farms is vital to improve food safety and prevent the emergence of MDR bacteria.

## MATERIALS AND METHODS

### Sample collection.

Between June and October 2019, 1,480 samples were collected, including 880 from 22 different farms (fecal and anal swabs of chicken, ducks, and pigs) and 600 from two mixed chicken and duck markets (mainly chickens; the proportion is unknown). The samples were collected from four cities (Ningbo, Shaoxing, Jiaxing, and Jinhua) located in Zhejiang Province and five cities (Fuzhou, Nanping, Xiamen, Putian, and Zhangzhou) located in Fujian Province in the eastern part of China (Fig. 1). The detailed information on sample collection is shown in Table S1 in the supplemental material.

### Ethics statement.

All of the activities in our study were approved by the institutional review board of the Zhejiang Academy of Agricultural Sciences. Samples were collected for bacterial isolation after the farmer’s verbal consent.

### Isolation and identification of isolates.

The isolation of Salmonella was performed according to methods described previously (2, 70). Briefly, fecal or anal swab samples were preenriched in 10 mL buffered peptone water (BPW; Landbridge, Beijing, China). Following the initial preenrichment in BPW, 0.1 mL of the preenriched sample was added to 10 mL Selenite cystine broth (Landbridge, Beijing, China) and incubated at 37°C for 12 to 18 h. Final colony isolation was done on xylose-lysine-Tergitol-4 agar (BD Biosciences, USA). The positive Salmonella isolates were further identified by matrix-assisted laser desorption ionization (MALDI)–time of flight mass spectrometry (Bruker MALDI Biotyper System, Germany).

### Serotyping by agglutination assay.

The isolates were serotyped using O- and H-antigens by slide agglutination with hyperimmune sera, and the serotypes of Salmonella samples were identified following the Kauffmann-White scheme (71).

### Antimicrobial susceptibility tests.

AMR profiles of recovered Salmonella isolates were determined by the broth microdilution method (Bio Fosun, Fosun Diagnostics, Shanghai, China) (72). The antibiotic concentration range of the 14 antimicrobial agents used in this assay were as follows: ampicillin (AMP), 0.25 to 512 μg/mL; amoxicillin and clavulanate potassium (AMC), 0.25/0.125 to 512/256 μg/mL; gentamicin (GEN), 0.25 to 32 μg/mL; spectinomycin (SPT), 0.125 to 256 μg/mL; tetracycline (TET), 0.125 to 256 μg/mL, florfenicol (FFL), 0.125 to 256 μg/mL; sulfafurazole (SF), 0.125 to 256 μg/mL; trimethoprim-sulfamethoxazole (SXT), 0.06/1.2 to 32/608 μg/mL; ceftiofur (CEF), 0.03 to 64 μg/mL; ceftazidime (CAZ), 0.03 to 64 μg/mL; enrofloxacin (ENR), 0.015 to 32 μg/mL; ofloxacin (OFL), 0.03 to 64 μg/mL, colistin (CST), 0.03 to 64 μg/mL; and meropenem (MEM), 0.015 to 32 μg/mL. The breakpoints for each antimicrobial agent were those set by the Clinical and Laboratory Standards Institute (73) (see Table S6). Escherichia coli ATCC 25922 served as a control strain in all the assays.

### Whole-genome sequencing and bioinformatics analysis.

Genomic DNA extraction was performed from overnight cultures of isolates grown in Luria-Bertani broth at 37°C under shaking conditions (180 rpm) and then use of a bacterial DNA extraction kit (General Biotech, Shanghai, China) per the manufacturer’s instructions. The extracted DNA was quantified using the Qubit 2.0 fluorometer (Invitrogen, CA, USA). All Illumina sequencing libraries were generated using a NEXTflex DNA sequencing kit (Bioo Scientific, USA). All isolates were subjected to whole-genome sequencing using the HiSeq platform (Illumina, San Diego, CA, USA). The paired-end reads (2 × 150 bp) were checked for quality and trimmed with Trimmomatic v0.36. All low-quality (*Q* of <20) data were filtered out. The raw sequence reads underwent a quality check and were assembled with SPAdes v3.12.0. (74) using the “careful” command line option. The Quast tool 5.0.2 (75) was used to evaluate the quality of the assembled genomes. Gene prediction and genome annotation were performed using the NCBI Prokaryotic Genome Annotation Pipeline. After the completion of genome assembly, serotype prediction was performed with SISTR v1.0.2 (76) using default parameters. In addition, the virulence genes and AMR genes were predicted by using the Abricate 1.0.1 tool (https://github.com/tseemann/abricate), which combines data sets from ResFinder 4.1 (https://cge.cbs.dtu.dk/services/ResFinder/; accessed 7 October 2021), with a similarity cutoff of 90% nucleotide identity and 90% minimum coverage (77), and the VFDB database (accessed 7 October 2021) with 70% minimum coverage and 50% nucleotide identity (78). In addition, plasmid types were detected using PlasmidFinder 2.1 (https://cge.cbs.dtu.dk/services/PlasmidFinder/), with a cutoff of 90% nucleotide identity and 60% minimum coverage. Furthermore, MLST was conducted at the Center for Genomic Epidemiology (https://cge.cbs.dtu.dk/services/MLST/). The minimum spanning tree was generated using GrapeTree. The above bioinformatics analysis pipeline is shown in the schematic diagram in Fig. S5.

### Statistical analysis.

The chi-square test was used to analyze the prevalence differences from different sources. The prevalence of various farms was grouped for the *t* test. The heatmaps of clustering of sources, virulence genes, plasmid replicons, AMR genes, serotypes, and AMR phenotypes were created by using TBtools (79). In this analysis, the presence of the indicated gene or phenotype received a score of 1 and its absence received a score of 0.

### Data availability.

The genomic data produced in this study are available under BioProject accession PRJNA751163.
