# Supplementary material for: Genomic Characterization of Antimicrobial-Resistant Salmonella enterica in Duck, Chicken, and Pig Farms and Retail Markets in Eastern China
Source: Microbiol Spectr. 2022 Sep 1;10(5):e01257-22. doi: 10.1128/spectrum.01257-22 (PMC9603869; doi:10.1128/spectrum.01257-22)
Supplement: Supplemental file 1 — Fig. S1 to S5 and Tables S1 to S6. Download spectrum.01257-22-s0001.pdf, PDF file, 2.8 MB [file spectrum.01257-22-s0001.pdf]

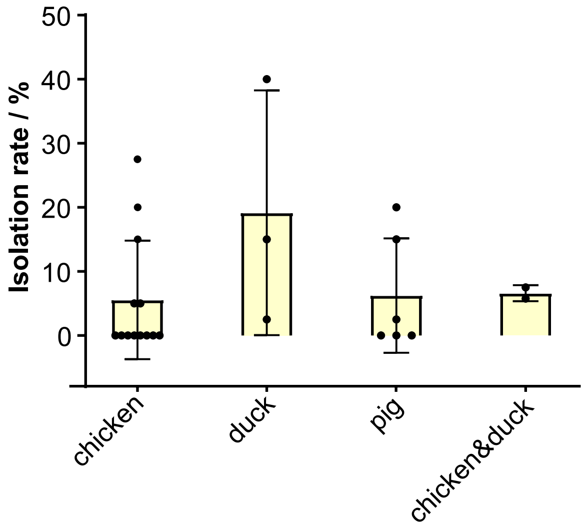

Figure S1. The isolation rate of *Salmonella* in 24 sampling areas from different sources.

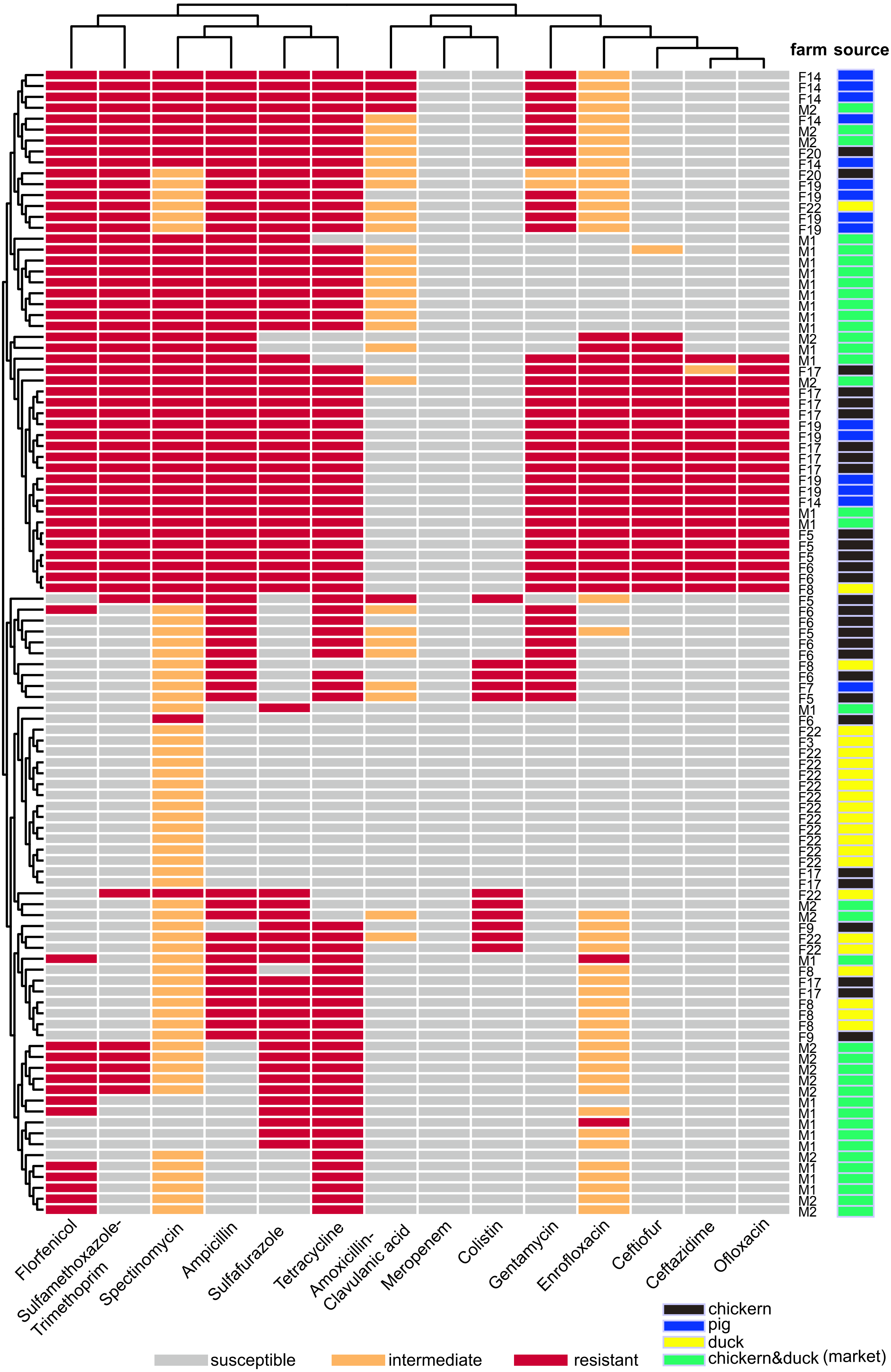

Figure S2. Sankey diagram combining the provinces, serotypes, sequence types and sampling sources based on 105 *Salmonella* isolates.

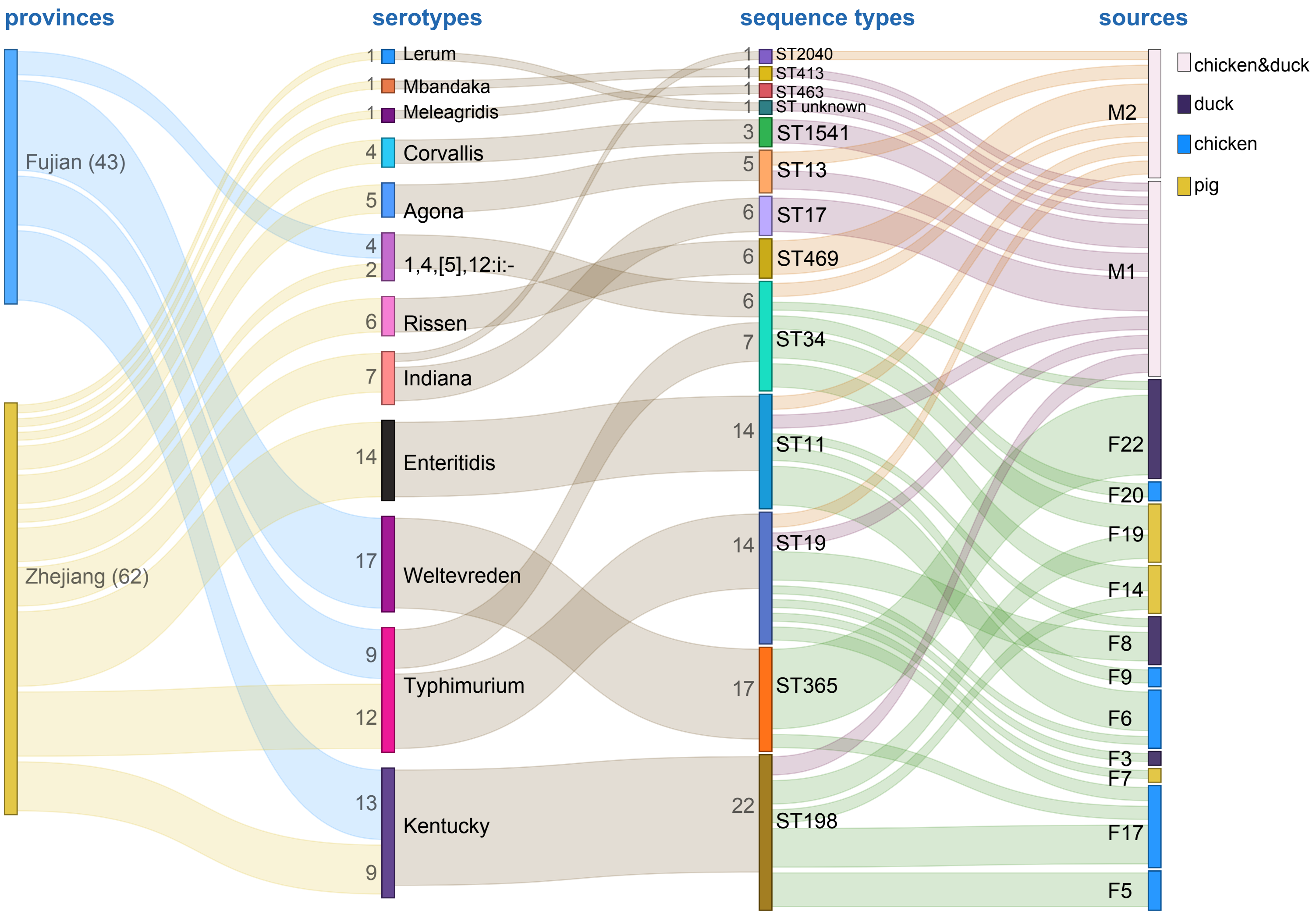

Figure S3. Dendrogram of hierarchical clustering heatmap of the AMR phenotypes and farms. Y-axis refers to the farms, and X-axis refers to the AMR phenotypes.

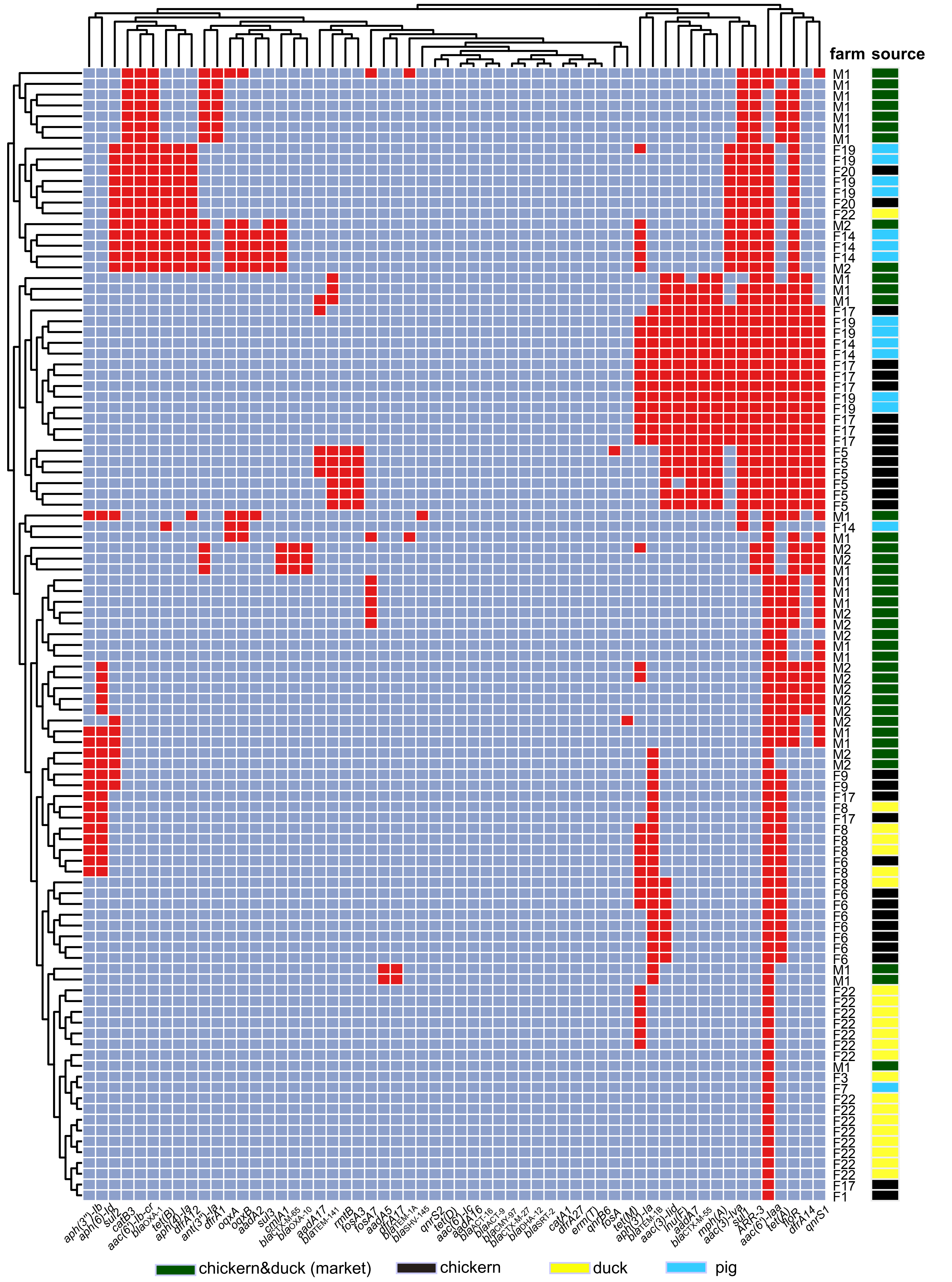

Figure S4. Dendrogram of hierarchical clustering heatmap of the AMR genes and farms. Y-axis refers to the farms and sources. The X-axis refers to the identified AMR genes. The red color in the cell relates to the existence, and the baby blue color relates to absence.

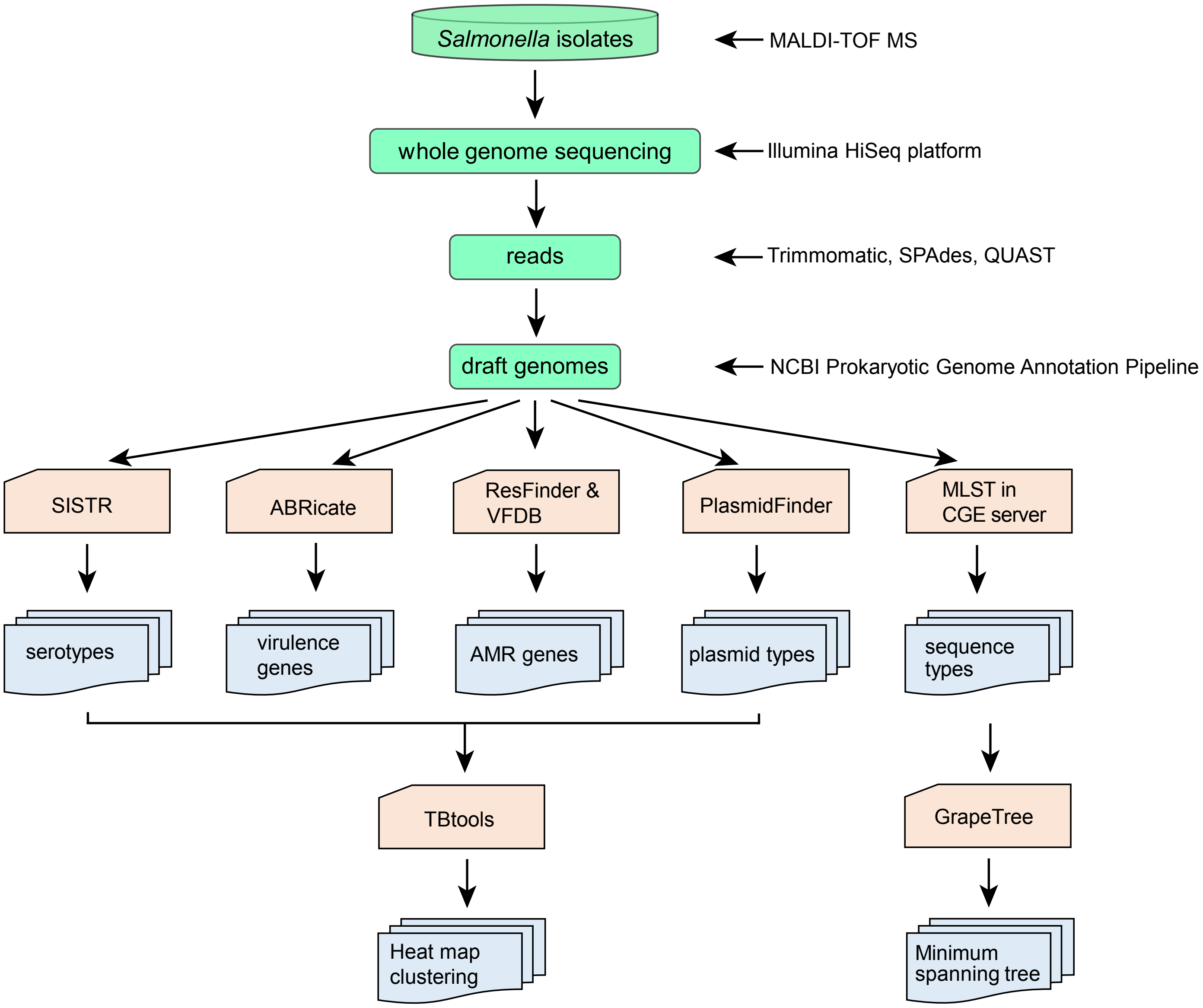

Figure S5. Bioinformatics analysis pipeline in this study.

Table S1. Sampling and isolation of *Salmonella* in this study

Province	City	Farm	Animal	Sample	*Salmonella*	Isolation%
Zhejiang	Ningbo	F1	chicken	40	0	0
		M1	chicken&duck	400	23	5.75
	Shaoxing	M2	chicken&duck	200	15	7.5
		F2	chicken	40	0	0
		F3	duck	40	1	2.5
	Jiangxing	F4	chicken	40	0	0
		F5	chicken	40	6	15
		F6	chicken	40	8	20
		F7	pig	40	1	2.5
	Jinhua	F8	duck	40	6	15
		F9	chicken	40	2	5
		F10	pig	40	0	0
**Sum:**				**1000**	**62**	**6.2**
Fujian	Fuzhou	F11	chicken	40	0	0
		F12	chicken	40	0	0
	Nanping	F13	chicken	40	0	0
	Xiamen	F14	pig	40	6	15
		F15	pig	40	0	0
		F16	pig	40	0	0
	Putian	F17	chicken	40	11	27.5
		F18	chicken	40	0	0
		F19	chicken	40	8	20
	Zhangzhou	F20	chicken	40	2	5
		F21	chicken	40	0	0
		F22	duck	40	16	40
**Sum:**				**480**	**43**	**8.959**

Table S2. Statistics of the prevalence between different sampling groups.

groups	*Salmonella* (+)	*Salmonella* (-)	samples	proportion%	X^2	*P*
Zhejiang	**62**	**938**	1000	6.20	3.743729	0.05301
Fujian	**43**	**437**	480	8.96		
Sum:	105	1375	1480			
Chicken	**37**	**523**	560	6.61	19.37678	0.00002
Duck	**23**	**97**	120	19.17		
Sum:	60	620	680			
Duck	**23**	**97**	120	19.17	21.66682	0.00001
Pig	**7**	**193**	200	3.50		
Sum:	30	290	320			
Chicken	**37**	**523**	560	6.61	2.608485	0.1063
Pig	**7**	**193**	200	3.50		
Sum:	44	716	760			
live animal markets	**38**	**562**	600	6.34	2.79572	0.09452
Chicken and duck farms	**60**	**620**	680	8.83		
Sum:	98	1182	1280			

Table S3. The prevalence, antimicrobial resistance, and genomic characterization of 105 *Salmonella* isolates.

abbreviation	strain	in silico Serotype	Species	Provience	location	City	Animal	ST	*aroC*	*dnaN*	*hemD*
**abbreviation**	**strain**	**in silico Serotype**	**Species**	**Provience**	**location**	**City**	**Animal**	**ST**	***aroC***	***dnaN***	***hemD***
z01	SC-Z1910-0001	Typhimurium	*Salmonella*	Zhejiang	天天田园 [M2]	Shaoxing	Chicken&Duck	19	10	7	12
z02	SC-Z1910-0002	Rissen	*Salmonella*	Zhejiang	天天田园 [M2]	Shaoxing	Chicken&Duck	469	92	107	79
z03	SC-Z1910-0003	Rissen	*Salmonella*	Zhejiang	天天田园 [M2]	Shaoxing	Chicken&Duck	469	92	107	79
z04	SC-Z1910-0004	Rissen	*Salmonella*	Zhejiang	天天田园 [M2]	Shaoxing	Chicken&Duck	469	92	107	79
z05	SC-Z1910-0005	Enteritidis	*Salmonella*	Zhejiang	天天田园 [M2]	Shaoxing	Chicken&Duck	11	5	2	3
z06	SC-Z1910-0006	Typhimurium	*Salmonella*	Zhejiang	天天田园 [M2]	Shaoxing	Chicken&Duck	19	10	7	12
z07	SC-Z1910-0007	Rissen	*Salmonella*	Zhejiang	天天田园 [M2]	Shaoxing	Chicken&Duck	469	92	107	79
z08	SC-Z1910-0008	Enteritidis	*Salmonella*	Zhejiang	天天田园 [M2]	Shaoxing	Chicken&Duck	11	5	2	3
z09	SC-Z1910-0009	Indiana	*Salmonella*	Zhejiang	天天田园 [M2]	Shaoxing	Chicken&Duck	2040	302	8	11
z10	SC-Z1910-0010	Rissen	*Salmonella*	Zhejiang	天天田园肉 [M2]	Shaoxing	Chicken&Duck	469	92	107	79
z11	SD-Z1910-0011	Typhimurium	*Salmonella*	Zhejiang	国伟禽业 [F3]	Shaoxing	Duck	19	10	7	12
z12	SC-Z1910-0012	Kentucky	*Salmonella*	Zhejiang	水乡情农场 [F5]	Jiaxing	Chicken	198	76	14	3
z13	SC-Z1910-0013	Kentucky	*Salmonella*	Zhejiang	水乡情农场 [F5]	Jiaxing	Chicken	198	76	14	3
z14	SC-Z1910-0014	Kentucky	*Salmonella*	Zhejiang	水乡情农场 [F5]	Jiaxing	Chicken	198	76	14	3
z15	SC-Z1910-0015	Kentucky	*Salmonella*	Zhejiang	水乡情农场 [F5]	Jiaxing	Chicken	198	76	14	3
z16	SP-Z1910-0016	Typhimurium	*Salmonella*	Zhejiang	嘉华 [F7]	Jiaxing	Pig	19	10	7	12
z17	SC-Z1910-0017	Enteritidis	*Salmonella*	Zhejiang	马库 [F6]	Jiaxing	Chicken	11	5	2	3
z18	SC-Z1910-0018	Enteritidis	*Salmonella*	Zhejiang	马库 [F6]	Jiaxing	Chicken	11	5	2	3
z19	SC-Z1910-0019	Typhimurium	*Salmonella*	Zhejiang	马库 [F6]	Jiaxing	Chicken	19	10	7	12
z20	SC-Z1910-0020	Kentucky	*Salmonella*	Zhejiang	水乡情 [F5]	Jiaxing	Chicken	198	76	14	3
z21	SC-Z1910-0021	Enteritidis	*Salmonella*	Zhejiang	马库 [F6]	Jiaxing	Chicken	11	5	2	3
z22	SC-Z1910-0022	Enteritidis	*Salmonella*	Zhejiang	马库 [F6]	Jiaxing	Chicken	11	5	2	3
z23	SC-Z1910-0023	Enteritidis	*Salmonella*	Zhejiang	马库 [F6]	Jiaxing	Chicken	11	5	2	3
z24	SC-Z1910-0024	Enteritidis	*Salmonella*	Zhejiang	马库 [F6]	Jiaxing	Chicken	11	5	2	3
z25	SC-Z1910-0025	Enteritidis	*Salmonella*	Zhejiang	马库 [F6]	Jiaxing	Chicken	11	5	2	3
z26	SC-Z1910-0026	Kentucky	*Salmonella*	Zhejiang	水乡情 [F5]	Jiaxing	Chicken	198	76	14	3
z27	SD-Z1910-0027	Typhimurium	*Salmonella*	Zhejiang	金婺 [F8]	Jinhua	Duck	19	10	7	12
z28	SD-Z1910-0028	Enteritidis	*Salmonella*	Zhejiang	金婺 [F8]	Jinhua	Duck	11	5	2	3
z29	SD-Z1910-0029	Typhimurium	*Salmonella*	Zhejiang	金婺 [F8]	Jinhua	Duck	19	10	7	12
z30	SD-Z1910-0030	Typhimurium	*Salmonella*	Zhejiang	金婺 [F8]	Jinhua	Duck	19	10	7	12
z31	SD-Z1910-0031	Typhimurium	*Salmonella*	Zhejiang	金婺 [F8]	Jinhua	Duck	19	10	7	12
z32	SD-Z1910-0032	Typhimurium	*Salmonella*	Zhejiang	金婺 [F8]	Jinhua	Duck	19	10	7	12
z33	SC-Z1910-0033	Enteritidis	*Salmonella*	Zhejiang	绿发农业 [F9]	Jinhua	Chicken	11	5	2	3
z34	SC-Z1910-0034	Enteritidis	*Salmonella*	Zhejiang	绿发农业 [F9]	Jinhua	Chicken	11	5	2	3
z35	SD-F1910-0035	Weltevreden	*Salmonella*	Fujian	比格山 [F22]	hangzho	Duck	365	130	97	25
z36	SD-F1910-0036	Weltevreden	*Salmonella*	Fujian	比格山 [F22]	hangzho	Duck	365	130	97	25
z37	SD-F1910-0037	Weltevreden	*Salmonella*	Fujian	比格山 [F22]	hangzho	Duck	365	130	97	25
z38	SD-F1910-0038	Weltevreden	*Salmonella*	Fujian	比格山 [F22]	hangzho	Duck	365	130	97	25

z39	SD-F1910-0039	Weltevreden	*Salmonella*	Fujian	比格山 [F22]	hangzhc	Duck	365	130	97	25
z40	SD-F1910-0040	Weltevreden	*Salmonella*	Fujian	比格山 [F22]	hangzhc	Duck	365	130	97	25
z41	SD-F1910-0041	Weltevreden	*Salmonella*	Fujian	比格山 [F22]	hangzhc	Duck	365	130	97	25
z42	SD-F1910-0042	Weltevreden	*Salmonella*	Fujian	比格山 [F22]	hangzhc	Duck	365	130	97	25
z43	SD-F1910-0043	Weltevreden	*Salmonella*	Fujian	比格山 [F22]	hangzhc	Duck	365	130	97	25
z44	SD-F1910-0044	Weltevreden	*Salmonella*	Fujian	比格山 [F22]	hangzhc	Duck	365	130	97	25
z45	SD-F1910-0045	Weltevreden	*Salmonella*	Fujian	比格山 [F22]	hangzhc	Duck	365	130	97	25
z46	SD-F1910-0046	Weltevreden	*Salmonella*	Fujian	比格山 [F22]	hangzhc	Duck	365	130	97	25
z47	SD-F1910-0047	Weltevreden	*Salmonella*	Fujian	比格山 [F22]	hangzhc	Duck	365	130	97	25
z48	SD-F1910-0048	Weltevreden	*Salmonella*	Fujian	比格山 [F22]	hangzhc	Duck	365	130	97	25
z49	SC-F1910-0049	Weltevreden	*Salmonella*	Fujian	温氏1号厂 [F17]	Putian	Chicken	365	130	97	25
z50	SC-F1910-0050	Weltevreden	*Salmonella*	Fujian	温氏1号厂 [F17]	Putian	Chicken	365	130	97	25
z51	SC-F1910-0051	Kentucky	*Salmonella*	Fujian	温氏1号厂 [F17]	Putian	Chicken	198	76	14	3
z52	SC-F1910-0052	Kentucky	*Salmonella*	Fujian	温氏1号厂 [F17]	Putian	Chicken	198	76	14	3
z53	SC-F1910-0053	Kentucky	*Salmonella*	Fujian	温氏1号厂 [F17]	Putian	Chicken	198	76	14	3
z54	SC-F1910-0054	Kentucky	*Salmonella*	Fujian	温氏1号厂 [F17]	Putian	Chicken	198	76	14	3
z55	SC-F1910-0055	Kentucky	*Salmonella*	Fujian	木兰屠宰场 [F19]	Putian	Chicken	198	76	14	3
z56	SC-F1910-0056	Kentucky	*Salmonella*	Fujian	木兰屠宰场 [F19]	Putian	Chicken	198	76	14	3
z57	SC-F1910-0057	Typhimurium	*Salmonella*	Fujian	木兰屠宰场 [F19]	Putian	Chicken	34	10	19	12
z58	SC-F1910-0058	Typhimurium	*Salmonella*	Fujian	木兰屠宰场 [F19]	Putian	Chicken	34	10	19	12
z59	SC-F1910-0059	Typhimurium	*Salmonella*	Fujian	木兰屠宰场 [F19]	Putian	Chicken	34	10	19	12
z60	SC-F1910-0060	Typhimurium	*Salmonella*	Fujian	碧山 [F20]	hangzhc	Chicken	34	10	19	12
z61	SD-F1910-0061	Typhimurium	*Salmonella*	Fujian	比格山 [F22]	hangzhc	Duck	34	10	19	12
z62	SD-F1910-0062	Weltevreden	*Salmonella*	Fujian	比格山 [F22]	hangzhc	Duck	365	130	97	25
z63	SC-F1910-0063	Typhimurium	*Salmonella*	Fujian	温氏1号厂 [F17]	Putian	Chicken	19	10	7	12
z64	SC-F1910-0064	Typhimurium	*Salmonella*	Fujian	温氏1号厂 [F17]	Putian	Chicken	19	10	7	12
z65	SC-F1910-0065	Kentucky	*Salmonella*	Fujian	温氏1号厂 [F17]	Putian	Chicken	198	76	14	3
z66	SC-F1910-0066	Kentucky	*Salmonella*	Fujian	温氏1号厂 [F17]	Putian	Chicken	198	76	14	3
z67	SC-F1910-0067	Kentucky	*Salmonella*	Fujian	温氏1号厂 [F17]	Putian	Chicken	198	76	14	3
z68	SC-F1910-0068	Kentucky	*Salmonella*	Fujian	木兰屠宰场 [F19]	Putian	Chicken	198	76	14	3
z69	SC-F1910-0069	Kentucky	*Salmonella*	Fujian	木兰屠宰场 [F19]	Putian	Chicken	198	76	14	3
z70	SC-F1910-0070	Typhimurium	*Salmonella*	Fujian	木兰屠宰场 [F19]	Putian	Chicken	34	10	19	12
z71	SC-F1910-0071	Typhimurium	*Salmonella*	Fujian	碧山 [F20]	hangzhc	Chicken	34	10	19	12
z72	SP-F1910-0072	Kentucky	*Salmonella*	Fujian	品加工（屠宰场）	Xiamen	Pig	198	76	14	3
z73	SP-F1910-0073	Kentucky	*Salmonella*	Fujian	品加工（屠宰场）	Xiamen	Pig	198	76	14	3
z75	SP-F1910-0075	1,4,[5],12:i:-	*Salmonella*	Fujian	品加工（屠宰场）	Xiamen	Pig	34	10	19	12
z76	SP-F1910-0076	1,4,[5],12:i:-	*Salmonella*	Fujian	品加工（屠宰场）	Xiamen	Pig	34	10	19	12
z77	SP-F1910-0077	1,4,[5],12:i:-	*Salmonella*	Fujian	品加工（屠宰场）	Xiamen	Pig	34	10	19	12
z78	SP-F1910-0078	1,4,[5],12:i:-	*Salmonella*	Fujian	品加工（屠宰场）	Xiamen	Pig	34	10	19	12
z79	SD-Z1910-0079	1,4,[5],12:i:-	*Salmonella*	Zhejiang	天天田园 [M2]	Shaoxing	Chicken&Duck	34	10	19	12
z80	SD-Z1910-0080	1,4,[5],12:i:-	*Salmonella*	Zhejiang	天天田园 [M2]	Shaoxing	Chicken&Duck	34	10	19	12
z81	SD-Z1910-0081	Rissen	*Salmonella*	Zhejiang	天天田园 [M2]	Shaoxing	Chicken&Duck	469	92	107	79
z83	SD-Z1910-0083	Agona	*Salmonella*	Zhejiang	天天田园 [M2]	Shaoxing	Chicken&Duck	13	3	3	7

z84	SD-Z1910-0084	Agona	*Salmonella*	Zhejiang	天天田园 [M2]	Shaoxing	Chicken&Duck	13	3	3	7
z85	SC-Z1910-0085	Agona	*Salmonella*	Zhejiang	宁波余姚 [M1]	Ningbo	Chicken&Duck	13	3	3	7
z86	SC-Z1910-0086	Agona	*Salmonella*	Zhejiang	宁波余姚 [M1]	Ningbo	Chicken&Duck	13	3	3	7
z87	SC-Z1910-0087	Agona	*Salmonella*	Zhejiang	宁波余姚 [M1]	Ningbo	Chicken&Duck	13	3	3	7
z88	SC-Z1910-0088	Indiana	*Salmonella*	Zhejiang	宁波余姚 [M1]	Ningbo	Chicken&Duck	17	8	8	11
z89	SC-Z1910-0089	Indiana	*Salmonella*	Zhejiang	宁波余姚 [M1]	Ningbo	Chicken&Duck	17	8	8	11
z90	SC-Z1910-0090	Meleagridis	*Salmonella*	Zhejiang	宁波余姚 [M1]	Ningbo	Chicken&Duck	463	92	125	78
z91	SD-Z1910-0091	Huddinge|Lerum	*Salmonella*	Zhejiang	宁波余姚 [M1]	Ningbo	Chicken&Duck	unknown	8	-795	11
z92	SD-Z1910-0092	Enteritidis	*Salmonella*	Zhejiang	宁波余姚 [M1]	Ningbo	Chicken&Duck	11	5	2	3
z93	SD-Z1910-0093	Enteritidis	*Salmonella*	Zhejiang	宁波余姚 [M1]	Ningbo	Chicken&Duck	11	5	2	3
z94	SD-Z1910-0094	Indiana	*Salmonella*	Zhejiang	宁波余姚 [M1]	Ningbo	Chicken&Duck	17	8	8	11
z95	SD-Z1910-0095	Typhimurium	*Salmonella*	Zhejiang	宁波余姚 [M1]	Ningbo	Chicken&Duck	19	10	7	12
z96	SD-Z1910-0096	Indiana	*Salmonella*	Zhejiang	宁波余姚 [M1]	Ningbo	Chicken&Duck	17	8	8	11
z97	SC-Z1910-0097	Indiana	*Salmonella*	Zhejiang	宁波余姚 [M1]	Ningbo	Chicken&Duck	17	8	8	11
z98	SC-Z1910-0098	Typhimurium	*Salmonella*	Zhejiang	宁波余姚 [M1]	Ningbo	Chicken&Duck	19	10	7	12
z99	SC-Z1910-0099	Indiana	*Salmonella*	Zhejiang	宁波余姚 [M1]	Ningbo	Chicken&Duck	17	8	8	11
z100	SC-Z1910-0100	Corvallis	*Salmonella*	Zhejiang	宁波余姚 [M1]	Ningbo	Chicken&Duck	1541	197	187	10
z101	SC-Z1910-0101	Kentucky	*Salmonella*	Zhejiang	宁波余姚 [M1]	Ningbo	Chicken&Duck	198	76	14	3
z102	SC-Z1910-0102	Mbandaka	*Salmonella*	Zhejiang	宁波余姚 [M1]	Ningbo	Chicken&Duck	413	15	70	93
z103	SC-Z1910-0103	Corvallis	*Salmonella*	Zhejiang	宁波余姚 [M1]	Ningbo	Chicken&Duck	1541	197	187	10
z104	SC-Z1910-0104	Corvallis	*Salmonella*	Zhejiang	宁波余姚 [M1]	Ningbo	Chicken&Duck	1541	197	187	10
z105	SC-Z1910-0105	Kentucky	*Salmonella*	Zhejiang	宁波余姚 [M1]	Ningbo	Chicken&Duck	198	76	14	3
z106	SC-Z1910-0106	Kentucky	*Salmonella*	Zhejiang	宁波余姚 [M1]	Ningbo	Chicken&Duck	198	76	14	3
z107	SC-Z1910-0107	Corvallis	*Salmonella*	Zhejiang	宁波余姚 [M1]	Ningbo	Chicken&Duck	1541	197	187	10

hisD	*purE*	*sucA*	*thrA*	O	H1	H2	Col156	ColpVC	IncFIA(HI1	IncFIB(K)	IncFIB(S)	IncFIB(pC1	IncFIB(pH(
							**plasmid**						
hisD	***purE***	***sucA***	***thrA***	**O**	**H1**	**H2**	**Col156**	**ColpVC**	**IncFIA(HI1**	**IncFIB(K)**	**IncFIB(S)**	**IncFIB(pC1**	**IncFIB(pH(**
9	5	9	2	4,5	i	2	0	0	0	0	1	0	0
156	64	151	87	6,7	f		0	0	0	0	0	0	0
156	64	151	87	6,7	f		0	0	0	0	0	0	0
156	64	151	87	6,7	f		0	0	0	0	0	0	0
7	6	6	11	9	m		0	0	0	0	1	0	0
9	5	9	2	4,5	i	2	0	0	0	0	1	0	0
156	64	151	87	6,7	f		0	0	0	0	0	0	0
7	6	6	11	9	m		0	0	0	0	1	0	0
11	5	11	15	4,12	i	1,6	0	0	0	0	0	0	0
156	64	151	87	6,7	f		0	0	0	0	0	0	0
9	5	9	2	4	i	2	0	0	0	0	1	0	0
77	64	64	67	8,20	i	z6	1	0	0	0	1	0	0
77	64	64	67	8,20	i	z6	1	0	0	0	0	0	0
77	64	64	67	8,20	i	z6	1	0	0	0	0	0	0
77	64	64	67	8,20	i	z6	1	0	0	0	0	0	0
9	5	9	2	4,5	i	2	0	0	0	0	1	0	0
7	6	6	11	9	m		0	1	0	0	0	0	0
7	6	6	11	9	m		0	1	0	0	0	0	0
9	5	9	2	4,5	i	2	0	0	0	0	1	0	0
77	64	64	67	8,20	i	z6	1	0	0	0	0	0	0
7	6	6	11	9,12	g,m		0	1	0	0	0	0	0
7	6	6	11	9,12	g,m		0	1	0	0	0	0	0
7	6	6	11	9,12	g,m		0	1	0	0	0	0	0
7	6	6	11	9,12	g,m		0	1	0	0	0	0	0
7	6	6	11	9,12	g,m		0	1	0	0	0	0	0
77	64	64	67	8,20	i	z6	1	0	0	0	0	0	0
9	5	9	2	4,5	i	2	0	0	0	0	1	0	0
7	6	6	11	9,12	g,m		0	1	0	0	0	0	0
9	5	9	2	4,5	i	2	0	0	0	0	1	0	0
9	5	9	2	4,5	i	2	0	0	0	0	1	0	0
9	5	9	2	4,5	i	2	0	0	0	0	1	0	0
9	5	9	2	4,5	i	2	0	0	0	0	1	0	0
7	6	6	11	9,12	g,m		0	0	0	0	1	0	0
7	6	6	11	9,12	g,m		0	0	0	0	1	0	0
125	84	9	101	3,10,15	r	z6	0	0	0	0	0	0	0
125	84	9	101	3,10,15	r	z6	0	0	0	0	0	0	0
125	84	9	101	3,10,15	r	z6	0	0	0	0	0	0	0
125	84	9	101	3,10,15	r	z6	0	0	0	0	0	0	0

125	84	9	101	3,10,15	r	z6	0	0	0	0	0	0	0
125	84	9	101	3,10,15	r	z6	0	0	0	0	0	0	0
125	84	9	101	3,10,15	r	z6	0	0	0	0	0	0	0
125	84	9	101	3,10,15	r	z6	0	0	0	0	0	0	0
125	84	9	101	3,10,15	r	z6	0	0	0	0	0	0	0
125	84	9	101	3,10,15	r	z6	0	0	0	0	0	0	0
125	84	9	101	3,10,15	r	z6	0	0	0	0	0	0	0
125	84	9	101	3,10,15	r	z6	0	0	0	0	0	0	0
125	84	9	101	3,10,15	r	z6	0	0	0	0	0	0	0
125	84	9	101	3,10,15	r	z6	0	0	0	0	0	0	0
125	84	9	101	3,10,15	r	z6	0	0	0	0	0	0	0
125	84	9	101	3,10,15	r	z6	0	0	0	0	0	0	0
77	64	64	67	8,20	i	z6	0	0	0	0	0	0	0
77	64	64	67	8,20	i	z6	0	0	0	0	0	0	0
77	64	64	67	8,20	i	z6	0	0	0	0	0	0	0
77	64	64	67	8,20	i	z6	0	0	0	0	0	0	0
77	64	64	67	8,20	i	z6	1	0	0	0	0	0	0
77	64	64	67	8,20	i	z6	0	0	0	0	0	0	0
9	5	9	2	1,4,5	i	2	0	0	0	0	0	0	0
9	5	9	2	1,4,5	i	2	0	0	0	0	0	0	0
9	5	9	2	1,4,5	i	2	0	0	0	0	0	0	0
9	5	9	2	1,4,5	i	2	0	0	0	0	0	0	0
9	5	9	2	4,5	i	2	0	0	0	0	0	0	0
125	84	9	101	3,10,15	r	z6	0	0	0	0	0	0	0
9	5	9	2	4,5	i	2	0	0	0	0	1	0	0
9	5	9	2	4,5	i	2	0	0	0	0	1	0	0
77	64	64	67	8,20	i	z6	0	0	0	0	0	0	0
77	64	64	67	8,20	i	z6	0	0	0	0	0	0	0
77	64	64	67	8,20	i	z6	0	0	0	0	0	0	0
77	64	64	67	8,20	i	z6	1	0	0	0	0	0	0
77	64	64	67	8,20	i	z6	1	0	0	0	0	0	0
9	5	9	2	4,5	i	2	0	0	0	0	0	0	0
9	5	9	2	4,5	i	2	0	0	0	0	0	0	0
77	64	64	67	8,20	i	z6	0	0	0	0	0	0	0
77	64	64	67	8,20	i	z6	0	0	0	0	0	0	0
9	5	9	2	4,5,12	i		0	0	0	0	0	0	0
9	5	9	2	4,5,12	i		0	0	0	0	0	0	0
9	5	9	2	4,5,12	i		0	0	0	0	0	0	0
9	5	9	2	4,5,12	i		0	0	0	0	0	0	0
9	5	9	2	4,5,12	f		0	0	0	0	0	0	0
9	5	9	2	4,5,12	f		0	0	0	0	0	0	0
156	64	151	87	6,7	f		0	0	0	0	0	0	0
4	3	3	7	4	f	s	0	0	1	0	0	0	0

4	3	3	7	4	f	s	0	0	1	0	0	0	0
4	3	3	7	4	f	g,s	0	0	1	0	0	0	0
4	3	3	7	4,12	f	g,s	0	0	1	0	0	0	0
4	3	3	7	4,12	f	g,s	0	0	1	0	0	0	0
11	5	11	15	4,12	z	1,7	0	0	0	0	0	0	1
11	5	11	15	4,12	z	1,7	0	0	0	0	0	0	1
128	138	9	141	3,10	e,h	l,w	0	0	1	1	0	0	0
11	5	11	-15	3,19	z	1,7	0	0	1	1	0	0	1
7	6	6	11	9,12	g,m		0	0	0	0	0	0	0
7	6	6	11	9,12	g,m		0	0	0	0	0	0	0
11	5	11	15	1,4,12	z	1,7	0	0	0	0	0	0	1
9	5	9	2	4,5	i	2	0	1	0	0	1	0	0
11	5	11	15	4,12	z	1,7	0	0	0	0	0	0	0
11	5	11	15	4,12	z	No agglutination	0	0	0	0	0	0	1
9	5	9	2	4,5,12	i	1,2	0	0	0	0	1	0	0
11	5	11	15	4,12	z	1,7	0	0	0	0	0	0	1
234	8	65	22	8,20	z4	z23	0	0	0	0	0	0	0
77	64	64	67	8,20	i	z6	0	0	0	0	0	0	0
78	113	6	68	6,7	z10	e,n,z15	0	0	0	0	0	0	0
234	8	65	22	8,20	No agglutination	No agglutination	0	0	0	1	0	0	0
234	8	65	22	8,20	z4	z23	0	0	0	0	0	0	0
77	64	64	67	8,20	i	z6	0	1	0	0	0	0	0
77	64	64	67	8,20	i	z6	0	0	0	0	0	0	0
234	8	65	22	8,20	z4	z23	0	0	0	0	0	0	0

IncFII(S)	IncFII(p96A	IncHI1A	IncHI1B(R2	IncHI2A	IncHI2	IncI1	IncL/M(pM	IncN	IncQ1	IncR	IncX1	IncX12	IncX4	IncY	p0111
**IncFII(S)**	**IncFII(p96A**	**IncHI1A**	**IncHI1B(R2**	**IncHI2A**	**IncHI2**	**IncI1**	**IncL/M(pM**	**IncN**	**IncQ1**	**IncR**	**IncX1**	**IncX1**	**IncX4**	**IncY**	**p0111**
1	0	0	0	0	0	0	0	0	0	0	0	0	0	0	0
0	0	0	0	0	0	0	0	1	0	0	0	0	0	0	0
0	0	0	0	0	0	0	0	1	0	0	0	0	0	0	0
0	0	0	0	0	0	0	0	1	0	0	0	0	0	0	0
1	0	0	0	0	0	0	0	0	0	0	0	1	0	0	0
1	0	0	0	0	0	0	0	0	0	0	0	0	0	0	0
0	0	0	0	0	0	0	0	1	0	0	0	0	0	0	0
1	0	0	0	0	0	0	0	0	0	0	0	1	0	0	0
0	0	0	0	0	0	0	0	0	0	0	0	0	0	0	0
0	0	0	0	0	0	0	0	1	0	0	0	0	0	0	0
1	0	0	0	0	0	0	0	0	0	0	0	0	0	0	0
0	0	0	0	0	0	0	0	0	0	0	0	0	0	0	0
0	0	0	0	0	0	0	0	0	0	0	0	0	0	0	0
0	0	0	0	0	0	0	0	0	0	0	0	0	0	0	0
0	0	0	0	0	0	0	0	0	0	0	0	0	0	0	0
1	0	0	0	0	0	0	0	0	0	0	0	0	0	0	0
0	0	0	0	0	0	0	0	0	0	0	0	1	1	0	0
0	0	0	0	0	0	0	0	0	0	0	0	1	1	0	0
1	0	0	0	0	0	0	0	0	0	0	0	0	0	0	0
0	0	0	0	0	0	0	0	0	0	0	0	0	0	0	0
0	0	0	0	0	0	0	0	0	0	0	0	1	0	0	0
0	0	0	0	0	0	0	0	0	0	0	0	1	0	0	0
0	0	0	0	0	0	0	0	0	0	0	0	1	0	0	0
0	0	0	0	0	0	0	0	0	0	0	0	1	0	0	0
0	0	0	0	0	0	0	0	0	0	0	0	1	0	0	0
0	0	0	0	0	0	0	0	0	0	0	0	0	0	0	0
1	0	0	0	0	0	0	0	0	0	0	0	0	0	0	0
0	0	0	0	0	0	0	0	0	0	0	0	1	0	0	0
1	0	0	0	0	0	0	0	0	0	0	0	0	0	0	0
1	0	0	0	0	0	0	0	0	0	0	0	0	0	0	0
1	0	0	0	0	0	0	0	0	0	0	0	0	0	0	0
1	0	0	0	0	0	0	0	0	0	0	0	0	0	0	0
1	0	0	0	0	0	0	0	0	0	0	0	1	0	0	0
1	0	0	0	0	0	0	0	0	0	0	0	1	0	0	0
1	0	0	0	0	0	0	0	0	0	0	0	0	0	0	0
1	0	0	0	0	0	0	0	0	0	0	0	0	0	0	0
1	0	0	0	0	0	0	0	0	0	0	0	0	0	0	0
1	0	0	0	0	0	0	0	0	0	0	0	0	0	0	0

1	0	0	0	0	0	0	0	0	0	0	0	0	0	0	0
1	0	0	0	0	0	0	0	0	0	0	0	0	0	0	0
1	0	0	0	0	0	0	0	0	0	0	0	0	0	0	0
1	0	0	0	0	0	0	0	0	0	0	0	0	0	0	0
1	0	0	0	0	0	0	0	0	0	0	0	0	0	0	0
1	0	0	0	0	0	0	0	0	0	0	0	0	0	0	0
1	0	0	0	0	0	0	0	0	0	0	0	0	0	0	0
1	0	0	0	0	0	0	0	0	0	0	0	0	0	0	0
1	0	0	0	0	0	0	0	0	0	0	0	0	0	0	0
1	0	0	0	0	0	0	0	0	0	0	0	0	0	0	0
1	0	0	0	0	0	0	0	0	0	0	0	0	0	0	0
1	0	0	0	0	0	0	0	0	0	0	0	0	0	0	0
0	0	0	0	0	0	0	0	0	1	0	0	0	0	0	0
0	0	0	0	0	0	0	0	0	1	0	0	0	0	0	0
0	0	0	0	0	0	0	0	0	1	0	0	0	0	0	0
0	0	0	0	0	0	0	0	0	1	0	0	0	0	0	0
0	0	0	0	0	0	0	0	0	1	0	0	0	0	0	0
0	0	0	0	0	0	0	0	0	1	0	0	0	0	0	0
0	0	0	0	1	1	0	0	0	0	0	0	0	0	0	0
0	0	0	0	1	1	0	0	0	0	0	0	0	0	0	0
0	0	0	0	1	1	0	0	0	0	0	0	0	0	0	0
0	0	0	0	1	1	0	0	0	0	0	0	0	0	0	0
0	0	0	0	1	1	0	0	0	0	0	0	0	0	0	0
1	0	0	0	0	0	0	0	0	0	0	0	0	0	0	0
1	0	0	0	0	0	0	0	0	0	0	0	0	0	0	0
1	0	0	0	0	0	0	0	0	0	0	0	0	0	0	0
0	0	0	0	0	0	0	0	0	1	0	0	0	0	0	0
0	0	0	0	0	0	0	0	0	1	0	0	0	0	0	0
0	0	0	0	0	0	0	0	0	1	0	0	0	0	0	0
0	0	0	0	0	0	0	0	0	1	0	0	0	0	0	0
0	0	0	0	0	0	0	0	0	1	0	0	0	0	0	0
0	0	0	0	1	1	0	0	0	0	0	0	0	0	0	0
0	0	0	0	1	1	0	0	0	0	0	0	0	0	0	0
0	0	0	0	0	0	0	0	0	1	0	0	0	0	0	0
0	0	0	0	0	0	0	0	0	1	0	0	0	0	0	0
0	0	0	0	1	1	0	0	0	0	0	0	0	0	0	0
0	0	0	0	1	1	0	0	0	0	0	0	0	0	0	0
0	0	0	0	1	1	0	0	0	0	0	0	0	0	0	0
0	0	0	0	1	1	0	0	0	0	0	0	0	0	0	0
0	0	0	0	1	1	0	0	0	0	0	0	0	0	0	0
0	0	0	0	1	1	0	0	0	0	0	0	0	0	0	0
0	0	0	0	0	0	0	0	0	0	0	0	0	0	0	0
0	1	1	1	0	0	0	0	0	0	0	0	0	0	0	0

0	1	1	1	0	0	0	0	0	0	0	0	0	0	0	0
0	1	1	1	0	0	0	0	0	0	0	0	0	0	0	0
0	1	1	1	0	0	0	0	0	0	0	0	0	0	0	0
0	1	1	1	0	0	0	0	0	0	0	0	0	0	0	0
0	0	0	0	1	1	0	0	0	0	0	0	0	0	0	1
0	0	0	0	1	1	0	0	0	0	0	0	0	0	0	1
0	0	0	0	0	0	0	1	0	0	0	0	0	0	0	0
0	0	0	0	1	1	0	1	0	0	0	0	0	0	0	1
0	0	0	0	0	0	0	0	0	0	0	1	0	0	0	0
0	0	0	0	0	0	0	0	0	0	0	1	0	0	0	0
0	0	0	0	1	1	0	0	0	0	0	0	0	0	0	1
1	0	0	0	0	0	0	0	0	0	0	0	0	0	0	0
0	0	0	0	1	1	0	0	0	0	0	0	0	0	0	1
0	0	0	0	1	1	0	0	0	0	0	0	0	0	0	1
1	0	0	0	0	0	0	0	0	0	0	0	0	0	0	0
0	0	0	0	1	1	0	0	0	0	0	0	0	0	0	1
0	0	0	0	0	0	0	0	0	0	0	0	0	0	0	0
0	0	0	0	0	0	0	0	0	0	0	0	0	0	0	0
0	0	0	0	0	0	0	0	0	0	0	0	0	0	0	0
0	0	0	0	0	0	0	0	0	1	0	0	0	0	0	0
0	0	0	0	0	0	0	0	0	0	0	0	0	0	0	0
0	0	0	0	0	0	0	0	0	0	0	0	0	0	0	0
0	0	0	0	0	0	0	0	0	0	0	0	0	0	0	0
0	0	0	0	0	0	0	0	0	0	0	0	0	0	0	0

aac(3)-IId	aac(3)-IVa	aac(6')-Iaa	aac(6')-Ib-c	aac(6')-Ic	aadA16	aadA17	aadA2	aadA5	aadA7	ant(3")-Ia	aph(3")-Ib	aph(3')-Ia	aph(4)-Ia	aph(6)-Id
**aminoglycoside**														
aac(3)-IId	***aac(3)-IVa***	***aac(6')-Iaa***	***aac(6')-Ib-c***	***aac(6')-Ic***	***aadA16***	***aadA17***	***aadA2***	***aadA5***	***aadA7***	***ant(3")-Ia***	***aph(3")-Ib***	***aph(3')-Ia***	***aph(4)-Ia***	***aph(6)-Id***
0	0	1	0	0	0	0	0	0	0	1	0	1	0	0
0	0	1	0	0	0	0	0	0	0	0	0	0	0	1
0	0	1	0	0	0	0	0	0	0	0	0	1	0	1
0	0	1	0	0	0	0	0	0	0	0	0	1	0	1
0	0	1	0	0	0	0	0	0	0	0	1	0	0	1
0	0	1	0	0	0	0	0	0	0	1	0	0	0	0
0	0	1	0	0	0	0	0	0	0	0	0	0	0	1
0	0	1	0	0	0	0	0	0	0	0	1	0	0	1
0	0	1	0	0	0	0	0	0	0	0	0	0	0	0
0	0	1	0	0	0	0	0	0	0	0	0	0	0	1
0	0	1	0	0	0	0	0	0	0	0	0	0	0	0
1	0	1	0	0	0	0	0	0	1	0	0	0	0	0
1	0	1	0	0	0	0	0	0	1	0	0	0	0	0
1	0	1	0	0	0	1	0	0	1	0	0	0	0	0
1	0	1	0	0	0	1	0	0	1	0	0	0	0	0
0	0	1	0	0	0	0	0	0	0	0	0	0	0	0
1	0	1	0	0	0	0	0	0	0	0	0	0	0	0
1	0	1	0	0	0	0	0	0	0	0	0	0	0	0
0	0	1	0	0	0	0	0	0	0	0	1	1	0	1
1	0	1	0	0	0	1	0	0	1	0	0	0	0	0
1	0	1	0	0	0	0	0	0	0	0	0	0	0	0
1	0	1	0	0	0	0	0	0	0	0	0	1	0	0
1	0	1	0	0	0	0	0	0	0	0	0	1	0	0
1	0	1	0	0	0	0	0	0	0	0	0	0	0	0
1	0	1	0	0	0	0	0	0	0	0	0	0	0	0
1	0	1	0	0	0	0	0	0	1	0	0	0	0	0
0	0	1	0	0	0	0	0	0	0	0	1	1	0	1
1	0	1	0	0	0	0	0	0	0	0	0	1	0	0
0	0	1	0	0	0	0	0	0	0	0	1	1	0	1
0	0	1	0	0	0	0	0	0	0	0	1	0	0	1
0	0	1	0	0	0	0	0	0	0	0	1	1	0	1
0	0	1	0	0	0	0	0	0	0	0	1	1	0	1
0	0	1	0	0	0	0	0	0	0	0	1	0	0	1
0	0	1	0	0	0	0	0	0	0	0	1	0	0	1
0	0	1	0	0	0	0	0	0	0	0	0	1	0	0
0	0	1	0	0	0	0	0	0	0	0	0	1	0	0
0	0	1	0	0	0	0	0	0	0	0	0	1	0	0
0	0	1	0	0	0	0	0	0	0	0	0	1	0	0

0	0	1	0	0	0	0	0	0	0	0	0	1	0	0
0	0	1	0	0	0	0	0	0	0	0	0	1	0	0
0	0	1	0	0	0	0	0	0	0	0	0	0	0	0
0	0	1	0	0	0	0	0	0	0	0	0	0	0	0
0	0	1	0	0	0	0	0	0	0	0	0	0	0	0
0	0	1	0	0	0	0	0	0	0	0	0	0	0	0
0	0	1	0	0	0	0	0	0	0	0	0	0	0	0
0	0	1	0	0	0	0	0	0	0	0	0	0	0	0
0	0	1	0	0	0	0	0	0	0	0	0	0	0	0
0	0	1	0	0	0	0	0	0	0	0	0	0	0	0
0	0	1	0	0	0	0	0	0	0	0	0	0	0	0
0	0	1	0	0	0	0	0	0	0	0	0	0	0	0
1	1	1	0	0	0	1	0	0	1	0	0	0	0	0
1	1	1	0	0	0	0	0	0	1	0	0	1	0	0
1	1	1	0	0	0	0	0	0	1	0	0	1	0	0
1	1	1	0	0	0	0	0	0	1	0	0	1	0	0
1	1	1	0	0	0	0	0	0	1	0	0	1	0	0
1	1	1	0	0	0	0	0	0	1	0	0	1	0	0
0	1	1	1	0	0	0	0	0	0	0	0	1	1	0
0	1	1	1	0	0	0	0	0	0	0	0	0	1	0
0	1	1	1	0	0	0	0	0	0	0	0	0	1	0
0	1	1	1	0	0	0	0	0	0	0	0	0	1	0
0	1	1	1	0	0	0	0	0	0	0	0	0	1	0
0	0	1	0	0	0	0	0	0	0	0	0	0	0	0
0	0	1	0	0	0	0	0	0	0	0	1	0	0	1
0	0	1	0	0	0	0	0	0	0	0	1	0	0	1
1	1	1	0	0	0	0	0	0	1	0	0	1	0	0
1	1	1	0	0	0	0	0	0	1	0	0	1	0	0
1	1	1	0	0	0	0	0	0	1	0	0	1	0	0
1	1	1	0	0	0	0	0	0	1	0	0	1	0	0
1	1	1	0	0	0	0	0	0	1	0	0	1	0	0
0	1	1	1	0	0	0	0	0	0	0	0	0	1	0
0	1	1	1	0	0	0	0	0	0	0	0	0	1	0
1	1	1	0	0	0	0	0	0	1	0	0	1	0	0
1	1	1	0	0	0	0	0	0	1	0	0	1	0	0
0	1	1	1	0	0	0	1	0	0	1	0	1	1	0
0	1	1	1	0	0	0	1	0	0	1	0	1	1	0
0	1	1	1	0	0	0	1	0	0	1	0	1	1	0
0	0	1	0	0	0	0	0	0	0	0	0	0	0	0
0	1	1	1	0	0	0	0	0	0	1	0	1	1	0
0	1	1	1	0	0	0	1	0	0	1	0	1	1	0
0	0	1	0	0	0	0	0	0	0	0	0	0	0	0
0	0	1	0	0	0	0	0	0	0	0	0	0	0	0

0	0	1	0	0	0	0	0	0	0	0	0	0	0	0
0	0	1	0	0	0	0	0	0	0	0	0	0	0	0
0	0	1	0	0	0	0	0	0	0	0	0	0	0	0
0	0	1	0	0	0	0	0	0	0	0	0	0	0	0
0	0	0	1	0	0	0	0	0	0	1	0	0	0	0
0	0	0	1	0	0	0	0	0	0	1	0	0	0	0
0	0	1	0	0	0	0	0	0	0	0	0	0	0	0
0	0	1	1	0	0	0	0	0	0	1	0	0	0	0
0	0	1	0	0	0	0	0	1	0	0	0	0	0	0
0	0	1	0	0	0	0	0	1	0	0	0	0	0	0
0	0	0	1	0	0	0	0	0	0	1	0	0	0	0
0	0	1	0	0	0	0	0	0	0	0	0	0	0	0
0	0	1	1	0	0	0	0	0	0	1	0	0	0	0
0	0	0	1	0	0	0	0	0	0	1	0	0	0	0
0	0	1	0	0	0	0	0	0	0	1	0	0	0	0
0	0	0	1	0	0	0	0	0	0	1	0	0	0	0
0	0	1	0	0	0	0	0	0	0	0	0	0	0	0
1	0	1	0	0	0	0	0	0	1	0	0	0	0	0
0	0	1	0	0	0	0	0	0	0	0	1	0	0	1
0	0	1	0	0	0	0	1	0	0	0	1	0	0	1
0	0	1	0	0	0	0	0	0	0	0	0	0	0	0
1	0	1	0	0	0	0	0	0	0	0	0	0	0	0
1	0	1	0	0	0	1	0	0	1	0	0	0	0	0
0	0	1	0	0	0	0	0	0	0	0	1	0	0	1

rmtB	*bla* ACT-16	*bla* ACT-9	*bla* CMY-97	*bla* CTX-M-	*bla* CTX-M-	*bla* CTX-M-	*bla* DHA-12	*bla* OXA-10	*bla* OXA-1	*bla* SHV-145	*bla* SRT-2	*bla* TEM-14	*bla* TEM-1A
	**beta-lactam**												
rmtB	*bla* ACT-16	*bla* ACT-9	*bla* CMY-97	*bla* CTX-M-	*bla* CTX-M-	*bla* CTX-M-	*bla* DHA-12	*bla* OXA-10	*bla* OXA-1	*bla* SHV-145	*bla* SRT-2	*bla* TEM-14	*bla* TEM-1A
0	0	0	0	0	0	1	0	1	0	0	0	0	0
0	0	0	0	0	0	0	0	0	0	0	0	0	0
0	0	0	0	0	0	0	0	0	0	0	0	0	0
0	0	0	0	0	0	0	0	0	0	0	0	0	0
0	0	0	0	0	0	0	0	0	0	0	0	0	0
0	0	0	0	0	0	1	0	1	0	0	0	0	0
0	0	0	0	0	0	0	0	0	0	0	0	0	0
0	0	0	0	0	0	0	0	0	0	0	0	0	0
0	0	0	0	0	0	0	0	0	0	0	0	0	0
0	0	0	0	0	0	0	0	0	0	0	0	0	0
0	0	0	0	0	0	0	0	0	0	0	0	0	0
1	0	0	0	0	1	0	0	0	0	0	0	1	0
1	0	0	0	0	1	0	0	0	0	0	0	1	0
1	0	0	0	0	1	0	0	0	0	0	0	1	0
1	0	0	0	0	1	0	0	0	0	0	0	1	0
0	0	0	0	0	0	0	0	0	0	0	0	0	0
0	0	0	0	0	0	0	0	0	0	0	0	0	0
0	0	0	0	0	0	0	0	0	0	0	0	0	0
0	0	0	0	0	0	0	0	0	0	0	0	0	0
1	0	0	0	0	1	0	0	0	0	0	0	1	0
0	0	0	0	0	0	0	0	0	0	0	0	0	0
0	0	0	0	0	0	0	0	0	0	0	0	0	0
0	0	0	0	0	0	0	0	0	0	0	0	0	0
0	0	0	0	0	0	0	0	0	0	0	0	0	0
0	0	0	0	0	0	0	0	0	0	0	0	0	0
1	0	0	0	0	1	0	0	0	0	0	0	1	0
0	0	0	0	0	0	0	0	0	0	0	0	0	0
0	0	0	0	0	0	0	0	0	0	0	0	0	0
0	0	0	0	0	0	0	0	0	0	0	0	0	0
0	0	0	0	0	0	0	0	0	0	0	0	0	0
0	0	0	0	0	0	0	0	0	0	0	0	0	0
0	0	0	0	0	0	0	0	0	0	0	0	0	0
0	0	0	0	0	0	0	0	0	0	0	0	0	0
0	0	0	0	0	0	0	0	0	0	0	0	0	0
0	0	0	0	0	0	0	0	0	0	0	0	0	0
0	0	0	0	0	0	0	0	0	0	0	0	0	0
0	0	0	0	0	0	0	0	0	0	0	0	0	0
0	0	0	0	0	0	0	0	0	0	0	0	0	0

0	0	0	0	0	0	0	0	0	0	0	0	0	0
0	0	0	0	0	0	0	0	0	0	0	0	0	0
0	0	0	0	0	0	0	0	0	0	0	0	0	0
0	0	0	0	0	0	0	0	0	0	0	0	0	0
0	0	0	0	0	0	0	0	0	0	0	0	0	0
0	0	0	0	0	0	0	0	0	0	0	0	0	0
0	0	0	0	0	0	0	0	0	0	0	0	0	0
0	0	0	0	0	0	0	0	0	0	0	0	0	0
0	0	0	0	0	0	0	0	0	0	0	0	0	0
0	0	0	0	0	0	0	0	0	0	0	0	0	0
0	0	0	0	0	0	0	0	0	0	0	0	0	0
0	0	0	0	0	0	0	0	0	0	0	0	0	0
0	0	0	0	0	1	0	0	0	0	0	0	0	0
0	0	0	0	0	1	0	0	0	0	0	0	0	0
0	0	0	0	0	1	0	0	0	0	0	0	0	0
0	0	0	0	0	1	0	0	0	0	0	0	0	0
0	0	0	0	0	1	0	0	0	0	0	0	0	0
0	0	0	0	0	1	0	0	0	0	0	0	0	0
0	0	0	0	0	0	0	0	0	1	0	0	0	0
0	0	0	0	0	0	0	0	0	1	0	0	0	0
0	0	0	0	0	0	0	0	0	1	0	0	0	0
0	0	0	0	0	0	0	0	0	1	0	0	0	0
0	0	0	0	0	0	0	0	0	1	0	0	0	0
0	0	0	0	0	0	0	0	0	0	0	0	0	0
0	0	0	0	0	0	0	0	0	0	0	0	0	0
0	0	0	0	0	0	0	0	0	0	0	0	0	0
0	0	0	0	0	1	0	0	0	0	0	0	0	0
0	0	0	0	0	1	0	0	0	0	0	0	0	0
0	0	0	0	0	1	0	0	0	0	0	0	0	0
0	0	0	0	0	1	0	0	0	0	0	0	0	0
0	0	0	0	0	1	0	0	0	0	0	0	0	0
0	0	0	0	0	0	0	0	0	1	0	0	0	0
0	0	0	0	0	0	0	0	0	1	0	0	0	0
0	0	0	0	0	1	0	0	0	0	0	0	0	0
0	0	0	0	0	1	0	0	0	0	0	0	0	0
0	0	0	0	0	0	0	0	0	1	0	0	0	0
0	0	0	0	0	0	0	0	0	1	0	0	0	0
0	0	0	0	0	0	0	0	0	1	0	0	0	0
0	0	0	0	0	0	0	0	0	0	0	0	0	0
0	0	0	0	0	0	0	0	0	1	0	0	0	0
0	0	0	0	0	0	0	0	0	1	0	0	0	0
0	0	0	0	0	0	0	0	0	0	0	0	0	0
0	0	0	0	0	0	0	0	0	0	0	0	0	0

0	0	0	0	0	0	0	0	0	0	0	0	0	0
0	0	0	0	0	0	0	0	0	0	0	0	0	0
0	0	0	0	0	0	0	0	0	0	0	0	0	0
0	0	0	0	0	0	0	0	0	0	0	0	0	0
0	0	0	0	0	0	0	0	0	1	0	0	0	0
0	0	0	0	0	0	0	0	0	1	0	0	0	0
0	0	0	0	0	0	0	0	0	0	0	0	0	1
0	0	0	0	0	0	0	0	0	1	0	0	0	1
0	0	0	0	0	0	0	0	0	0	0	0	0	0
0	0	0	0	0	0	0	0	0	0	0	0	0	0
0	0	0	0	0	0	0	0	0	1	0	0	0	0
0	0	0	0	0	0	0	0	0	0	0	0	0	0
0	0	0	0	0	0	0	0	0	1	0	0	0	0
0	0	0	0	0	0	0	0	0	1	0	0	0	0
0	0	0	0	0	0	1	0	1	0	0	0	0	0
0	0	0	0	0	0	0	0	0	1	0	0	0	0
0	0	0	0	0	0	0	0	0	0	0	0	0	0
0	0	0	0	0	1	0	0	0	0	0	0	1	0
0	0	0	0	0	0	0	0	0	0	0	0	0	0
0	0	0	0	0	0	0	0	0	0	1	0	0	0
0	0	0	0	0	0	0	0	0	0	0	0	0	0
0	0	0	0	0	1	0	0	0	0	0	0	1	0
0	0	0	0	0	1	0	0	0	0	0	0	1	0
0	0	0	0	0	0	0	0	0	0	0	0	0	0

bla TEM-1E	*catA1*	*catB3*	*cmlA1*	*floR*	*dfrA12*	*dfrA14*	*dfrA17*	*dfrA1*	*dfrA27*	*fosA3*	*fosA7*	*fosA*	*lnu(F)*	*mph(A)*	*erm(T)*	*oqxA*
	**phenicol**				**trimethoprim**					**fosfomycin**			**macrolide**			**quinolon**
***bla* TEM-1E**	***catA1***	***catB3***	***cmlA1***	***floR***	***dfrA12***	***dfrA14***	***dfrA17***	***dfrA1***	***dfrA27***	***fosA3***	***fosA7***	***fosA***	***lnu(F)***	***mph(A)***	***erm(T)***	***oqxA***
0	0	0	1	1	0	1	0	0	0	0	0	0	0	0	0	0
0	0	0	0	1	0	1	0	0	0	0	0	0	0	0	0	0
0	0	0	0	1	0	1	0	0	0	0	0	0	0	0	0	0
0	0	0	0	1	0	1	0	0	0	0	0	0	0	0	0	0
1	0	0	0	0	0	0	0	0	0	0	0	0	0	0	0	0
0	0	0	1	1	0	1	0	0	0	0	0	0	0	0	0	0
0	0	0	0	1	0	1	0	0	0	0	0	0	0	0	0	0
1	0	0	0	0	0	0	0	0	0	0	0	0	0	0	0	0
0	0	0	0	0	0	0	0	0	0	0	0	0	0	0	0	0
0	0	0	0	1	0	1	0	0	0	0	0	0	0	0	0	0
0	0	0	0	0	0	0	0	0	0	0	0	0	0	0	0	0
0	0	0	0	1	0	1	0	0	0	1	0	0	1	1	0	0
0	0	0	0	1	0	1	0	0	0	1	0	0	1	1	0	0
0	0	0	0	1	0	1	0	0	0	1	0	0	1	1	0	0
0	0	0	0	1	0	1	0	0	0	1	0	0	1	1	0	0
0	0	0	0	0	0	0	0	0	0	0	0	0	0	0	0	0
1	0	0	0	0	0	0	0	0	0	0	0	0	0	0	0	0
1	0	0	0	0	0	0	0	0	0	0	0	0	0	0	0	0
1	0	0	0	0	0	0	0	0	0	0	0	0	0	0	0	0
0	0	0	0	1	0	1	0	0	0	1	0	1	1	1	0	0
1	0	0	0	0	0	0	0	0	0	0	0	0	0	0	0	0
1	0	0	0	0	0	0	0	0	0	0	0	0	0	0	0	0
1	0	0	0	0	0	0	0	0	0	0	0	0	0	0	0	0
1	0	0	0	0	0	0	0	0	0	0	0	0	0	0	0	0
1	0	0	0	0	0	0	0	0	0	0	0	0	0	0	0	0
0	0	0	0	1	0	1	0	0	0	1	0	0	0	1	0	0
1	0	0	0	0	0	0	0	0	0	0	0	0	0	0	0	0
1	0	0	0	0	0	0	0	0	0	0	0	0	0	0	0	0
1	0	0	0	0	0	0	0	0	0	0	0	0	0	0	0	0
1	0	0	0	0	0	0	0	0	0	0	0	0	0	0	0	0
1	0	0	0	0	0	0	0	0	0	0	0	0	0	0	0	0
1	0	0	0	0	0	0	0	0	0	0	0	0	0	0	0	0
1	0	0	0	0	0	0	0	0	0	0	0	0	0	0	0	0
1	0	0	0	0	0	0	0	0	0	0	0	0	0	0	0	0
0	0	0	0	0	0	0	0	0	0	0	0	0	0	0	0	0
0	0	0	0	0	0	0	0	0	0	0	0	0	0	0	0	0
0	0	0	0	0	0	0	0	0	0	0	0	0	0	0	0	0
0	0	0	0	0	0	0	0	0	0	0	0	0	0	0	0	0

0	0	0	0	0	0	0	0	0	0	0	0	0	0	0	0	0
0	0	0	0	0	0	0	0	0	0	0	0	0	0	0	0	0
0	0	0	0	0	0	0	0	0	0	0	0	0	0	0	0	0
0	0	0	0	0	0	0	0	0	0	0	0	0	0	0	0	0
0	0	0	0	0	0	0	0	0	0	0	0	0	0	0	0	0
0	0	0	0	0	0	0	0	0	0	0	0	0	0	0	0	0
0	0	0	0	0	0	0	0	0	0	0	0	0	0	0	0	0
0	0	0	0	0	0	0	0	0	0	0	0	0	0	0	0	0
0	0	0	0	0	0	0	0	0	0	0	0	0	0	0	0	0
0	0	0	0	0	0	0	0	0	0	0	0	0	0	0	0	0
0	0	0	0	0	0	0	0	0	0	0	0	0	0	0	0	0
0	0	0	0	0	0	0	0	0	0	0	0	0	0	0	0	0
1	0	0	0	1	0	1	0	0	0	0	0	0	1	1	0	0
1	0	0	0	1	0	1	0	0	0	0	0	0	1	1	0	0
1	0	0	0	1	0	1	0	0	0	0	0	0	1	1	0	0
1	0	0	0	1	0	1	0	0	0	0	0	0	1	1	0	0
1	0	0	0	1	0	1	0	0	0	0	0	0	1	1	0	0
1	0	0	0	1	0	1	0	0	0	0	0	0	1	1	0	0
0	0	1	0	1	1	0	0	0	0	0	0	0	0	0	0	0
0	0	1	0	1	1	0	0	0	0	0	0	0	0	0	0	0
0	0	1	0	1	1	0	0	0	0	0	0	0	0	0	0	0
0	0	1	0	1	1	0	0	0	0	0	0	0	0	0	0	0
0	0	1	0	1	1	0	0	0	0	0	0	0	0	0	0	0
0	0	0	0	0	0	0	0	0	0	0	0	0	0	0	0	0
1	0	0	0	0	0	0	0	0	0	0	0	0	0	0	0	0
1	0	0	0	0	0	0	0	0	0	0	0	0	0	0	0	0
1	0	0	0	1	0	1	0	0	0	0	0	0	1	1	0	0
1	0	0	0	1	0	1	0	0	0	0	0	0	1	1	0	0
1	0	0	0	1	0	1	0	0	0	0	0	0	1	1	0	0
1	0	0	0	1	0	1	0	0	0	0	0	0	1	1	0	0
1	0	0	0	1	0	1	0	0	0	0	0	0	1	1	0	0
0	0	1	0	1	1	0	0	0	0	0	0	0	0	0	0	0
0	0	1	0	1	1	0	0	0	0	0	0	0	0	0	0	0
1	0	0	0	1	0	1	0	0	0	0	0	0	1	1	0	0
1	0	0	0	1	0	1	0	0	0	0	0	0	1	1	0	0
0	0	1	1	1	1	0	0	0	0	0	0	0	0	0	0	1
0	0	1	1	1	1	0	0	0	0	0	0	0	0	0	0	1
0	0	1	1	1	1	0	0	0	0	0	0	0	0	0	0	1
0	0	0	0	0	0	0	0	0	0	0	0	0	0	0	0	1
0	0	1	1	1	1	0	0	0	0	0	0	0	0	0	0	1
0	0	1	1	1	1	0	0	0	0	0	0	0	0	0	0	1
0	0	0	0	1	0	0	0	0	0	0	0	0	0	0	0	0
0	0	0	0	1	0	0	0	0	0	0	1	0	0	0	0	0

0	0	0	0	1	0	0	0	0	0	0	1	0	0	0	0	0
0	0	0	0	1	0	0	0	0	0	0	1	0	0	0	0	0
0	0	0	0	1	0	0	0	0	0	0	1	0	0	0	0	0
0	0	0	0	1	0	0	0	0	0	0	1	0	0	0	0	0
0	0	1	0	1	0	0	0	1	0	0	0	0	0	0	0	0
0	0	1	0	1	0	0	0	1	0	0	0	0	0	0	0	0
0	0	0	0	1	0	0	0	0	0	0	1	0	0	0	0	1
0	0	1	0	1	0	0	0	1	0	0	1	0	0	0	0	1
1	0	0	0	0	0	0	1	0	0	0	0	0	0	0	0	0
1	0	0	0	0	0	0	1	0	0	0	0	0	0	0	0	0
0	0	1	0	1	0	0	0	1	0	0	0	0	0	0	0	0
0	0	0	0	0	0	0	0	0	0	0	0	0	0	0	0	0
0	0	1	0	1	0	0	0	1	0	0	0	0	0	0	0	0
0	0	1	0	1	0	0	0	1	0	0	0	0	0	0	0	0
0	0	0	1	1	0	1	0	0	0	0	0	0	0	0	0	0
0	0	1	0	1	0	0	0	1	0	0	0	0	0	0	0	0
0	0	0	0	0	0	0	0	0	0	0	0	0	0	0	0	0
0	0	0	0	1	0	1	0	0	0	0	0	0	1	1	0	0
0	0	0	0	1	0	0	0	0	0	0	0	0	0	0	0	0
0	0	0	0	1	1	0	0	0	0	0	0	0	0	0	0	1
0	0	0	0	0	0	0	0	0	0	0	0	0	0	0	0	0
0	0	0	0	1	0	1	0	0	0	0	0	0	1	1	0	0
0	0	0	0	1	0	1	0	0	0	0	0	0	1	1	0	0
0	0	0	0	1	0	0	0	0	0	0	0	0	0	0	0	0

oqxB	qnrB6	qnrS1	qnrS2	sul1	sul2	sul3	tet (A)	tet (B)	tet (D)	tet (M)	ARR-3	VFDB	cdtB	spvB	csgA
ne				sulphonamide			tetracycline				rifampicin		Typhoid toxi	SpvB	Agf/Csg
oqxB	*qnrB6*	*qnrS1*	*qnrS2*	*sul1*	*sul2*	*sul3*	*tet* (A)	*tet* (B)	*tet* (D)	*tet* (M)	*ARR-3*	B_NUM_FO	*cdtB*	*spvB*	*csgA*
0	0	1	0	0	0	0	0	0	0	0	1	106	0	1	1
0	0	1	0	0	0	0	1	0	0	0	0	91	0	0	1
0	0	1	0	0	0	0	1	0	0	0	0	91	0	0	1
0	0	1	0	0	0	0	1	0	0	0	0	91	0	0	1
0	0	0	0	0	1	0	0	0	0	0	0	107	0	1	1
0	0	1	0	0	0	0	0	0	0	0	1	106	0	1	1
0	0	1	0	0	0	0	1	0	0	0	0	91	0	0	1
0	0	0	0	0	1	0	0	0	0	0	0	106	0	1	1
0	0	0	0	0	0	0	1	0	0	0	0	90	1	0	1
0	0	1	0	0	0	0	1	0	0	0	0	91	0	0	1
0	0	0	0	0	0	0	0	0	0	0	0	112	0	1	1
0	0	1	0	1	0	0	1	0	0	0	1	93	0	0	1
0	0	1	0	1	0	0	1	0	0	0	1	92	0	0	1
0	0	1	0	1	0	0	1	0	0	0	1	92	0	0	1
0	0	1	0	1	0	0	1	0	0	0	1	92	0	0	1
0	0	0	0	0	0	0	0	0	0	0	0	111	0	1	1
0	0	0	0	0	0	0	1	0	0	0	0	100	0	0	1
0	0	0	0	0	0	0	1	0	0	0	0	100	0	0	1
0	0	0	0	0	0	0	1	0	0	0	0	111	0	1	1
0	0	1	0	1	0	0	1	0	0	0	1	92	0	0	1
0	0	0	0	0	0	0	1	0	0	0	0	101	0	0	1
0	0	0	0	0	0	0	1	0	0	0	0	100	0	0	1
0	0	0	0	0	0	0	1	0	0	0	0	100	0	0	1
0	0	0	0	0	0	0	1	0	0	0	0	100	0	0	1
0	0	0	0	0	0	0	1	0	0	0	0	100	0	0	1
0	0	1	0	1	0	0	1	0	0	0	1	92	0	0	1
0	0	0	0	0	0	0	1	0	0	0	0	111	0	1	1
0	0	0	0	0	0	0	1	0	0	0	0	100	0	0	1
0	0	0	0	0	0	0	1	0	0	0	0	111	0	1	1
0	0	0	0	0	0	0	1	0	0	0	0	111	0	1	1
0	0	0	0	0	0	0	1	0	0	0	0	111	0	1	1
0	0	0	0	0	0	0	1	0	0	0	0	112	0	1	1
0	0	0	0	0	1	0	1	0	0	0	0	107	0	1	1
0	0	0	0	0	1	0	1	0	0	0	0	106	0	1	1
0	0	0	0	0	0	0	0	0	0	0	0	96	0	0	1
0	0	0	0	0	0	0	0	0	0	0	0	96	0	0	1
0	0	0	0	0	0	0	0	0	0	0	0	95	0	0	1
0	0	0	0	0	0	0	0	0	0	0	0	96	0	0	1

0	0	0	0	0	0	0	0	0	0	0	0	95	0	0	1
0	0	0	0	0	0	0	0	0	0	0	0	96	0	0	1
0	0	0	0	0	0	0	0	0	0	0	0	96	0	0	1
0	0	0	0	0	0	0	0	0	0	0	0	95	0	0	1
0	0	0	0	0	0	0	0	0	0	0	0	96	0	0	1
0	0	0	0	0	0	0	0	0	0	0	0	95	0	0	1
0	0	0	0	0	0	0	0	0	0	0	0	95	0	0	1
0	0	0	0	0	0	0	0	0	0	0	0	94	0	0	1
0	0	0	0	0	0	0	0	0	0	0	0	95	0	0	1
0	0	0	0	0	0	0	0	0	0	0	0	96	0	0	1
0	0	0	0	0	0	0	0	0	0	0	0	96	0	0	1
0	0	0	0	0	0	0	0	0	0	0	0	96	0	0	1
0	0	1	0	1	0	0	1	0	0	0	1	92	0	0	1
0	0	1	0	1	0	0	1	0	0	0	1	92	0	0	1
0	0	1	0	1	0	0	1	0	0	0	1	92	0	0	1
0	0	1	0	1	0	0	1	0	0	0	1	92	0	0	1
0	0	1	0	1	0	0	1	0	0	0	1	92	0	0	1
0	0	1	0	1	0	0	1	0	0	0	1	92	0	0	1
0	0	0	0	1	1	0	0	1	0	0	1	102	0	0	1
0	0	0	0	1	1	0	0	1	0	0	1	102	0	0	1
0	0	0	0	1	1	0	0	1	0	0	1	102	0	0	1
0	0	0	0	1	1	0	0	1	0	0	1	102	0	0	1
0	0	0	0	1	1	0	0	1	0	0	1	102	0	0	1
0	0	0	0	0	0	0	0	0	0	0	0	96	0	0	1
0	0	0	0	0	0	0	1	0	0	0	0	111	0	1	1
0	0	0	0	0	0	0	1	0	0	0	0	111	0	1	1
0	0	1	0	1	0	0	1	0	0	0	1	92	0	0	1
0	0	1	0	1	0	0	1	0	0	0	1	92	0	0	1
0	0	1	0	1	0	0	1	0	0	0	1	92	0	0	1
0	0	1	0	1	0	0	1	0	0	0	1	92	0	0	1
0	0	1	0	1	0	0	1	0	0	0	1	92	0	0	1
0	0	0	0	1	1	0	0	1	0	0	1	102	0	0	1
0	0	0	0	1	1	0	0	1	0	0	1	102	0	0	1
0	0	1	0	1	0	0	1	0	0	0	1	92	0	0	1
0	0	1	0	1	0	0	1	0	0	0	1	92	0	0	1
1	0	0	0	1	1	1	0	1	0	0	1	102	0	0	1
1	0	0	0	1	1	1	0	1	0	0	1	102	0	0	1
1	0	0	0	1	1	1	0	1	0	0	1	102	0	0	1
1	0	0	0	1	0	0	0	1	0	0	0	103	0	0	1
1	0	0	0	1	1	1	0	1	0	0	1	102	0	0	1
1	0	0	0	1	1	1	0	1	0	0	1	102	0	0	1
0	0	1	0	0	1	0	1	0	0	1	0	91	0	0	1
0	0	1	0	0	0	0	1	0	0	0	0	92	0	0	1

0	0	1	0	0	0	0	1	0	0	0	0	92	0	0	1
0	0	1	0	0	0	0	1	0	0	0	0	92	0	0	1
0	0	1	0	0	0	0	1	0	0	0	0	92	0	0	1
0	0	1	0	0	0	0	1	0	0	0	0	92	0	0	1
0	0	0	0	1	0	0	1	0	0	0	1	89	1	0	1
0	0	0	0	1	0	0	1	0	0	0	1	89	1	0	1
1	0	1	0	0	0	0	0	0	0	0	0	90	0	0	1
1	0	1	0	1	0	0	1	0	0	0	1	80	1	0	1
0	0	0	0	0	0	0	0	0	0	0	0	100	0	0	1
0	0	0	0	0	0	0	0	0	0	0	0	100	0	0	1
0	0	0	0	1	0	0	1	0	0	0	1	89	1	0	1
0	0	0	0	0	0	0	0	0	0	0	0	112	0	1	1
0	0	0	0	1	0	0	0	0	0	0	1	89	1	0	1
0	0	0	0	1	0	0	1	0	0	0	1	89	1	0	1
0	0	1	0	0	0	0	0	0	0	0	1	106	0	1	1
0	0	0	0	1	0	0	1	0	0	0	1	89	1	0	1
0	0	1	0	0	0	0	1	0	0	0	0	90	0	0	1
0	0	0	0	1	0	0	1	0	0	0	1	92	0	0	1
0	0	1	0	0	1	0	1	0	0	0	0	92	0	0	1
1	0	1	0	1	1	0	1	0	0	0	0	89	0	0	1
0	0	1	0	0	0	0	1	0	0	0	0	89	0	0	1
0	0	0	0	0	0	0	0	0	0	0	1	92	0	0	1
0	0	0	0	1	0	0	1	0	0	0	1	92	0	0	1
0	0	1	0	0	1	0	1	0	0	0	0	89	0	0	1

csgB	csgC	csgD	csgE	csgF	csgG	fimC	fimD	fimF	fimH	fimI	lpfA	lpfB	lpfC	lpfD	lpfE	pefA	pefB	pefC	pefD
						FIM					Lpf					Pef			
csgB	csgC	csgD	csgE	csgF	csgG	fimC	fimD	fimF	fimH	fimI	lpfA	lpfB	lpfC	lpfD	lpfE	pefA	pefB	pefC	pefD
1	1	1	1	1	1	1	1	1	1	1	1	1	1	1	1	0	0	0	0
1	1	1	1	1	1	1	1	1	1	1	1	1	1	0	1	0	0	0	0
1	1	1	1	1	1	1	1	1	1	1	1	1	1	0	1	0	0	0	0
1	1	1	1	1	1	1	1	1	1	1	1	1	1	0	1	0	0	0	0
1	1	1	1	1	1	1	1	1	1	1	1	1	1	1	1	0	1	1	1
1	1	1	1	1	1	1	1	1	1	1	1	1	1	1	1	0	0	0	0
1	1	1	1	1	1	1	1	1	1	1	1	1	1	0	1	0	0	0	0
1	1	1	1	1	1	1	1	1	1	1	1	1	1	1	1	0	1	1	1
1	1	1	1	1	1	1	1	1	1	1	0	0	0	0	0	0	0	0	0
1	1	1	1	1	1	1	1	1	1	1	1	1	1	0	1	0	0	0	0
1	1	1	1	1	1	1	1	1	1	1	1	1	1	1	1	1	1	1	1
1	1	1	1	1	1	1	1	1	1	1	1	1	1	1	1	0	1	0	0
1	1	1	1	1	1	1	1	1	1	1	1	1	1	1	1	0	0	0	0
1	1	1	1	1	1	1	1	1	1	1	1	1	1	1	1	0	0	0	0
1	1	1	1	1	1	1	1	1	1	1	1	1	1	1	1	0	0	0	0
1	1	1	1	1	1	1	1	1	1	1	1	1	1	1	1	1	1	1	1
1	1	1	1	1	1	1	1	1	1	1	1	1	1	1	1	0	0	0	0
1	1	1	1	1	1	1	1	1	1	1	1	1	1	1	1	0	0	0	0
1	1	1	1	1	1	1	1	1	1	1	1	1	1	1	1	1	1	1	1
1	1	1	1	1	1	1	1	1	1	1	1	1	1	1	1	0	0	0	0
1	1	1	1	1	1	1	1	1	1	1	1	1	1	1	1	0	0	0	0
1	1	1	1	1	1	1	1	1	1	1	1	1	1	1	1	0	0	0	0
1	1	1	1	1	1	1	1	1	1	1	1	1	1	1	1	0	0	0	0
1	1	1	1	1	1	1	1	1	1	1	1	1	1	1	1	0	0	0	0
1	1	1	1	1	1	1	1	1	1	1	1	1	1	1	1	0	0	0	0
1	1	1	1	1	1	1	1	1	1	1	1	1	1	1	1	0	0	0	0
1	1	1	1	1	1	1	1	1	1	1	1	1	1	1	1	1	1	1	1
1	1	1	1	1	1	1	1	1	1	1	1	1	1	1	1	0	0	0	0
1	1	1	1	1	1	1	1	1	1	1	1	1	1	1	1	1	1	1	1
1	1	1	1	1	1	1	1	1	1	1	1	1	1	1	1	1	1	1	1
1	1	1	1	1	1	1	1	1	1	1	1	1	1	1	1	1	1	1	1
1	1	1	1	1	1	1	1	1	1	1	1	1	1	1	1	1	1	1	1
1	1	1	1	1	1	1	1	1	1	1	1	1	1	1	1	0	1	1	1
1	1	1	1	1	1	1	1	1	1	1	1	1	1	1	1	0	1	1	1
1	1	1	1	1	1	1	1	1	1	1	0	0	0	0	0	0	0	0	0
1	1	1	1	1	1	1	1	1	1	1	0	0	0	0	0	0	0	0	0
1	1	1	1	1	1	1	1	1	1	1	0	0	0	0	0	0	0	0	0
1	1	1	1	1	1	1	1	1	1	1	0	0	0	0	0	0	0	0	0

1	1	1	1	1	1	1	1	1	1	1	0	0	0	0	0	0	0	0	0
1	1	1	1	1	1	1	1	1	1	1	0	0	0	0	0	0	0	0	0
1	1	1	1	1	1	1	1	1	1	1	0	0	0	0	0	0	0	0	0
1	1	1	1	1	1	1	1	1	1	1	0	0	0	0	0	0	0	0	0
1	1	1	1	1	1	1	1	1	1	1	0	0	0	0	0	0	0	0	0
1	1	1	1	1	1	1	1	1	1	1	0	0	0	0	0	0	0	0	0
1	1	1	1	1	1	1	1	1	1	1	0	0	0	0	0	0	0	0	0
1	1	1	1	1	1	1	1	1	1	1	0	0	0	0	0	0	0	0	0
1	1	1	1	1	1	1	1	1	1	1	0	0	0	0	0	0	0	0	0
1	1	1	1	1	1	1	1	1	1	1	0	0	0	0	0	0	0	0	0
1	1	1	1	1	1	1	1	1	1	1	0	0	0	0	0	0	0	0	0
1	1	1	1	1	1	1	1	1	1	1	0	0	0	0	0	0	0	0	0
1	1	1	1	1	1	1	1	1	1	1	1	1	1	1	1	0	0	0	0
1	1	1	1	1	1	1	1	1	1	1	1	1	1	1	1	0	0	0	0
1	1	1	1	1	1	1	1	1	1	1	1	1	1	1	1	0	0	0	0
1	1	1	1	1	1	1	1	1	1	1	1	1	1	1	1	0	0	0	0
1	1	1	1	1	1	1	1	1	1	1	1	1	1	1	1	0	0	0	0
1	1	1	1	1	1	1	1	1	1	1	1	1	1	1	1	0	0	0	0
1	1	1	1	1	1	1	1	1	1	1	1	1	1	1	1	0	0	0	0
1	1	1	1	1	1	1	1	1	1	1	1	1	1	1	1	0	0	0	0
1	1	1	1	1	1	1	1	1	1	1	1	1	1	1	1	0	0	0	0
1	1	1	1	1	1	1	1	1	1	1	1	1	1	1	1	0	0	0	0
1	1	1	1	1	1	1	1	1	1	1	1	1	1	1	1	0	0	0	0
1	1	1	1	1	1	1	1	1	1	1	0	0	0	0	0	0	0	0	0
1	1	1	1	1	1	1	1	1	1	1	1	1	1	1	1	1	1	1	1
1	1	1	1	1	1	1	1	1	1	1	1	1	1	1	1	1	1	1	1
1	1	1	1	1	1	1	1	1	1	1	1	1	1	1	1	0	0	0	0
1	1	1	1	1	1	1	1	1	1	1	1	1	1	1	1	0	0	0	0
1	1	1	1	1	1	1	1	1	1	1	1	1	1	1	1	0	0	0	0
1	1	1	1	1	1	1	1	1	1	1	1	1	1	1	1	0	0	0	0
1	1	1	1	1	1	1	1	1	1	1	1	1	1	1	1	0	0	0	0
1	1	1	1	1	1	1	1	1	1	1	1	1	1	1	1	0	0	0	0
1	1	1	1	1	1	1	1	1	1	1	1	1	1	1	1	0	0	0	0
1	1	1	1	1	1	1	1	1	1	1	1	1	1	1	1	0	0	0	0
1	1	1	1	1	1	1	1	1	1	1	1	1	1	1	1	0	0	0	0
1	1	1	1	1	1	1	1	1	1	1	1	1	1	1	1	0	0	0	0
1	1	1	1	1	1	1	1	1	1	1	1	1	1	1	1	0	0	0	0
1	1	1	1	1	1	1	1	1	1	1	1	1	1	1	1	0	0	0	0
1	1	1	1	1	1	1	1	1	1	1	1	1	1	1	1	0	0	0	0
1	1	1	1	1	1	1	1	1	1	1	1	1	1	1	1	0	0	0	0
1	1	1	1	1	1	1	1	1	1	1	1	1	1	1	1	0	0	0	0
1	1	1	1	1	1	1	1	1	1	1	1	1	1	0	1	0	0	0	0
1	1	1	1	1	1	1	1	1	1	1	1	1	1	0	1	0	0	0	0

1	1	1	1	1	1	1	1	1	1	1	1	1	1	0	1	0	0	0	0
1	1	1	1	1	1	1	1	1	1	1	1	1	1	0	1	0	0	0	0
1	1	1	1	1	1	1	1	1	1	1	1	1	1	0	1	0	0	0	0
1	1	1	1	1	1	1	1	1	1	1	1	1	1	0	1	0	0	0	0
1	1	1	1	1	1	1	1	1	1	1	0	0	0	0	0	0	0	0	0
1	1	1	1	1	1	1	1	1	1	1	0	0	0	0	0	0	0	0	0
1	1	1	1	1	1	1	1	1	1	1	1	1	1	0	1	0	0	0	0
1	1	1	1	1	1	1	1	1	0	1	1	1	1	0	1	0	0	0	0
1	1	1	1	1	1	1	1	1	1	1	1	1	1	1	1	0	0	0	0
1	1	1	1	1	1	1	1	1	1	1	1	1	1	1	1	0	0	0	0
1	1	1	1	1	1	1	1	1	1	1	0	0	0	0	0	0	0	0	0
1	1	1	1	1	1	1	1	1	1	1	1	1	1	1	1	1	1	1	1
1	1	1	1	1	1	1	1	1	1	1	0	0	0	0	0	0	0	0	0
1	1	1	1	1	1	1	1	1	1	1	0	0	0	0	0	0	0	0	0
1	1	1	1	1	1	1	1	1	1	1	1	1	1	1	1	0	0	0	0
1	1	1	1	1	1	1	1	1	1	1	0	0	0	0	0	0	0	0	0
1	1	1	1	1	1	1	1	1	1	1	0	0	0	0	0	0	0	0	0
1	1	1	1	1	1	1	1	1	1	1	1	1	1	1	1	0	0	0	0
1	1	1	1	1	1	1	1	1	1	1	1	1	1	0	1	0	0	0	0
1	1	1	1	1	1	1	1	1	1	1	0	0	0	0	0	0	0	0	0
1	1	1	1	1	1	1	1	1	1	1	0	0	0	0	0	0	0	0	0
1	1	1	1	1	1	1	1	1	1	1	1	1	1	1	1	0	0	0	0
1	1	1	1	1	1	1	1	1	1	1	1	1	1	1	1	0	0	0	0
1	1	1	1	1	1	1	1	1	1	1	0	0	0	0	0	0	0	0	0

steA	*steB*	*steC*	*mgtB*	*mgtC*	*rck*	*sodCI*	*mig-14*	*sinH*	*shdA*	*ratB*	*misL*	*gogB*	*spvC*	*spvR*	*sifA*	*sifB*	*sseF*
**Ste**			**Mg2+ transport**		**rck**	**SodCI**	**Mig-14**	**sinH**	**ShdA**	**ratB**	**MisL**	**anslocated effectors**					
steA	***steB***	***steC***	***mgtB***	***mgtC***	***rck***	***sodCI***	***mig-14***	***sinH***	***shdA***	***ratB***	***misL***	***gogB***	***spvC***	***spvR***	***sifA***	***sifB***	***sseF***
1	1	1	1	1	0	1	1	1	0	1	1	1	1	1	1	1	1
0	1	1	1	1	0	0	1	1	0	0	1	0	0	0	1	1	1
0	1	1	1	1	0	0	1	1	0	0	1	0	0	0	1	1	1
0	1	1	1	1	0	0	1	1	0	0	1	0	0	0	1	1	1
1	1	1	1	1	1	1	1	1	1	1	1	0	1	1	1	1	1
1	1	1	1	1	0	1	1	1	0	1	1	1	1	1	1	1	1
0	1	1	1	1	0	0	1	1	0	0	1	0	0	0	1	1	1
1	1	1	1	1	1	1	1	1	0	1	1	0	1	1	1	1	1
1	1	1	1	1	0	0	1	1	0	0	1	0	0	0	1	1	1
0	1	1	1	1	0	0	1	1	0	0	1	0	0	0	1	1	1
1	1	1	1	1	1	1	1	1	1	1	1	1	1	1	1	1	1
0	1	1	1	1	0	0	1	1	0	0	1	0	0	0	1	1	1
0	1	1	1	1	0	0	1	1	0	0	1	0	0	0	1	1	1
0	1	1	1	1	0	0	1	1	0	0	1	0	0	0	1	1	1
0	1	1	1	1	0	0	1	1	0	0	1	0	0	0	1	1	1
1	1	1	1	1	1	1	1	1	0	1	1	1	1	1	1	1	1
1	1	1	1	1	0	1	1	1	0	1	1	0	0	0	1	1	1
1	1	1	1	1	0	1	1	1	0	1	1	0	0	0	1	1	1
1	1	1	1	1	1	1	1	1	0	1	1	1	1	1	1	1	1
0	1	1	1	1	0	0	1	1	0	0	1	0	0	0	1	1	1
1	1	1	1	1	0	1	1	1	1	1	1	0	0	0	1	1	1
1	1	1	1	1	0	1	1	1	0	1	1	0	0	0	1	1	1
1	1	1	1	1	0	1	1	1	0	1	1	0	0	0	1	1	1
1	1	1	1	1	0	1	1	1	0	1	1	0	0	0	1	1	1
1	1	1	1	1	0	1	1	1	0	1	1	0	0	0	1	1	1
0	1	1	1	1	0	0	1	1	0	0	1	0	0	0	1	1	1
1	1	1	1	1	1	1	1	1	0	1	1	1	1	1	1	1	1
1	1	1	1	1	0	1	1	1	0	1	1	0	0	0	1	1	1
1	1	1	1	1	1	1	1	1	0	1	1	1	1	1	1	1	1
1	1	1	1	1	1	1	1	1	0	1	1	1	1	1	1	1	1
1	1	1	1	1	1	1	1	1	0	1	1	1	1	1	1	1	1
1	1	1	1	1	1	1	1	1	1	1	1	1	1	1	1	1	1
1	1	1	1	1	1	1	1	1	1	1	1	0	1	1	1	1	1
1	1	1	1	1	1	1	1	1	0	1	1	0	1	1	1	1	1
1	1	1	1	1	0	1	1	1	0	1	1	0	0	0	1	1	1
1	1	1	1	1	0	1	1	1	0	1	1	0	0	0	1	1	1
1	1	1	1	1	0	1	1	1	0	1	1	0	0	0	1	1	1
1	1	1	1	1	0	1	1	1	0	1	1	0	0	0	1	1	1

1	1	1	1	1	0	1	1	1	0	1	1	0	0	0	1	1	1
1	1	1	1	1	0	1	1	1	0	1	1	0	0	0	1	1	1
1	1	1	1	1	0	1	1	1	0	1	1	0	0	0	1	1	1
1	1	1	1	1	0	1	1	1	0	1	1	0	0	0	1	1	1
1	1	1	1	1	0	1	1	1	0	1	1	0	0	0	1	1	1
1	1	1	1	1	0	1	1	1	0	1	1	0	0	0	1	1	1
1	1	1	1	1	0	1	1	1	0	1	1	0	0	0	1	1	1
1	1	1	1	1	0	1	1	1	0	1	1	0	0	0	1	1	1
1	1	1	1	1	0	1	1	1	0	1	1	0	0	0	1	1	1
1	1	1	1	1	0	1	1	1	0	1	1	0	0	0	1	1	1
1	1	1	1	1	0	1	1	1	0	1	1	0	0	0	1	1	1
1	1	1	1	1	0	1	1	1	0	1	1	0	0	0	1	1	1
0	1	1	1	1	0	0	1	1	0	0	1	0	0	0	1	1	1
0	1	1	1	1	0	0	1	1	0	0	1	0	0	0	1	1	1
0	1	1	1	1	0	0	1	1	0	0	1	0	0	0	1	1	1
0	1	1	1	1	0	0	1	1	0	0	1	0	0	0	1	1	1
0	1	1	1	1	0	0	1	1	0	0	1	0	0	0	1	1	1
0	1	1	1	1	0	0	1	1	0	0	1	0	0	0	1	1	1
1	1	1	1	1	0	1	1	1	0	1	1	1	0	0	1	1	1
1	1	1	1	1	0	1	1	1	0	1	1	1	0	0	1	1	1
1	1	1	1	1	0	1	1	1	0	1	1	1	0	0	1	1	1
1	1	1	1	1	0	1	1	1	0	1	1	1	0	0	1	1	1
1	1	1	1	1	0	1	1	1	0	1	1	1	0	0	1	1	1
1	1	1	1	1	0	1	1	1	0	1	1	0	0	0	1	1	1
1	1	1	1	1	1	1	1	1	0	1	1	1	1	1	1	1	1
1	1	1	1	1	1	1	1	1	0	1	1	1	1	1	1	1	1
0	1	1	1	1	0	0	1	1	0	0	1	0	0	0	1	1	1
0	1	1	1	1	0	0	1	1	0	0	1	0	0	0	1	1	1
0	1	1	1	1	0	0	1	1	0	0	1	0	0	0	1	1	1
0	1	1	1	1	0	0	1	1	0	0	1	0	0	0	1	1	1
0	1	1	1	1	0	0	1	1	0	0	1	0	0	0	1	1	1
1	1	1	1	1	0	1	1	1	0	1	1	1	0	0	1	1	1
1	1	1	1	1	0	1	1	1	0	1	1	1	0	0	1	1	1
0	1	1	1	1	0	0	1	1	0	0	1	0	0	0	1	1	1
0	1	1	1	1	0	0	1	1	0	0	1	0	0	0	1	1	1
1	1	1	1	1	0	1	1	1	0	1	1	1	0	0	1	1	1
1	1	1	1	1	0	1	1	1	0	1	1	1	0	0	1	1	1
1	1	1	1	1	0	1	1	1	0	1	1	1	0	0	1	1	1
1	1	1	1	1	0	1	1	1	1	1	1	1	0	0	1	1	1
1	1	1	1	1	0	1	1	1	0	1	1	1	0	0	1	1	1
1	1	1	1	1	0	1	1	1	0	1	1	1	0	0	1	1	1
0	1	1	1	1	0	0	1	1	0	0	1	0	0	0	1	1	1
0	1	1	1	1	0	0	1	1	0	0	1	0	0	0	1	1	1

0	1	1	1	1	0	0	1	1	0	0	1	0	0	0	1	1	1
0	1	1	1	1	0	0	1	1	0	0	1	0	0	0	1	1	1
0	1	1	1	1	0	0	1	1	0	0	1	0	0	0	1	1	1
0	1	1	1	1	0	0	1	1	0	0	1	0	0	0	1	1	1
1	1	1	1	1	0	0	0	1	0	0	1	0	0	0	1	1	1
1	1	1	1	1	0	0	0	1	0	0	1	0	0	0	1	1	1
0	1	1	1	1	0	0	1	1	0	0	1	0	0	0	1	1	1
1	1	0	1	1	0	0	1	0	0	0	0	0	0	0	0	1	1
1	1	1	1	1	0	1	1	1	0	1	1	0	0	0	1	1	1
1	1	1	1	1	0	1	1	1	0	1	1	0	0	0	1	1	1
1	1	1	1	1	0	0	0	1	0	0	1	0	0	0	1	1	1
1	1	1	1	1	1	1	1	1	1	1	1	1	1	1	1	1	1
1	1	1	1	1	0	0	0	1	0	0	1	0	0	0	1	1	1
1	1	1	1	1	0	0	0	1	0	0	1	0	0	0	1	1	1
1	1	1	1	1	0	1	1	1	0	1	1	1	1	1	1	1	1
1	1	1	1	1	0	0	0	1	0	0	1	0	0	0	1	1	1
1	1	1	1	1	0	0	1	1	1	0	1	0	0	0	0	1	1
0	1	1	1	1	0	0	1	1	0	0	1	0	0	0	1	1	1
0	1	1	1	1	0	0	1	1	0	0	1	0	0	0	1	1	1
1	1	1	1	1	0	0	1	1	0	0	1	0	0	0	0	1	1
1	1	1	1	1	0	0	1	1	0	0	1	0	0	0	0	1	1
0	1	1	1	1	0	0	1	1	0	0	1	0	0	0	1	1	1
0	1	1	1	1	0	0	1	1	0	0	1	0	0	0	1	1	1
1	1	1	1	1	0	0	1	1	0	0	1	0	0	0	0	1	1

sseG	sseI/srfH	sseJ	sseK1	sseK2	sseL	sspH2	pipB	pipB2	sipA/sspA	sipB/sspB	sipC/sspC	sipD	avrA	sopA	pB/sig	sopD	sopD2
TTSS-1 translocated effectors																	
sseG	sseI/srfH	sseJ	sseK1	sseK2	sseL	sspH2	pipB	pipB2	sipA/sspA	sipB/sspB	sipC/sspC	sipD	avrA	sopA	sopB/	sopD	sopD2
1	1	1	1	1	1	1	1	1	1	1	1	1	1	1	1	1	1
1	0	1	1	1	0	0	1	0	1	1	1	1	1	1	1	1	0
1	0	1	1	1	0	0	1	0	1	1	1	1	1	1	1	1	0
1	0	1	1	1	0	0	1	0	1	1	1	1	1	1	1	1	0
1	1	1	1	0	1	0	1	1	1	1	1	1	1	1	1	1	1
1	1	1	1	1	1	1	1	1	1	1	1	1	1	1	1	1	1
1	0	1	1	1	0	0	1	0	1	1	1	1	1	1	1	1	0
1	1	1	1	0	1	0	1	1	1	1	1	1	1	1	1	1	1
1	0	1	0	1	1	0	1	0	1	1	1	1	0	1	1	1	1
1	0	1	1	1	0	0	1	0	1	1	1	1	1	1	1	1	0
1	1	1	1	1	1	1	1	1	1	1	1	1	1	1	1	1	1
1	0	1	1	1	0	0	1	0	1	1	1	1	1	1	1	1	0
1	0	1	1	1	0	0	1	0	1	1	1	1	1	1	1	1	0
1	0	1	1	1	0	0	1	0	1	1	1	1	1	1	1	1	0
1	0	1	1	1	0	0	1	0	1	1	1	1	1	1	1	1	0
1	1	1	1	1	1	1	1	1	1	1	1	1	1	1	1	1	1
1	1	1	1	0	1	1	1	1	1	1	1	1	1	1	1	1	1
1	1	1	1	0	1	1	1	1	1	1	1	1	1	1	1	1	1
1	1	1	1	1	1	1	1	1	1	1	1	1	1	1	1	1	1
1	0	1	1	1	0	0	1	0	1	1	1	1	1	1	1	1	0
1	1	1	1	0	1	1	1	1	1	1	1	1	1	1	1	1	1
1	1	1	1	0	1	1	1	1	1	1	1	1	1	1	1	1	1
1	1	1	1	0	1	1	1	1	1	1	1	1	1	1	1	1	1
1	1	1	1	0	1	1	1	1	1	1	1	1	1	1	1	1	1
1	1	1	1	0	1	1	1	1	1	1	1	1	1	1	1	1	1
1	0	1	1	1	0	0	1	0	1	1	1	1	1	1	1	1	0
1	1	1	1	1	1	1	1	1	1	1	1	1	1	1	1	1	1
1	1	1	1	0	1	1	1	1	1	1	1	1	1	1	1	1	1
1	1	1	1	1	1	1	1	1	1	1	1	1	1	1	1	1	1
1	1	1	1	1	1	1	1	1	1	1	1	1	1	1	1	1	1
1	1	1	1	1	1	1	1	1	1	1	1	1	1	1	1	1	1
1	1	1	1	1	1	1	1	1	1	1	1	1	1	1	1	1	1
1	1	1	1	0	1	0	1	1	1	1	1	1	1	1	1	1	1
1	1	1	1	0	1	0	1	1	1	1	1	1	1	1	1	1	1
1	1	1	1	0	1	1	1	1	1	1	1	1	1	1	1	1	1
1	1	1	1	0	1	1	1	1	1	1	1	1	1	1	1	1	1
1	0	1	1	0	1	1	1	1	1	1	1	1	1	1	1	1	1
1	1	1	1	0	1	1	1	1	1	1	1	1	1	1	1	1	1

1	0	1	1	0	1	1	1	1	1	1	1	1	1	1	1	1	1
1	1	1	1	0	1	1	1	1	1	1	1	1	1	1	1	1	1
1	1	1	1	0	1	1	1	1	1	1	1	1	1	1	1	1	1
1	0	1	1	0	1	1	1	1	1	1	1	1	1	1	1	1	1
1	1	1	1	0	1	1	1	1	1	1	1	1	1	1	1	1	1
1	0	1	1	0	1	1	1	1	1	1	1	1	1	1	1	1	1
1	0	1	1	0	1	1	1	1	1	1	1	1	1	1	1	1	1
1	0	1	1	0	1	1	1	1	1	1	1	1	1	1	1	1	0
1	0	1	1	0	1	1	1	1	1	1	1	1	1	1	1	1	1
1	1	1	1	0	1	1	1	1	1	1	1	1	1	1	1	1	1
1	1	1	1	0	1	1	1	1	1	1	1	1	1	1	1	1	1
1	1	1	1	0	1	1	1	1	1	1	1	1	1	1	1	1	1
1	0	1	1	1	0	0	1	0	1	1	1	1	1	1	1	1	0
1	0	1	1	1	0	0	1	0	1	1	1	1	1	1	1	1	0
1	0	1	1	1	0	0	1	0	1	1	1	1	1	1	1	1	0
1	0	1	1	1	0	0	1	0	1	1	1	1	1	1	1	1	0
1	0	1	1	1	0	0	1	0	1	1	1	1	1	1	1	1	0
1	0	1	1	1	0	0	1	0	1	1	1	1	1	1	1	1	0
1	1	1	1	1	1	1	1	1	1	1	1	1	1	1	1	1	1
1	1	1	1	1	1	1	1	1	1	1	1	1	1	1	1	1	1
1	1	1	1	1	1	1	1	1	1	1	1	1	1	1	1	1	1
1	1	1	1	1	1	1	1	1	1	1	1	1	1	1	1	1	1
1	1	1	1	1	1	1	1	1	1	1	1	1	1	1	1	1	1
1	1	1	1	0	1	1	1	1	1	1	1	1	1	1	1	1	1
1	1	1	1	1	1	1	1	1	1	1	1	1	1	1	1	1	1
1	1	1	1	1	1	1	1	1	1	1	1	1	1	1	1	1	1
1	0	1	1	1	0	0	1	0	1	1	1	1	1	1	1	1	0
1	0	1	1	1	0	0	1	0	1	1	1	1	1	1	1	1	0
1	0	1	1	1	0	0	1	0	1	1	1	1	1	1	1	1	0
1	0	1	1	1	0	0	1	0	1	1	1	1	1	1	1	1	0
1	0	1	1	1	0	0	1	0	1	1	1	1	1	1	1	1	0
1	1	1	1	1	1	1	1	1	1	1	1	1	1	1	1	1	1
1	1	1	1	1	1	1	1	1	1	1	1	1	1	1	1	1	1
1	0	1	1	1	0	0	1	0	1	1	1	1	1	1	1	1	0
1	0	1	1	1	0	0	1	0	1	1	1	1	1	1	1	1	0
1	1	1	1	1	1	1	1	1	1	1	1	1	1	1	1	1	1
1	1	1	1	1	1	1	1	1	1	1	1	1	1	1	1	1	1
1	1	1	1	1	1	1	1	1	1	1	1	1	1	1	1	1	1
1	1	1	1	1	1	1	1	1	1	1	1	1	1	1	1	1	1
1	1	1	1	1	1	1	1	1	1	1	1	1	1	1	1	1	1
1	1	1	1	1	1	1	1	1	1	1	1	1	1	1	1	1	1
1	0	1	1	1	0	0	1	0	1	1	1	1	1	1	1	1	0
1	0	1	1	0	1	0	1	0	1	1	1	1	1	1	1	1	0

1	0	1	1	0	1	0	1	0	1	1	1	1	1	1	1	1	0
1	0	1	1	0	1	0	1	0	1	1	1	1	1	1	1	1	0
1	0	1	1	0	1	0	1	0	1	1	1	1	1	1	1	1	0
1	0	1	1	0	1	0	1	0	1	1	1	1	1	1	1	1	0
1	0	1	0	1	1	0	1	0	1	1	1	1	0	1	1	1	1
1	0	1	0	1	1	0	1	0	1	1	1	1	0	1	1	1	1
1	0	1	1	0	0	0	1	0	1	1	1	1	1	1	1	1	0
1	0	0	1	1	0	0	1	0	1	1	1	1	1	0	1	0	1
1	1	1	1	0	1	1	1	1	1	1	1	1	1	1	1	1	1
1	1	1	1	0	1	1	1	1	1	1	1	1	1	1	1	1	1
1	0	1	0	1	1	0	1	0	1	1	1	1	0	1	1	1	1
1	1	1	1	1	1	1	1	1	1	1	1	1	1	1	1	1	1
1	0	1	0	1	1	0	1	0	1	1	1	1	0	1	1	1	1
1	0	1	0	1	1	0	1	0	1	1	1	1	0	1	1	1	1
1	1	1	1	1	1	1	1	1	1	1	1	1	1	1	1	1	1
1	0	1	0	1	1	0	1	0	1	1	1	1	0	1	1	1	1
1	0	1	1	1	1	0	1	0	1	1	1	1	1	1	1	1	0
1	0	1	1	1	0	0	1	0	1	1	1	1	1	1	1	1	0
1	0	1	1	1	1	0	1	0	1	1	1	1	1	1	1	1	0
1	0	1	1	1	1	0	1	0	1	1	1	1	1	1	1	1	0
1	0	1	1	1	1	0	1	0	1	1	1	1	1	1	1	1	0
1	0	1	1	1	0	0	1	0	1	1	1	1	1	1	1	1	0
1	0	1	1	1	0	0	1	0	1	1	1	1	1	1	1	1	0
1	0	1	1	1	1	0	1	0	1	1	1	1	1	1	1	1	0

sopE2	sptP	invA	invB	invC	invE	invF	invG	invH	invI	invJ	orgA	orgB	orgC	prgH	prgI	prgJ	prgK	sicA	sicP	spaO	spaP	spaQ
TTSS (SPI-1 encode)																						
sopE2	*sptP*	*invA*	*invB*	*invC*	*invE*	*invF*	*invG*	*invH*	*invI*	*invJ*	*orgA*	*orgB*	*orgC*	*prgH*	*prgI*	*prgJ*	*prgK*	*sicA*	*sicP*	*spaO*	*spaP*	*spaQ*
1	1	1	1	1	1	1	1	1	1	1	1	1	1	1	1	1	1	1	1	1	1	1
1	1	1	1	1	1	1	1	1	1	1	1	1	1	1	1	1	1	1	1	1	1	1
1	1	1	1	1	1	1	1	1	1	1	1	1	1	1	1	1	1	1	1	1	1	1
1	1	1	1	1	1	1	1	1	1	1	1	1	1	1	1	1	1	1	1	1	1	1
1	1	1	1	1	1	1	1	1	1	1	1	1	1	1	1	1	1	1	1	1	1	1
1	1	1	1	1	1	1	1	1	1	1	1	1	1	1	1	1	1	1	1	1	1	1
1	1	1	1	1	1	1	1	1	1	1	1	1	1	1	1	1	1	1	1	1	1	1
1	1	1	1	1	1	1	1	1	1	1	1	1	1	1	1	1	1	1	1	1	1	1
1	1	1	1	1	1	1	1	1	1	1	1	1	1	1	1	1	1	1	1	1	1	1
1	1	1	1	1	1	1	1	1	1	1	1	1	1	1	1	1	1	1	1	1	1	1
1	1	1	1	1	1	1	1	1	1	1	1	1	1	1	1	1	1	1	1	1	1	1
1	1	1	1	1	1	1	1	1	1	1	1	1	1	1	1	1	1	1	1	1	1	1
1	1	1	1	1	1	1	1	1	1	1	1	1	1	1	1	1	1	1	1	1	1	1
1	1	1	1	1	1	1	1	1	1	1	1	1	1	1	1	1	1	1	1	1	1	1
1	1	1	1	1	1	1	1	1	1	1	1	1	1	1	1	1	1	1	1	1	1	1
1	1	1	1	1	1	1	1	1	1	1	1	1	1	1	1	1	1	1	1	1	1	1
1	1	1	1	1	1	1	1	1	1	1	1	1	1	1	1	1	1	1	1	1	1	1
1	1	1	1	1	1	1	1	1	1	1	1	1	1	1	1	1	1	1	1	1	1	1
1	1	1	1	1	1	1	1	1	1	1	1	1	1	1	1	1	1	1	1	1	1	1
1	1	1	1	1	1	1	1	1	1	1	1	1	1	1	1	1	1	1	1	1	1	1
1	1	1	1	1	1	1	1	1	1	1	1	1	1	1	1	1	1	1	1	1	1	1
1	1	1	1	1	1	1	1	1	1	1	1	1	1	1	1	1	1	1	1	1	1	1
1	1	1	1	1	1	1	1	1	1	1	1	1	1	1	1	1	1	1	1	1	1	1
1	1	1	1	1	1	1	1	1	1	1	1	1	1	1	1	1	1	1	1	1	1	1
1	1	1	1	1	1	1	1	1	1	1	1	1	1	1	1	1	1	1	1	1	1	1
1	1	1	1	1	1	1	1	1	1	1	1	1	1	1	1	1	1	1	1	1	1	1
1	1	1	1	1	1	1	1	1	1	1	1	1	1	1	1	1	1	1	1	1	1	1
1	1	1	1	1	1	1	1	1	1	1	1	1	1	1	1	1	1	1	1	1	1	1
1	1	1	1	1	1	1	1	1	1	1	1	1	1	1	1	1	1	1	1	1	1	1
1	1	1	1	1	1	1	1	1	1	1	1	1	1	1	1	1	1	1	1	1	1	1
1	1	1	1	1	1	1	1	1	1	1	1	1	1	1	1	1	1	1	1	1	1	1
1	1	1	1	1	1	1	1	1	1	1	1	1	1	1	1	1	1	1	1	1	1	1
1	1	1	1	1	1	1	1	1	1	1	1	1	1	1	1	1	1	1	1	1	1	1
1	1	1	1	1	1	1	1	1	1	1	1	1	1	1	1	1	1	1	1	1	1	1
1	1	1	1	1	1	1	1	1	1	1	1	1	1	1	1	1	1	1	1	1	1	1
1	1	1	1	1	1	1	1	1	1	1	1	1	1	1	1	1	1	1	1	1	1	1
1	1	1	1	1	1	1	1	1	1	1	1	1	1	1	1	1	1	1	1	1	1	1
1	1	1	1	1	1	1	1	1	1	1	1	1	1	1	1	1	1	1	1	1	1	1
1	1	1	1	1	1	1	1	1	1	1	1	1	1	1	1	1	1	1	1	1	1	1
1	1	1	1	1	1	1	1	1	1	1	1	1	1	1	1	1	1	1	1	1	1	1
1	1	1	1	1	1	1	1	1	1	1	1	1	1	1	1	1	1	1	1	1	1	1

1	1	1	1	1	1	1	1	1	1	1	1	1	1	1	1	1	1	1	1	1	1	1
1	1	1	1	1	1	1	1	1	1	1	1	1	1	1	1	1	1	1	1	1	1	1
1	1	1	1	1	1	1	1	1	1	1	1	1	1	1	1	1	1	1	1	1	1	1
1	1	1	1	1	1	1	1	1	1	1	1	1	1	1	1	1	1	1	1	1	1	1
1	1	1	1	1	1	1	1	1	1	1	1	1	1	1	1	1	1	1	1	1	1	1
1	1	1	1	1	1	1	1	1	1	1	1	1	1	1	1	1	1	1	1	1	1	1
1	1	1	1	1	1	1	1	1	1	1	1	1	1	1	1	1	1	1	1	1	1	1
1	1	1	1	1	1	1	1	1	1	1	1	1	1	1	1	1	1	1	1	1	1	1
1	1	1	1	1	1	1	1	1	1	1	1	1	1	1	1	1	1	1	1	1	1	1
1	1	1	1	1	1	1	1	1	1	1	1	1	1	1	1	1	1	1	1	1	1	1
1	1	1	1	1	1	1	1	1	1	1	1	1	1	1	1	1	1	1	1	1	1	1
1	1	1	1	1	1	1	1	1	1	1	1	1	1	1	1	1	1	1	1	1	1	1
1	1	1	1	1	1	1	1	1	1	1	1	1	1	1	1	1	1	1	1	1	1	1
1	1	1	1	1	1	1	1	1	1	1	1	1	1	1	1	1	1	1	1	1	1	1
1	1	1	1	1	1	1	1	1	1	1	1	1	1	1	1	1	1	1	1	1	1	1
1	1	1	1	1	1	1	1	1	1	1	1	1	1	1	1	1	1	1	1	1	1	1
1	1	1	1	1	1	1	1	1	1	1	1	1	1	1	1	1	1	1	1	1	1	1
1	1	1	1	1	1	1	1	1	1	1	1	1	1	1	1	1	1	1	1	1	1	1
1	1	1	1	1	1	1	1	1	1	1	1	1	1	1	1	1	1	1	1	1	1	1
1	1	1	1	1	1	1	1	1	1	1	1	1	1	1	1	1	1	1	1	1	1	1
1	1	1	1	1	1	1	1	1	1	1	1	1	1	1	1	1	1	1	1	1	1	1
1	1	1	1	1	1	1	1	1	1	1	1	1	1	1	1	1	1	1	1	1	1	1
1	1	1	1	1	1	1	1	1	1	1	1	1	1	1	1	1	1	1	1	1	1	1
1	1	1	1	1	1	1	1	1	1	1	1	1	1	1	1	1	1	1	1	1	1	1
1	1	1	1	1	1	1	1	1	1	1	1	1	1	1	1	1	1	1	1	1	1	1
1	1	1	1	1	1	1	1	1	1	1	1	1	1	1	1	1	1	1	1	1	1	1
1	1	1	1	1	1	1	1	1	1	1	1	1	1	1	1	1	1	1	1	1	1	1
1	1	1	1	1	1	1	1	1	1	1	1	1	1	1	1	1	1	1	1	1	1	1
1	1	1	1	1	1	1	1	1	1	1	1	1	1	1	1	1	1	1	1	1	1	1
1	1	1	1	1	1	1	1	1	1	1	1	1	1	1	1	1	1	1	1	1	1	1
1	1	1	1	1	1	1	1	1	1	1	1	1	1	1	1	1	1	1	1	1	1	1
1	1	1	1	1	1	1	1	1	1	1	1	1	1	1	1	1	1	1	1	1	1	1
1	1	1	1	1	1	1	1	1	1	1	1	1	1	1	1	1	1	1	1	1	1	1
1	1	1	1	1	1	1	1	1	1	1	1	1	1	1	1	1	1	1	1	1	1	1
1	1	1	1	1	1	1	1	1	1	1	1	1	1	1	1	1	1	1	1	1	1	1
1	1	1	1	1	1	1	1	1	1	1	1	1	1	1	1	1	1	1	1	1	1	1
1	1	1	1	1	1	1	1	1	1	1	1	1	1	1	1	1	1	1	1	1	1	1
1	1	1	1	1	1	1	1	1	1	1	1	1	1	1	1	1	1	1	1	1	1	1
1	1	1	1	1	1	1	1	1	1	1	1	1	1	1	1	1	1	1	1	1	1	1
1	1	1	1	1	1	1	1	1	1	1	1	1	1	1	1	1	1	1	1	1	1	1
1	1	1	1	1	1	1	1	1	1	1	1	1	1	1	1	1	1	1	1	1	1	1
1	1	1	1	1	1	1	1	1	1	1	1	1	1	1	1	1	1	1	1	1	1	1
1	1	1	1	1	1	1	1	1	1	1	1	1	1	1	1	1	1	1	1	1	1	1

1	1	1	1	1	1	1	1	1	1	1	1	1	1	1	1	1	1	1	1	1	1	1
1	1	1	1	1	1	1	1	1	1	1	1	1	1	1	1	1	1	1	1	1	1	1
1	1	1	1	1	1	1	1	1	1	1	1	1	1	1	1	1	1	1	1	1	1	1
1	1	1	1	1	1	1	1	1	1	1	1	1	1	1	1	1	1	1	1	1	1	1
1	1	1	1	1	1	1	1	1	1	1	1	1	1	1	1	1	1	1	1	1	1	1
1	1	1	1	1	1	1	1	1	1	1	1	1	1	1	1	1	1	1	1	1	1	1
1	1	1	1	1	1	1	1	1	1	1	1	1	1	1	1	1	1	1	1	1	1	1
1	0	1	1	1	0	1	1	1	1	1	1	1	1	0	1	1	1	1	1	1	1	1
1	1	1	1	1	1	1	1	1	1	1	1	1	1	1	1	1	1	1	1	1	1	1
1	1	1	1	1	1	1	1	1	1	1	1	1	1	1	1	1	1	1	1	1	1	1
1	1	1	1	1	1	1	1	1	1	1	1	1	1	1	1	1	1	1	1	1	1	1
1	1	1	1	1	1	1	1	1	1	1	1	1	1	1	1	1	1	1	1	1	1	1
1	1	1	1	1	1	1	1	1	1	1	1	1	1	1	1	1	1	1	1	1	1	1
1	1	1	1	1	1	1	1	1	1	1	1	1	1	1	1	1	1	1	1	1	1	1
1	1	1	1	1	1	1	1	1	1	1	1	1	1	1	1	1	1	1	1	1	1	1
1	1	1	1	1	1	1	1	1	1	1	1	1	1	1	1	1	1	1	1	1	1	1
1	1	1	1	1	1	1	1	1	1	1	1	1	1	1	1	1	1	1	1	1	1	1
1	1	1	1	1	1	1	1	1	1	1	1	1	1	1	1	1	1	1	1	1	1	1
1	1	1	1	1	1	1	1	1	1	1	1	1	1	1	1	1	1	1	1	1	1	1
1	1	1	1	1	1	1	1	1	1	1	1	1	1	1	1	1	1	1	1	1	1	1
1	1	1	1	1	1	1	1	1	1	1	1	1	1	1	1	1	1	1	1	1	1	1
1	1	1	1	1	1	1	1	1	1	1	1	1	1	1	1	1	1	1	1	1	1	1
1	1	1	1	1	1	1	1	1	1	1	1	1	1	1	1	1	1	1	1	1	1	1
1	1	1	1	1	1	1	1	1	1	1	1	1	1	1	1	1	1	1	1	1	1	1

spaR	spaS	slrP	:piC/ssaE	ssaC	ssaD	ssaE	ssaG	ssaH	ssaI	ssaJ	ssaK	ssaL	ssaM	ssaN	ssaO	ssaP	ssaQ
		TTSS e(SPI-2 encode)															
spaR	*spaS*	*slrP*	*spiC/ssa*	*ssaC*	*ssaD*	*ssaE*	*ssaG*	*ssaH*	*ssaI*	*ssaJ*	*ssaK*	*ssaL*	*ssaM*	*ssaN*	*ssaO*	*ssaP*	*ssaQ*
1	1	1	1	1	1	1	1	1	1	1	1	1	1	1	1	1	1
1	1	0	1	1	1	1	1	1	1	1	1	1	1	1	1	1	1
1	1	0	1	1	1	1	1	1	1	1	1	1	1	1	1	1	1
1	1	0	1	1	1	1	1	1	1	1	1	1	1	1	1	1	1
1	1	1	1	1	1	1	1	1	1	1	1	1	1	1	1	1	1
1	1	1	1	1	1	1	1	1	1	1	1	1	1	1	1	1	1
1	1	0	1	1	1	1	1	1	1	1	1	1	1	1	1	1	1
1	1	1	1	1	1	1	1	1	1	1	1	1	1	1	1	1	1
1	1	1	1	1	1	1	1	1	1	1	1	1	1	1	1	1	1
1	1	0	1	1	1	1	1	1	1	1	1	1	1	1	1	1	1
1	1	1	1	1	1	1	1	1	1	1	1	1	1	1	1	1	1
1	1	0	1	1	1	1	1	1	1	1	1	1	1	1	1	1	1
1	1	0	1	1	1	1	1	1	1	1	1	1	1	1	1	1	1
1	1	0	1	1	1	1	1	1	1	1	1	1	1	1	1	1	1
1	1	0	1	1	1	1	1	1	1	1	1	1	1	1	1	1	1
1	1	1	1	1	1	1	1	1	1	1	1	1	1	1	1	1	1
1	1	1	1	1	1	1	1	1	1	1	1	1	1	1	1	1	1
1	1	1	1	1	1	1	1	1	1	1	1	1	1	1	1	1	1
1	1	1	1	1	1	1	1	1	1	1	1	1	1	1	1	1	1
1	1	0	1	1	1	1	1	1	1	1	1	1	1	1	1	1	1
1	1	1	1	1	1	1	1	1	1	1	1	1	1	1	1	1	1
1	1	1	1	1	1	1	1	1	1	1	1	1	1	1	1	1	1
1	1	1	1	1	1	1	1	1	1	1	1	1	1	1	1	1	1
1	1	1	1	1	1	1	1	1	1	1	1	1	1	1	1	1	1
1	1	1	1	1	1	1	1	1	1	1	1	1	1	1	1	1	1
1	1	0	1	1	1	1	1	1	1	1	1	1	1	1	1	1	1
1	1	1	1	1	1	1	1	1	1	1	1	1	1	1	1	1	1
1	1	1	1	1	1	1	1	1	1	1	1	1	1	1	1	1	1
1	1	1	1	1	1	1	1	1	1	1	1	1	1	1	1	1	1
1	1	1	1	1	1	1	1	1	1	1	1	1	1	1	1	1	1
1	1	1	1	1	1	1	1	1	1	1	1	1	1	1	1	1	1
1	1	1	1	1	1	1	1	1	1	1	1	1	1	1	1	1	1
1	1	1	1	1	1	1	1	1	1	1	1	1	1	1	1	1	1
1	1	1	1	1	1	1	1	1	1	1	1	1	1	1	1	1	1
1	1	1	1	1	1	1	1	1	1	1	1	1	1	1	1	1	1
1	1	1	1	1	1	1	1	1	1	1	1	1	1	1	1	1	1
1	1	1	1	1	1	1	1	1	1	1	1	1	1	1	1	1	1
1	1	1	1	1	1	1	1	1	1	1	1	1	1	1	1	1	1
1	1	1	1	1	1	1	1	1	1	1	1	1	1	1	1	1	1

1	1	1	1	1	1	1	1	1	1	1	1	1	1	1	1	1	1
1	1	1	1	1	1	1	1	1	1	1	1	1	1	1	1	1	1
1	1	1	1	1	1	1	1	1	1	1	1	1	1	1	1	1	1
1	1	1	1	1	1	1	1	1	1	1	1	1	1	1	1	1	1
1	1	1	1	1	1	1	1	1	1	1	1	1	1	1	1	1	1
1	1	1	1	1	1	1	1	1	1	1	1	1	1	1	1	1	1
1	1	1	1	1	1	1	1	1	1	1	1	1	1	1	1	1	1
1	1	1	1	1	1	1	1	1	1	1	1	1	1	1	1	1	1
1	1	1	1	1	1	1	1	1	1	1	1	1	1	1	1	1	1
1	1	1	1	1	1	1	1	1	1	1	1	1	1	1	1	1	1
1	1	1	1	1	1	1	1	1	1	1	1	1	1	1	1	1	1
1	1	1	1	1	1	1	1	1	1	1	1	1	1	1	1	1	1
1	1	0	1	1	1	1	1	1	1	1	1	1	1	1	1	1	1
1	1	0	1	1	1	1	1	1	1	1	1	1	1	1	1	1	1
1	1	0	1	1	1	1	1	1	1	1	1	1	1	1	1	1	1
1	1	0	1	1	1	1	1	1	1	1	1	1	1	1	1	1	1
1	1	0	1	1	1	1	1	1	1	1	1	1	1	1	1	1	1
1	1	0	1	1	1	1	1	1	1	1	1	1	1	1	1	1	1
1	1	1	1	1	1	1	1	1	1	1	1	1	1	1	1	1	1
1	1	1	1	1	1	1	1	1	1	1	1	1	1	1	1	1	1
1	1	1	1	1	1	1	1	1	1	1	1	1	1	1	1	1	1
1	1	1	1	1	1	1	1	1	1	1	1	1	1	1	1	1	1
1	1	1	1	1	1	1	1	1	1	1	1	1	1	1	1	1	1
1	1	1	1	1	1	1	1	1	1	1	1	1	1	1	1	1	1
1	1	1	1	1	1	1	1	1	1	1	1	1	1	1	1	1	1
1	1	1	1	1	1	1	1	1	1	1	1	1	1	1	1	1	1
1	1	0	1	1	1	1	1	1	1	1	1	1	1	1	1	1	1
1	1	0	1	1	1	1	1	1	1	1	1	1	1	1	1	1	1
1	1	0	1	1	1	1	1	1	1	1	1	1	1	1	1	1	1
1	1	0	1	1	1	1	1	1	1	1	1	1	1	1	1	1	1
1	1	0	1	1	1	1	1	1	1	1	1	1	1	1	1	1	1
1	1	1	1	1	1	1	1	1	1	1	1	1	1	1	1	1	1
1	1	1	1	1	1	1	1	1	1	1	1	1	1	1	1	1	1
1	1	0	1	1	1	1	1	1	1	1	1	1	1	1	1	1	1
1	1	0	1	1	1	1	1	1	1	1	1	1	1	1	1	1	1
1	1	1	1	1	1	1	1	1	1	1	1	1	1	1	1	1	1
1	1	1	1	1	1	1	1	1	1	1	1	1	1	1	1	1	1
1	1	1	1	1	1	1	1	1	1	1	1	1	1	1	1	1	1
1	1	1	1	1	1	1	1	1	1	1	1	1	1	1	1	1	1
1	1	1	1	1	1	1	1	1	1	1	1	1	1	1	1	1	1
1	1	1	1	1	1	1	1	1	1	1	1	1	1	1	1	1	1
1	1	0	1	1	1	1	1	1	1	1	1	1	1	1	1	1	1
1	1	1	1	1	1	1	1	1	1	1	1	1	1	1	1	1	1

1	1	1	1	1	1	1	1	1	1	1	1	1	1	1	1	1	1
1	1	1	1	1	1	1	1	1	1	1	1	1	1	1	1	1	1
1	1	1	1	1	1	1	1	1	1	1	1	1	1	1	1	1	1
1	1	1	1	1	1	1	1	1	1	1	1	1	1	1	1	1	1
1	1	1	1	1	1	1	1	1	1	1	1	1	1	1	1	1	1
1	1	1	1	1	1	1	1	1	1	1	1	1	1	1	1	1	1
1	1	0	1	1	1	1	1	1	1	1	1	1	1	1	1	1	1
1	1	0	1	1	1	1	1	1	1	1	1	1	1	1	1	1	1
1	1	1	1	1	1	1	1	1	1	1	1	1	1	1	1	1	1
1	1	1	1	1	1	1	1	1	1	1	1	1	1	1	1	1	1
1	1	1	1	1	1	1	1	1	1	1	1	1	1	1	1	1	1
1	1	1	1	1	1	1	1	1	1	1	1	1	1	1	1	1	1
1	1	1	1	1	1	1	1	1	1	1	1	1	1	1	1	1	1
1	1	1	1	1	1	1	1	1	1	1	1	1	1	1	1	1	1
1	1	1	1	1	1	1	1	1	1	1	1	1	1	1	1	1	1
1	1	1	1	1	1	1	1	1	1	1	1	1	1	1	1	1	1
1	1	1	1	1	1	1	1	1	1	1	1	1	1	1	1	1	1
1	1	0	1	1	1	1	1	1	1	1	1	1	1	1	1	1	1
1	1	0	1	1	1	1	1	1	1	1	1	1	1	1	1	1	1
1	1	1	1	1	1	1	1	1	1	1	1	1	1	1	1	1	1
1	1	1	1	1	1	1	1	1	1	1	1	1	1	1	1	1	1
1	1	0	1	1	1	1	1	1	1	1	1	1	1	1	1	1	1
1	1	0	1	1	1	1	1	1	1	1	1	1	1	1	1	1	1
1	1	1	1	1	1	1	1	1	1	1	1	1	1	1	1	1	1

ssaR	*ssaS*	*ssaT*	*ssaU*	*ssaV*	*sscA*	*sscB*	*sseA*	*sseB*	*sseC*	*sseD*	*sseE*	*papB*	*papI*	*grvA*	Ampicillin	Amoxicillir
**ssaR**	**ssaS**	**ssaT**	**ssaU**	**ssaV**	**sscA**	**sscB**	**sseA**	**sseB**	**sseC**	**sseD**	**sseE**	**papB**	**papI**	**grvA**	**Ampicillin**	**Amoxicillir**
1	1	1	1	1	1	1	1	1	1	1	1	0	0	1	> 512	8/4
1	1	1	1	1	1	1	1	1	1	1	1	0	0	0	1	1/0.5
1	1	1	1	1	1	1	1	1	1	1	1	0	0	0	1	1/0.5
1	1	1	1	1	1	1	1	1	1	1	1	0	0	0	1	0.5/0.25
1	1	1	1	1	1	1	1	1	1	1	1	0	0	0	> 512	8/4
1	1	1	1	1	1	1	1	1	1	1	1	0	0	1	> 512	16/8
1	1	1	1	1	1	1	1	1	1	1	1	0	0	0	1	1/0.5
1	1	1	1	1	1	1	1	1	1	1	1	0	0	0	> 512	16/8
1	1	1	1	1	1	1	1	1	1	1	1	0	0	0	1	0.5/0.25
1	1	1	1	1	1	1	1	1	1	1	1	0	0	0	1	0.5/0.25
1	1	1	1	1	1	1	1	1	1	1	1	0	0	1	1	1/0.5
1	1	1	1	1	1	1	1	1	1	1	1	0	0	0	512	32/16
1	1	1	1	1	1	1	1	1	1	1	1	0	0	0	> 512	8/4
1	1	1	1	1	1	1	1	1	1	1	1	0	0	0	> 512	8/4
1	1	1	1	1	1	1	1	1	1	1	1	0	0	0	> 512	8/4
1	1	1	1	1	1	1	1	1	1	1	1	0	0	1	> 512	16/8
1	1	1	1	1	1	1	1	1	1	1	1	0	0	0	> 512	8/4
1	1	1	1	1	1	1	1	1	1	1	1	0	0	0	1	0.5/0.25
1	1	1	1	1	1	1	1	1	1	1	1	0	0	1	> 512	16/8
1	1	1	1	1	1	1	1	1	1	1	1	0	0	0	> 512	16/8
1	1	1	1	1	1	1	1	1	1	1	1	0	0	0	> 512	16/8
1	1	1	1	1	1	1	1	1	1	1	1	0	0	0	> 512	8/4
1	1	1	1	1	1	1	1	1	1	1	1	0	0	0	> 512	16/8
1	1	1	1	1	1	1	1	1	1	1	1	0	0	0	> 512	8/4
1	1	1	1	1	1	1	1	1	1	1	1	0	0	0	> 512	8/4
1	1	1	1	1	1	1	1	1	1	1	1	0	0	0	> 512	16/8
1	1	1	1	1	1	1	1	1	1	1	1	0	0	1	> 512	8/4
1	1	1	1	1	1	1	1	1	1	1	1	0	0	0	> 512	8/4
1	1	1	1	1	1	1	1	1	1	1	1	0	0	1	512	8/4
1	1	1	1	1	1	1	1	1	1	1	1	0	0	1	256	8/4
1	1	1	1	1	1	1	1	1	1	1	1	0	0	1	512	8/4
1	1	1	1	1	1	1	1	1	1	1	1	0	0	1	512	8/4
1	1	1	1	1	1	1	1	1	1	1	1	0	0	0	512	8/4
1	1	1	1	1	1	1	1	1	1	1	1	0	0	0	4	8/4
1	1	1	1	1	1	1	1	1	1	1	1	0	0	1	> 512	16/8
1	1	1	1	1	1	1	1	1	1	1	1	0	0	1	> 512	8/4
1	1	1	1	1	1	1	1	1	1	1	1	0	0	1	0.5	1/0.5
1	1	1	1	1	1	1	1	1	1	1	1	0	0	1	1	2/1

1	1	1	1	1	1	1	1	1	1	1	1	0	0	1	1	0.5/0.25
1	1	1	1	1	1	1	1	1	1	1	1	0	0	1	1	0.5/0.25
1	1	1	1	1	1	1	1	1	1	1	1	0	0	1	1	0.5/0.25
1	1	1	1	1	1	1	1	1	1	1	1	0	0	1	1	0.5/0.25
1	1	1	1	1	1	1	1	1	1	1	1	0	0	1	> 512	8/4
1	1	1	1	1	1	1	1	1	1	1	1	0	0	1	1	0.5/0.25
1	1	1	1	1	1	1	1	1	1	1	1	0	0	1	1	0.5/0.25
1	1	1	1	1	1	1	1	1	1	1	1	0	0	1	1	0.5/0.25
1	1	1	1	1	1	1	1	1	1	1	1	0	0	1	0.5	0.5/0.25
1	1	1	1	1	1	1	1	1	1	1	1	0	0	1	1	2/1
1	1	1	1	1	1	1	1	1	1	1	1	0	0	1	1	1/0.5
1	1	1	1	1	1	1	1	1	1	1	1	0	0	1	1	0.5/0.25
1	1	1	1	1	1	1	1	1	1	1	1	0	0	0	> 512	8/4
1	1	1	1	1	1	1	1	1	1	1	1	0	0	0	> 512	8/4
1	1	1	1	1	1	1	1	1	1	1	1	0	0	0	> 512	8/4
1	1	1	1	1	1	1	1	1	1	1	1	0	0	0	> 512	8/4
1	1	1	1	1	1	1	1	1	1	1	1	0	0	0	> 512	8/4
1	1	1	1	1	1	1	1	1	1	1	1	0	0	0	> 512	8/4
1	1	1	1	1	1	1	1	1	1	1	1	0	0	0	128	8/4
1	1	1	1	1	1	1	1	1	1	1	1	0	0	0	128	16/8
1	1	1	1	1	1	1	1	1	1	1	1	0	0	0	256	16/8
1	1	1	1	1	1	1	1	1	1	1	1	0	0	0	512	16/8
1	1	1	1	1	1	1	1	1	1	1	1	0	0	0	128	16/8
1	1	1	1	1	1	1	1	1	1	1	1	0	0	1	1	2/1
1	1	1	1	1	1	1	1	1	1	1	1	0	0	1	256	4/2
1	1	1	1	1	1	1	1	1	1	1	1	0	0	1	256	4/2
1	1	1	1	1	1	1	1	1	1	1	1	0	0	0	> 512	8/4
1	1	1	1	1	1	1	1	1	1	1	1	0	0	0	> 512	8/4
1	1	1	1	1	1	1	1	1	1	1	1	0	0	0	> 512	8/4
1	1	1	1	1	1	1	1	1	1	1	1	0	0	0	> 512	8/4
1	1	1	1	1	1	1	1	1	1	1	1	0	0	0	> 512	8/4
1	1	1	1	1	1	1	1	1	1	1	1	0	0	0	128	16/8
1	1	1	1	1	1	1	1	1	1	1	1	0	0	0	128	16/8
1	1	1	1	1	1	1	1	1	1	1	1	0	0	0	> 512	8/4
1	1	1	1	1	1	1	1	1	1	1	1	0	0	0	256	16/8
1	1	1	1	1	1	1	1	1	1	1	1	0	0	0	128	32/16
1	1	1	1	1	1	1	1	1	1	1	1	0	0	0	256	32/16
1	1	1	1	1	1	1	1	1	1	1	1	0	0	0	256	32/16
1	1	1	1	1	1	1	1	1	1	1	1	0	0	0	512	16/8
1	1	1	1	1	1	1	1	1	1	1	1	0	0	0	512	32/16
1	1	1	1	1	1	1	1	1	1	1	1	0	0	0	512	16/8
1	1	1	1	1	1	1	1	1	1	1	1	0	0	0	256	16/8
1	1	1	1	1	1	1	1	1	1	1	1	0	0	0	1	0.5/0.25

1	1	1	1	1	1	1	1	1	1	1	1	0	0	0	1	0.5/0.25
1	1	1	1	1	1	1	1	1	1	1	1	0	0	0	1	1/0.5
1	1	1	1	1	1	1	1	1	1	1	1	0	0	0	1	0.5/0.25
1	1	1	1	1	1	1	1	1	1	1	1	0	0	0	1	0.5/0.25
1	1	1	1	1	1	1	1	1	1	1	1	0	0	0	256	16/8
1	1	1	1	1	1	1	1	1	1	1	1	0	0	0	256	16/8
1	1	1	1	1	1	1	1	1	1	1	1	0	0	0	> 512	8/4
1	1	0	0	1	1	1	1	0	1	1	1	0	0	0	128	16/8
1	1	1	1	1	1	1	1	1	1	1	1	0	0	0	256	16/8
1	1	1	1	1	1	1	1	1	1	1	1	0	0	0	256	16/8
1	1	1	1	1	1	1	1	1	1	1	1	0	0	0	256	16/8
1	1	1	1	1	1	1	1	1	1	1	1	0	0	1	1	1/0.5
1	1	1	1	1	1	1	1	1	1	1	1	0	0	0	32	8/4
1	1	1	1	1	1	1	1	1	1	1	1	0	0	0	64	16/8
1	1	1	1	1	1	1	1	1	1	1	1	0	0	1	> 512	16/8
1	1	1	1	1	1	1	1	1	1	1	1	0	0	0	128	16/8
1	1	1	1	1	1	1	1	1	1	1	1	0	0	0	2	1/0.5
1	1	1	1	1	1	1	1	1	1	1	1	0	0	0	> 512	4/2
1	1	1	1	1	1	1	1	1	1	1	1	0	0	0	≤0.25	0.5/0.25
1	1	1	1	1	1	1	1	1	1	1	1	0	0	0	1	1/0.5
1	1	1	1	1	1	1	1	1	1	1	1	0	0	0	1	1/0.5
1	1	1	1	1	1	1	1	1	1	1	1	0	0	0	> 512	4/2
1	1	1	1	1	1	1	1	1	1	1	1	0	0	0	> 512	4/2
1	1	1	1	1	1	1	1	1	1	1	1	0	0	0	0.5	0.5/0.25

Gentamyci	Spectinom	Tetracyclir	Florfenicol	Sulfafuraz	Sulfameth	Ceftiofur	Ceftazidim	Enrofloxac	Ofloxacin	Meropener	Colistin
Gentamyci	Spectinom	Tetracyclir	Florfenicol	Sulfafuraz	Sulfameth	Ceftiofur	Ceftazidim	Enrofloxac	Ofloxacin	Meropener	Colistin
1	> 512	2	128	128	> 32/608	> 256	4	2	8	⩽0.03	1
0.5	64	128	256	512	> 32/608	1	1	0.5	2	⩽0.03	0.25
0.5	64	128	> 256	> 512	> 32/608	0.5	0.5	0.5	2	⩽0.03	0.5
1	64	64	> 256	> 512	> 32/608	0.5	0.5	0.5	2	⩽0.03	0.5
0.5	64	2	4	> 512	0.5/9.5	0.5	0.5	0.25	1	⩽0.03	4
> 512	> 512	128	> 256	> 512	> 32/608	> 256	16	> 32	> 64	⩽0.03	0.5
1	64	64	256	> 512	> 32/608	0.5	0.5	1	2	⩽0.03	0.5
0.5	64	2	2	> 512	0.5/9.5	0.5	0.5	0.5	1	⩽0.03	4
0.5	64	64	2	8	⩽0.06/1.2	0.5	0.25	0.03	0.06	⩽0.03	0.5
0.5	64	128	> 256	> 512	> 32/608	1	0.5	1	2	⩽0.03	0.5
0.5	64	1	2	32	⩽0.06/1.2	0.5	0.5	0.03	0.12	⩽0.03	0.5
2	> 512	16	2	4	> 32/608	⩽0.12	⩽0.12	1	1	0.5	8
> 512	> 512	128	> 256	> 512	> 32/608	256	32	> 32	> 64	⩽0.03	0.5
> 512	> 512	128	> 256	> 512	> 32/608	> 256	32	> 32	> 64	⩽0.03	0.5
> 512	> 512	128	> 256	> 512	> 32/608	256	16	> 32	> 64	⩽0.03	0.25
64	64	128	2	16	0.125/2.4	1	0.25	0.25	1	⩽0.03	4
> 512	> 512	128	> 256	> 512	> 32/608	128	16	> 32	64	⩽0.03	0.25
0.5	128	1	2	64	⩽0.06/1.2	1	0.5	0.06	0.12	⩽0.03	1
128	64	128	64	16	⩽0.06/1.2	0.5	0.5	0.25	1	⩽0.03	2
64	64	128	2	32	⩽0.06/1.2	1	0.5	0.5	1	⩽0.03	2
64	64	128	2	32	⩽0.06/1.2	0.5	0.5	0.25	0.5	⩽0.03	2
> 512	> 512	128	> 256	> 512	> 32/608	256	32	> 32	> 64	⩽0.03	0.5
64	64	64	2	32	⩽0.06/1.2	0.5	0.5	0.25	0.5	⩽0.03	2
64	64	64	2	16	⩽0.06/1.2	1	0.25	0.25	0.5	⩽0.03	4
64	64	64	2	16	⩽0.06/1.2	0.5	0.5	0.25	0.5	⩽0.03	2
64	64	64	2	16	⩽0.06/1.2	1	0.25	0.25	0.5	⩽0.03	4
64	64	1	2	64	⩽0.06/1.2	0.5	0.5	0.25	0.5	⩽0.03	4
> 512	> 512	128	> 256	> 512	> 32/608	128	16	> 32	> 64	⩽0.03	0.25
0.5	64	64	2	> 512	⩽0.06/1.2	0.5	1	0.5	1	⩽0.03	1
0.5	64	64	2	64	⩽0.06/1.2	0.5	1	0.5	2	⩽0.03	1
0.5	64	64	2	> 512	⩽0.06/1.2	1	0.5	0.5	2	⩽0.03	1
0.5	64	64	2	> 512	⩽0.06/1.2	0.5	0.25	0.5	1	⩽0.03	1
0.5	64	64	2	> 512	⩽0.06/1.2	0.5	0.25	0.5	1	⩽0.03	1
⩽0.25	64	128	2	> 512	0.5/9.5	0.5	0.5	0.5	1	⩽0.03	4
⩽0.25	64	128	2	> 512	0.25/4.8	0.5	0.5	0.5	1	⩽0.03	4
⩽0.25	64	128	2	> 512	0.25/4.8	0.5	0.5	0.5	1	⩽0.03	4
0.5	64	1	2	32	⩽0.06/1.2	0.5	0.5	0.03	0.12	0.25	1
0.5	64	1	2	32	⩽0.06/1.2	0.5	0.5	0.03	0.12	0.12	2

0.5	64	1	2	32	≤0.06/1.2	0.5	0.5	0.03	0.12	≤0.03	2
0.5	64	1	2	32	≤0.06/1.2	0.5	0.25	0.03	0.12	≤0.03	2
0.5	64	1	2	16	≤0.06/1.2	0.5	0.25	0.03	0.12	≤0.03	1
0.5	64	1	2	64	≤0.06/1.2	0.5	0.25	≤0.01	0.12	≤0.03	2
0.5	> 512	1	2	> 512	> 32/608	1	0.25	0.25	0.5	≤0.03	4
0.5	64	1	2	32	≤0.06/1.2	0.5	0.25	0.03	0.12	≤0.03	2
0.5	64	1	2	16	≤0.06/1.2	0.5	0.25	0.03	0.12	≤0.03	1
0.5	64	2	2	32	≤0.06/1.2	0.25	0.25	0.03	0.12	≤0.03	1
0.5	64	1	2	32	≤0.06/1.2	0.25	0.5	0.03	0.12	≤0.03	1
0.5	64	1	2	32	≤0.06/1.2	0.5	0.5	0.03	0.12	≤0.03	1
0.5	64	1	2	32	≤0.06/1.2	0.5	0.5	≤0.01	0.12	≤0.03	2
0.5	64	1	2	16	≤0.06/1.2	0.5	0.25	≤0.01	0.12	≤0.03	1
64	> 512	128	> 256	> 512	> 32/608	256	16	> 32	> 64	≤0.03	0.25
64	> 512	64	256	> 512	> 32/608	> 256	8	> 32	32	≤0.03	0.5
128	> 512	128	> 256	> 512	> 32/608	256	32	> 32	> 64	≤0.03	0.25
32	> 512	128	> 256	> 512	> 32/608	256	16	> 32	> 64	≤0.03	0.5
64	> 512	256	256	> 512	> 32/608	256	16	16	64	≤0.03	0.5
128	> 512	128	> 256	> 512	> 32/608	256	16	> 32	> 64	≤0.03	0.25
16	64	128	> 256	> 512	> 32/608	1	1	1	2	≤0.03	0.5
32	64	128	> 256	> 512	> 32/608	1	0.5	1	4	≤0.03	0.5
32	64	128	> 256	> 512	> 32/608	2	0.5	1	2	≤0.03	0.5
8	64	128	> 256	> 512	> 32/608	1	0.5	0.5	2	≤0.03	0.5
16	64	128	> 256	> 512	> 32/608	1	0.5	0.5	2	≤0.03	0.5
0.5	64	1	4	32	≤0.06/1.2	0.5	0.5	0.03	0.12	≤0.03	2
0.5	64	32	2	> 512	≤0.06/1.2	0.5	0.25	0.5	2	≤0.03	1
0.5	64	64	2	> 512	≤0.06/1.2	0.5	0.25	0.5	1	≤0.03	0.5
32	> 512	128	> 256	> 512	> 32/608	256	32	> 32	> 64	≤0.03	0.5
64	> 512	128	> 256	> 512	> 32/608	256	16	> 32	64	≤0.03	0.5
64	> 512	128	> 256	> 512	> 32/608	256	16	> 32	> 64	≤0.03	0.5
128	> 512	128	> 256	> 512	> 32/608	> 256	16	> 32	> 64	≤0.03	0.5
64	> 512	128	> 256	> 512	> 32/608	256	16	> 32	> 64	≤0.03	0.5
8	64	128	> 256	> 512	> 32/608	1	0.5	1	2	≤0.03	0.5
16	128	128	> 256	> 512	> 32/608	1	0.5	1	2	≤0.03	0.5
32	> 512	128	> 256	> 512	> 32/608	256	16	> 32	> 64	≤0.03	0.5
32	> 512	128	> 256	> 512	> 32/608	0.5	0.25	1	2	≤0.03	0.5
32	> 512	128	> 256	> 512	> 32/608	0.5	1	1	2	≤0.03	0.5
16	> 512	128	> 256	> 512	> 32/608	0.5	0.25	1	2	≤0.03	0.5
32	> 512	128	> 256	> 512	> 32/608	0.5	0.25	1	2	≤0.03	0.5
16	> 512	128	> 256	> 512	> 32/608	0.5	0.25	1	2	≤0.03	0.5
16	> 512	128	> 256	> 512	> 32/608	0.5	0.25	1	1	≤0.03	0.5
32	> 512	128	> 256	> 512	> 32/608	0.5	0.5	1	2	≤0.03	0.5
32	> 512	128	256	> 512	> 32/608	0.5	0.5	1	2	≤0.03	1
0.5	64	64	128	32	≤0.06/1.2	0.5	0.5	0.5	2	≤0.03	0.5

0.5	64	64	128	32	≤0.06/1.2	0.5	0.5	0.5	1	≤0.03	0.5
0.5	64	64	128	16	≤0.06/1.2	0.5	0.5	0.5	2	≤0.03	0.25
0.5	64	64	128	16	≤0.06/1.2	0.5	0.5	0.5	2	≤0.03	0.5
0.5	64	64	128	16	≤0.06/1.2	0.5	0.5	0.5	2	≤0.03	0.5
0.5	256	64	256	> 512	> 32/608	0.5	0.5	0.25	0.5	≤0.03	0.5
0.5	256	64	256	> 512	> 32/608	1	1	0.25	0.5	≤0.03	0.25
0.5	64	64	256	> 512	0.125/2.4	0.5	0.5	2	4	≤0.03	0.5
0.5	256	64	256	> 512	> 32/608	1	0.5	0.25	0.5	≤0.03	0.5
0.5	512	64	256	> 512	> 32/608	4	0.5	0.25	0.5	≤0.03	0.5
0.5	512	64	256	> 512	> 32/608	1	0.5	0.25	0.5	≤0.03	0.5
0.5	256	64	256	> 512	> 32/608	1	0.5	0.25	0.5	≤0.03	0.5
0.5	64	1	2	> 512	≤0.06/1.2	0.5	0.25	0.25	1	≤0.03	1
0.5	512	1	256	> 512	> 32/608	0.25	≤0.12	0.12	0.5	≤0.03	0.5
0.5	256	64	256	> 512	> 32/608	0.5	0.5	0.25	0.5	≤0.03	0.5
1	> 512	1	256	32	> 32/608	256	4	2	8	≤0.03	0.5
1	256	64	256	> 512	> 32/608	0.5	0.5	0.25	0.5	≤0.03	0.25
0.5	32	128	4	> 512	≤0.06/1.2	1	0.5	2	4	≤0.03	0.5
64	> 512	128	256	> 512	> 32/608	> 256	16	> 32	64	≤0.03	0.5
1	32	32	128	> 512	≤0.06/1.2	0.25	0.5	0.25	1	≤0.03	0.5
0.5	16	128	4	> 512	0.125/2.4	0.5	0.5	1	2	≤0.03	0.25
0.5	16	64	4	> 512	≤0.06/1.2	0.5	0.5	1	2	≤0.03	≤0.12
64	> 512	4	> 256	> 512	> 32/608	> 256	16	> 32	32	≤0.03	1
64	> 512	128	> 256	> 512	> 32/608	> 256	32	> 32	32	≤0.03	1
0.5	16	64	256	> 512	0.125/2.4	0.25	0.25	0.5	1	≤0.03	2

Table S4. Antimicrobial-resistant patterns of 105 *Salmonella* isolates.

ID	Antibiotic-resistant pattern	Number	Percentage%
1	GEM-SPT-AMP--CEF-CAZ--FFC-SF-SXT-ENR-OFL-TET	20	19.05
2	-	15	14.29
3	SPT-AMP-FFC-SF-SXT-TET	8	7.62
4	AMP-SF-TET	6	5.72
5	FFC-SF-SXT-TET	5	4.77
6	GEM-SPT-AMP-FFC-SF-SXT-TET	5	4.77
7	FFC-TET	5	4.77
8	GEM-AMP-TET	4	3.81
9	GEM-AMP-FFC-SF-SXT-TET	4	3.81
10	GEM-SPT-AMP-A/C-FFC-SF-SXT-TET	4	3.81
11	GEM-AMP-TET-CL	3	2.86
12	SPT-AMP-CEF-FFC--SXT-ENR	2	1.91
13	AMP-SF-CL	2	1.91
14	AMP-SF-TET-CL	2	1.91
15	AMP-FFC-SF-SXT-TET	2	1.91
16	FFC-SF-TET	2	1.91
17	SF-TET	2	1.91
18	TET	1	0.96
19	SPT-AMP-A/C-SXT-TET-CL	1	0.96
20	SPT	1	0.96
21	GEM-AMP-FFC-TET	1	0.96
22	GEM-AMP-CL	1	0.96
23	AMP-TET	1	0.96
24	SF-TET-CL	1	0.96
25	SPT-AMP-SF-SXT-CL	1	0.96
26	GEM-SPT-AMP-CEF-FFC-SF-SXT-ENR-OFL-TET	1	0.96
27	AMP-FFC-SF--ENR-TET	1	0.96
28	SF	1	0.96
29	SPT-AMP-FFC-SF-SXT	1	0.96
30	SF-ENR-TET	1	0.96
31	GEM-SPT-AMP-CEF-CAZ-FFC-SF-SXT-ENR-OFL	1	0.96

Table S5. the level of agreement between phenotypic-genotypic acquired antimicrobial resistance.

antibiotics	concordance rate (%)	A (%)	B (%)	C (%)	D (%)
Gentamycin	83.82	36.20	47.62	10.48	5.72
Spectinomycin	83.82	37.15	46.67	10.48	5.72
Ampicillin	91.44	61.91	29.53	2.86	5.72
Amoxicillin-Clavulanic acid	84.77	3.81	80.96	14.29	0.96
Ceftiofur	93.34	20.00	73.34	3.81	2.86
Ceftazidime	90.49	17.15	73.34	6.67	2.86
Meropenem	100.00	0.00	100.00	0.00	0.00
Florfenicol	89.54	52.39	37.15	3.81	6.67
Sulfafurazole	77.15	38.1	39.05	8.58	14.29
Sulfamethoxazole-Trimethoprim	91.44	46.67	44.77	2.86	5.72
Enrofloxacin	69.53	19.05	50.48	24.77	5.72
Ofloxacin	65.72	15.24	50.48	28.58	5.72
Tetracycline	91.44	69.53	21.91	1.91	6.67
Colistin	89.53	0.00	89.53	0.00	10.48

A: both genotype and phenotype were positive

B: both genotype and phenotype were negative

C: only genotypes of acquired AMR genes are present

D: only AMR phenotypes are present

Table S6. MIC breakpoints for *Salmonella* in this study

Antibiotics	MIC breakpoints (μg/mL)		
	S	I	R
Ampicillin	⩽8	16	⩾32
Amoxicillin-Clavulanic acid	⩽8/4	16/8	⩾32/16
Ceftiofur	⩽2	4	⩾8
Ceftazidime	⩽4	8	⩾16
Meropenem	⩽1	2	⩾4
Spectinomycin	⩽32	64	⩾128
Gentamycin	⩽4	8	⩾16
Tetracycline	⩽4	8	⩾16
Florfenicol	⩽4	8	⩾16
Sulfafurazole	⩽256	-	⩾512
Sulfamethoxazole-Trimethoprim	⩽2/38	-	⩾4/76
Enrofloxacin	⩽0.5	0.25~1	⩾2
Ofloxacin	⩽0.12	0.25-1	⩾2
Colistin	⩽2	-	⩾4

S, susceptible; I, intermediate; R, resistant; “-”, Indicates that there is no corresponding criterion.
